# Effects of turn-structure on folding and entanglement in artificial molecular overhand knots†

**DOI:** 10.1039/d0sc05897a

**Published:** 2020-12-08

**Authors:** Yiwei Song, Fredrik Schaufelberger, Zoe Ashbridge, Lucian Pirvu, Iñigo J. Vitorica-Yrezabal, David A. Leigh

**Affiliations:** Shanghai Engineering Research Center of Molecular Therapeutics and New Drug Development, School of Chemistry and Molecular Engineering, East China Normal University Shanghai 200062 PR China david.leigh@manchester.ac.uk; Department of Chemistry, University of Manchester Oxford Road Manchester M13 9PL UK

## Abstract

The length and constitution of spacers linking three 2,6-pyridinedicarboxamide units in a molecular strand influence the tightness of the resulting overhand (open-trefoil) knot that the strand folds into in the presence of lanthanide(iii) ions. The use of β-hairpin forming motifs as linkers enables a metal-coordinated pseudopeptide with a knotted tertiary structure to be generated. The resulting pseudopeptide knot has one of the highest backbone-to-crossing ratios (BCR)—a measure of knot tightness (a high value corresponding to looseness)—for a synthetic molecular knot to date. Preorganization in the crossing-free turn section of the knot affects aromatic stacking interactions close to the crossing region. The metal-coordinated pseudopeptide knot is compared to overhand knots with other linkers of varying tightness and turn preorganization, and the entangled architectures characterized by NMR spectroscopy, ESI-MS, CD spectroscopy and, in one case, X-ray crystallography. The results show how it is possible to program specific conformational properties into different key regions of synthetic molecular knots, opening the way to systems where knotting can be systematically incorporated into peptide-like chains through design.

## Abstract 2

Spacers linking 2,6-pyridinedicarboxamide units influence the tightness of the corresponding lanthanide-coordinated overhand knot. β-Hairpin forming motifs generate a metal-coordinated pseudopeptide with a knotted tertiary structure.

## Graphical abstract

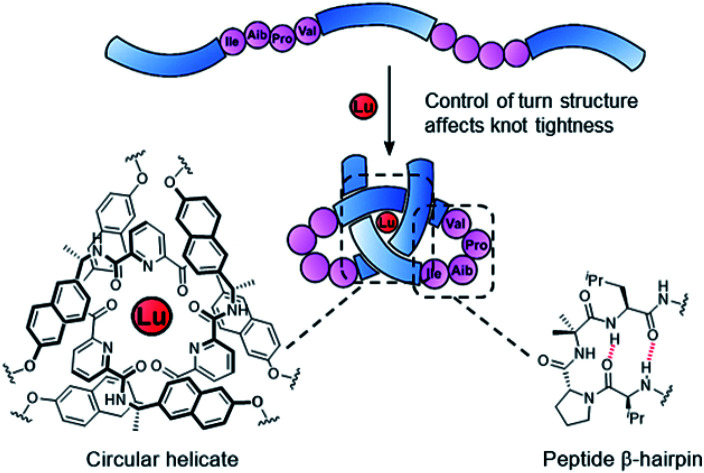

## Introduction

Entangled strands occur in nature at all length scales, from the cm-scale knots in bird nests to μm-sized chromatin bundles and circular DNA and proteins that span a few nm in each dimension.^1–7^ At the molecular level, knotting a strand alters characteristics such as size and shape,^8,9^ stability,^10^ mechanical properties^11–14^ and the expression of chirality.^15^ However, the underlying basis for such effects is not always clear, partly due to a lack of studies in which the knot backbone is systematically varied.^16,17^

Synthetic molecular knots are generally constructed through the self-assembly of multiple building blocks to generate the requisite crossing pattern, followed by multiple connections of the building block ends to form the strand entanglement.^1,18–22^ Biology takes a different approach, programing tertiary structure into proteins by encoding noncovalent interactions, stereoelectronic effects and conformational preferences into the strand primary structure, which then folds through the locally organised secondary structures (*e.g.* β-turns *etc.*) into the functional shape.^3,23,24^ Around 2% of proteins have entangled (‘knotted’§§Protein ‘knots’ invariably do not have a closed-loop backbone of covalent bonds and are therefore technically topologically trivial, *i.e.* the knotted conformation can be untangled through a specific series of bond rotations. However, as the knotted conformation either represents a thermodynamic minimum or is persistent over a long time scale, it is generally useful to consider their characteristics in a similar manner to topologically non-trivial, closed-loop knots. See also ESI Section S9.†) tertiary structures,^25^ with significant variations in backbone-to-crossing ratio (BCR), the positioning of the knotted region of the protein (deep or shallow), and its topology (*e.g.* 3_1_, 4_1_, 5_2_, 6_1_*etc.*). In contrast, synthetic molecular knots generally have short, well defined, knotted regions that are much tighter and possess high degrees of symmetry.^26–43^ Investigating systematic variations in knot structure may aid the understanding of how molecular knotting impacts properties and, ultimately, how it might prove useful for practical applications.^44–49^

Folding of a single strand can, in principle, allow access to entanglements with lower degrees of symmetry than the assembly of multiple building blocks.^50–54^ However, controlling folding is challenging because the design criteria for restricting conformational entropy to achieve knot formation is likely to be strict. One reason is that folding into one precise topologically non-trivial architecture will generally compete with others or a collapse into non-entangled species. Thus far, there are no methods to create knots with high BCR ratios (loose knots) and limited examples of making unsymmetrical knots by design.^54^ We are interested in approaching such goals by exploring strand compositions (primary structure) that give rise to particular local turns and crossing points (secondary structure) and learning how these can be controlled or encouraged to generate larger, entangled, strand arrangements (tertiary structure).^55,56^

**Fig. 1:**
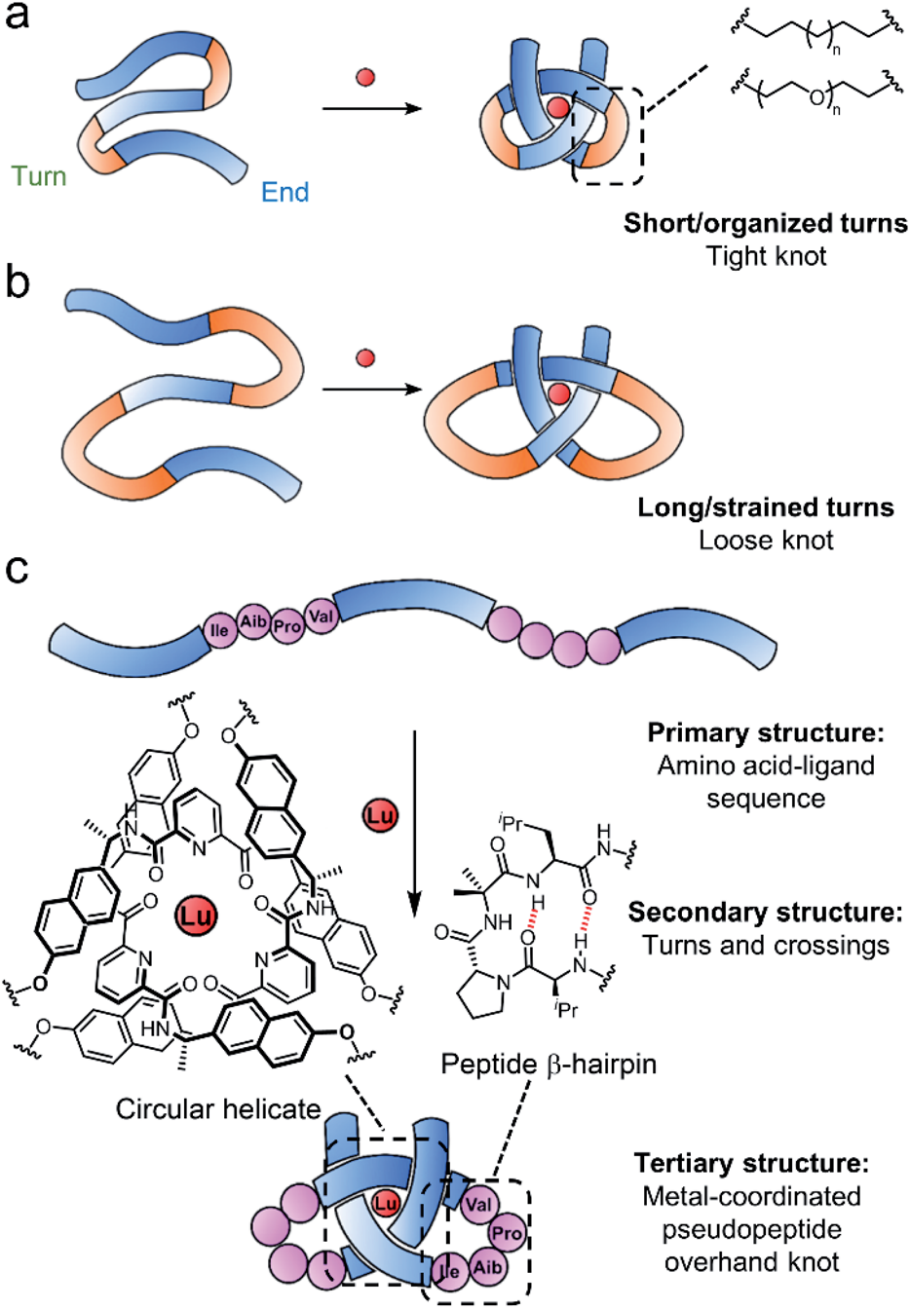
Tight (a) and loose (b) overhand knot folding in the presence of lanthanide ions (red sphere) in a synthetic system. Blue regions correspond to a 2,6-pyridinedicarboxamide unit, orange regions to a turn sequence. (c) Folding of a lanthanide-coordinated pseudopeptide overhand knot with β-hairpin peptide turns.

Tritopic-2,6-pyridinedicarboxamide ligands have been developed that fold into an open trefoil (3_1_) overhand knot conformation in the presence of lanthanide(iii) ions.^9,15,46,54,57^ Here we investigate how these strands can be tied into both very tight and relatively loose overhand knots, and whether the size of the knot backbone can be significantly increased when amino acids that code for a complementary secondary structure are incorporated into the crossing-free turn region of the strand. Unlike conventional lanthanide–peptide assemblies,^58,59^ which typically have dynamic and somewhat poorly-defined coordination spheres, the metal-coordinated pseudopeptide^60–65^ overhand knot maintains the lanthanide ion^66,67^ located in a specific region without interference from other parts of the folded strand (Fig. 1).

## Results and discussion

### Knot tightness in synthetic models

Folding of a tris(2,6-pyridine dicarboxamide) strand to coordinate to a lanthanide(iii) ion restricts the conformational freedom of the strand, resulting in the local environment of many backbone atoms changing significantly. In previous tris(2,6-pyridine dicarboxamide)-lanthanide(iii) trefoil knots,^57,68^ knotting caused characteristic changes observable by ^1^H NMR spectroscopy and circular dichroism. The knot BCR (*i.e.* how many backbone atoms the core knotted region is composed of) is a measure of entanglement tightness used as a convenient indicator in both synthetic and biological systems.^16,69,70^ Most metal-template synthetic knots feature linkers chosen to maximize folding and ring-closing efficiency. When the linkers are too short, a strand cannot fold into a knot due to the strained geometry around the metal centre. When the linkers are too long, misfolding can occur by favouring conformations where loop threading does not occur.¶¶Modelling with CPK models suggests that misfolding to access a closed-loop conformation can only occur from a bridging length of at least 14 sp^3^-atoms per linker, given the saturated coordination sphere of the lutetium(iii) ion. The present study is concerned with exploring looser knots (larger BCR) to assess how entanglement tightness affects folding.^16^

**Fig. 2:**
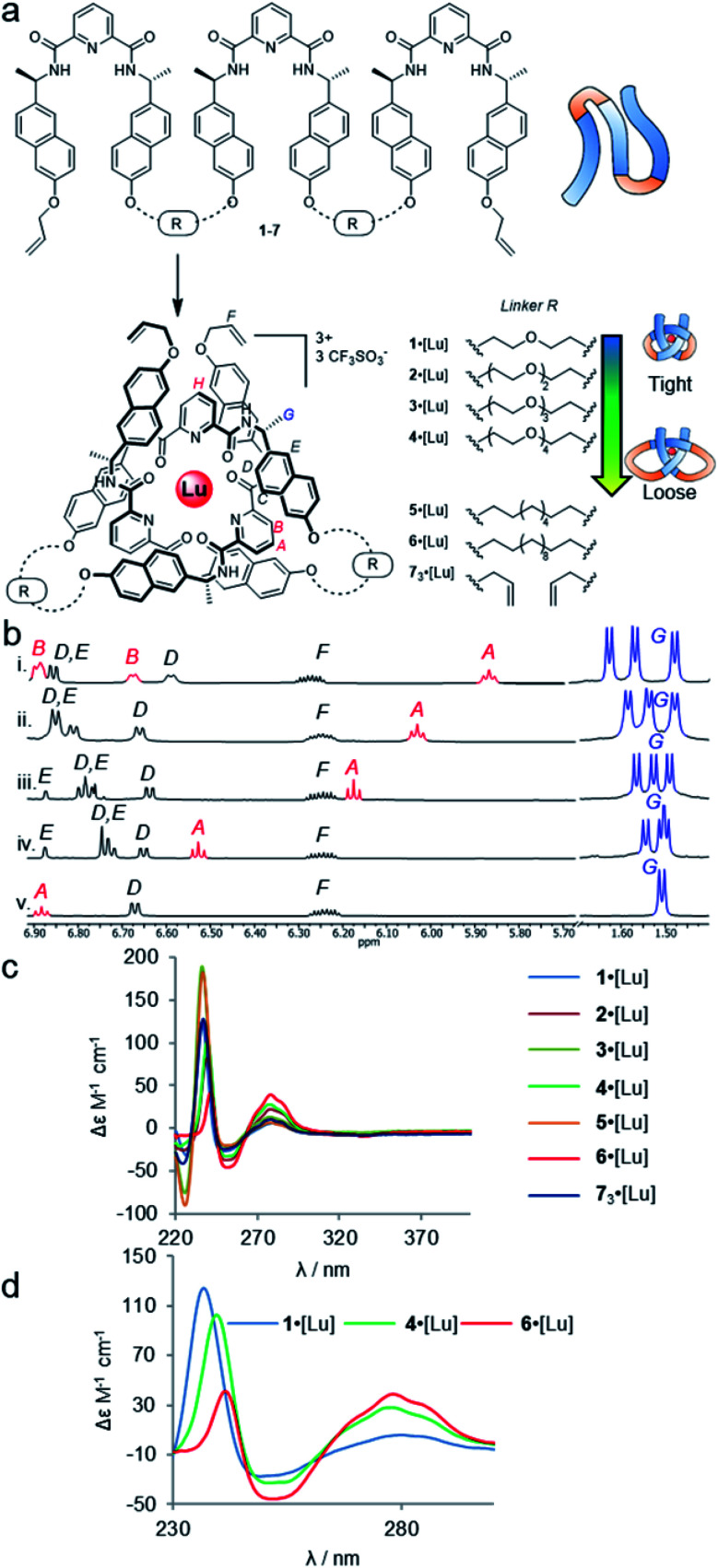
Effects of entanglement tightness in overhand knots. (a) Structure of knots 1–6·[Lu] and circular helicate 7_3_·[Lu]. Reagents and conditions: Lu(CF_3_SO_3_)_3_, MeCN, 80 °C, 16–24 h. (b) Partial ^1^H NMR spectra (600 MHz, 298 K, MeCN-*d*_3_) of overhand knots and helicates (i) 1·[Lu], (ii) 2·[Lu], (iii) 3·[Lu], (iv) 4·[Lu] and (v) 7_3_·[Lu]. For full spectral assignments, see ESI.† (c) Overlaid circular dichroism spectra of overhand knots 1–6·[Lu] and circular helicate 7_3_·[Lu] (MeCN), normalised for absorbance. (d) Expanded region of CD spectra of 1·[Lu], 4·[Lu] and 6·[Lu], showing correlations between chiral expression and tightness of the core entanglement around the lanthanide ion.

Accordingly, we varied linker lengths in the turn region with both poly(ethylene glycol) (PEG) based spacers and aliphatic chains (Fig. 2a). Although these chains only differ by changing every third –CH_2_– group in the crossing-free turn region for an O atom, this can have a profound effect on the conformation of the turn required by the chain: –OCH_2_CH_2_O– units are subject to stereoelectronic effects that favour gauche rather than anti-dihedral angles for the chain.^32^ The absence of these hydrogen atoms also reduces unfavourable 1,3-diaxial steric clashes as the chain twists to form the turn, generating both van der Waals and Pitzer strain.

The synthesis of tritopic molecular strands 1–6 and monotopic ligand 7 was carried out as outlined in the ESI.† Compounds 1–4 incorporate two-to-five polyethylene glycol (PEG) repeat units in each of the two linkers in their main chains, while compounds 5 and 6 use octyl and dodecyl linkers. Each ligand strand was then treated with Lu(CF_3_SO_3_)_3_ in MeCN at 80 °C for 16–24 h in order to induce knotting (Fig. 2a), in each case leading to metal complexation as evidenced by electrospray ionization mass spectrometry (ESI-MS) and ^1^H NMR spectroscopy (see ESI†). DOSY-NMR analysis confirmed that the molecules are single species (spectra S47–S53).

The ^1^H NMR spectra of each of the PEG-linked lanthanide complexes 1–4·[Lu] in MeCN-*d*_3_ were consistent with knotted conformations (Fig. 2b). There is significant correlation between the apparent tightness of each knot and the corresponding solution spectra. Among the most pronounced changes observed upon lanthanide(iii) ion addition are the shifts in the protons H_A_ and H_B_ of the distal pyridine units. The enforced proximity of the electron-rich naphthol units and electron-poor pyridine dicarboxamide units in the knotted conformation increases the π–π stacking leading to upfield shifts due to enhanced proton shielding.

Comparison of the ^1^H NMR data for 1–4·[Lu] shows a correlation between linker length and the extent of the ^1^H NMR spectral shift (Δ*δ*) for proton H_A_ (Fig. 2b). Overhand knot 1·[Lu] only has a five-atom-length bridge (according to molecular modelling the shortest turn distance possible that can still fold into a knotted conformation) and the aromatic units are forced into a tight stacking motif, as evidenced by a Δ*δ* 2.4 ppm for H_A_. The value decreases stepwise with increasing chain length: overhand knot 4·[Lu] has a linker length of 14 atoms and a Δ*δ* for H_A_ of only 1.6 ppm. In comparison, the coordinated circular helicate without connected linkers, 7_3_·[Lu], which does not need to pay an entropic penalty for strand folding and has conformational flexibility of the aromatic ring systems unrestricted by being part of a closed loop, has a Δ*δ* of 1.3 ppm. The trends observed for proton H_A_ also follow for other shielded protons, including the methyl protons, H_G_. The difference in environment of the six methyl groups is strongly expressed when the protons are held in close proximity to the helically chiral crossing region of the knot. The tighter the knot, the greater the shifts observed, from a single set of coincident doublets in 7_3_·[Lu] to three distinct doublets that span a 0.2 ppm range in the tightest overhand knot, 1·[Lu] (Fig. 2b).

The trend for the downfield shifts of the central 2,6-pyridine dicarboxamide unit differs substantially from that of the two peripheral units. Signal H_H_ occurs between 7.0–7.1 ppm for all of the knots, with no correlation in chemical shift to knot tightness. This supports the notion that the observed tightening effects result from the turn structures of the linkers, and also explains why there is little difference in closing efficiency when the overhand knots are covalently captured through ring-closing metathesis.||||Ring-closing olefin metathesis of overhand knots 2·[Lu], 3·[Lu] and 5·[Lu] using the conditions reported in ref. 46 generated the corresponding closed-loop trefoil knots in 80–90% yield.

In contrast to PEG-linked strands 1–4, aliphatic chain-bridged strands 5 and 6 do not benefit from turn-inducing –OCH_2_CH_2_O– gauche interactions, making the bend in the strand required by the knot less favourable. This is reflected in the NMR spectra upon lanthanide-induced knotting in MeCN-*d*_3_. For strand 5, overhand knot 5·[Lu] folds readily, but shows smaller Δ*δ* values than 2·[Lu] with the same number of bridging atoms. This suggests there is some disruption of the π–π interactions that hold the knot core together to minimize strain associated with folding alkyl chains into eclipsed conformations.^71^ The longer strand, 6, folds less effectively than the other strands and produces an insoluble, presumably oligomeric, byproduct as well as the knot. In addition to the Pitzer strain in this system, larger alkyl chain folds can experience transannular Prelog-type strain, which may also contribute to disfavouring the knotted conformation.^72,73^ The Δ*δ* value (1.4 ppm) of proton H_A_ in 6 is smaller than for the PEG-linked knots of comparable linker length, suggesting that the π systems are forced apart to relieve strain in the turn region.

**Scheme 1:**
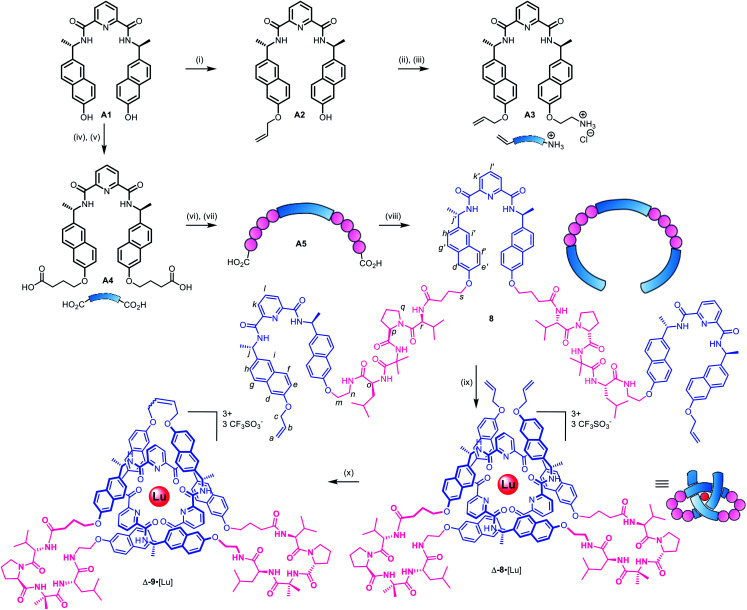
Synthetic route to pseudopeptide ligand 8 and its folding into a pseudopeptide overhand knot through lutetium(iii) complexation. Reagents and conditions: (i) NaOH (s), allyl bromide, DMF, 60 °C, 46%; (ii) BocNHCH_2_CH_2_OTs, Cs_2_CO_3_, DMF, 80 °C, 98%; (iii) HCl, 1,4-dioxane (4 M), quant; (iv) ethyl-4-bromobutyrate, Cs_2_CO_3_, DMF, r.t., 81%; (v) NaOH, EtOH/H_2_O/THF (1 : 1 : 1), 40 °C, quant; (vi) BnO-Val-D-Pro-Aib-Ile-NH_2_, HATU, DIPEA, DMF, 67%; (vii) Pd(OH)_2_/C, MeOH, 35 °C; (viii) A3, HATU, DIPEA, DMF, 5 h, 58% over two steps; (ix) Lu(CF_3_SO_3_)_3_, MeCN, MW (80 W, 80 °C), 8 h, 97%. (x) Hoveyda–Grubbs 2nd generation catalyst, CH_2_Cl_2_/MeNO_2_ 3 : 2, 50°C, 24 h, 90%.

The overhand knots also differ significantly in terms of their circular dichroism spectra (Fig. 2c). The helically chiral arrangement of the chromophores around the Lu(iii) ion results in three exciton coupling maxima around 235, 250 and 285 nm. The relative intensity of the Cotton effects qualitatively correlates with the entanglement tightness, and there is a clear red shift in the exciton couplings upon going from tighter to looser knots (Fig. 2d).

**Fig. 3:**
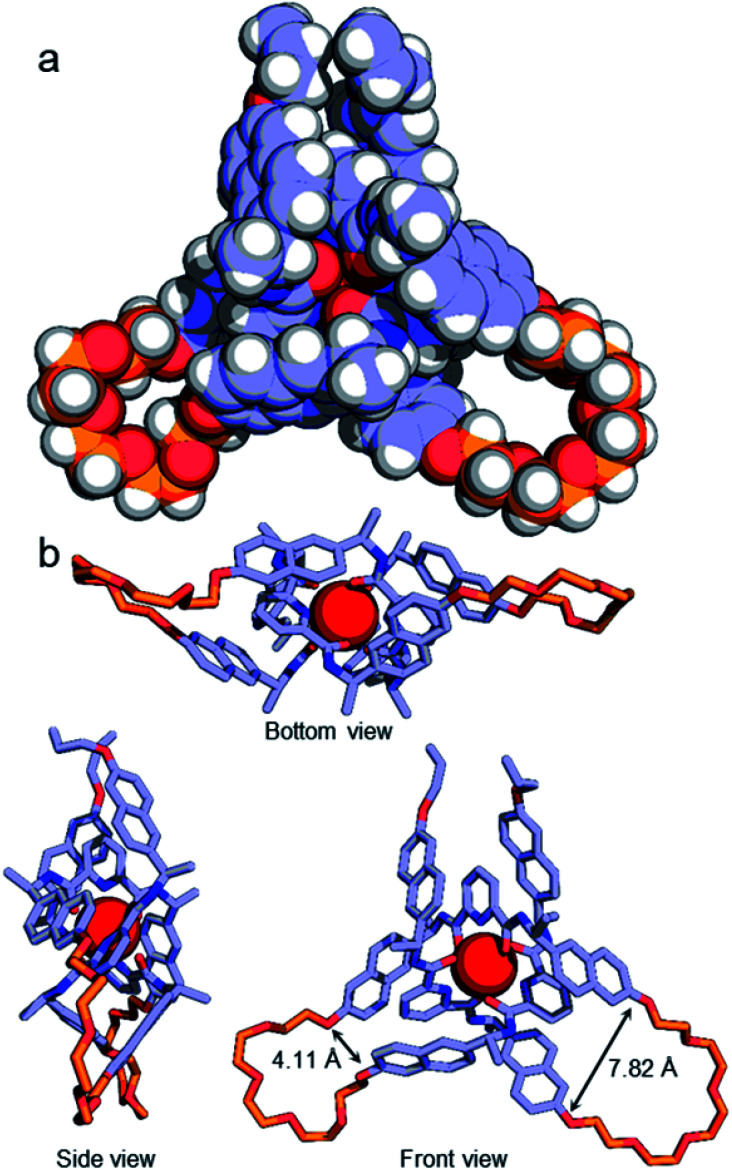
(a) X-ray crystal structure of Λ-4·[Lu], shown in space-filling representation. Selected metal–donor atom bond lengths (Å): Lu–O 2.32(2) × 4, 2.34(2), 2.30; Lu–N 2.46(2), 2.43, 2.44(1). (b) Illustrative stick representations of the crystal structure from several angles. Hydrogen atoms and counteranions omitted for clarity.

Although no examples of X-ray crystal structures of chiral overhand knots have previously been reported, single crystals suitable for X-ray diffraction analysis were grown of 4·[Lu] by slow diffusion of diethyl ether into an acetonitrile solution. In the X-ray crystal structure of 4·[Lu] (Fig. 3) the ligand wraps around the lutetium(iii) ion with the metal in a trigonal-prismatic coordination geometry. The Lu–O (2.30/2.31/2.34 Å) and Lu–N (2.43/2.44/2.46 Å) distances are in the expected ranges for lanthanide-2,6-pyridinedicarboxamide complexes. The structure of the knot is significantly distorted from C_2_-symmetry, with the looped regions having pocket-spanning naphthol O–O distances of 7.82 Å in one turn region and 4.11 Å in the other (Fig. 3b). Lanthanide coordination complexes are labile in solution, so the solid-state asymmetry is consistent with the temperature-dependence of the ^1^H NMR spectra apparent with this class of knots, where high temperatures are often necessary to obtain well-resolved ^1^H NMR spectra.^45,54,57^

### A metal-coordinated pseudopeptide overhand knot

As the constitution of the turn region significantly affects the structure around the crossing points, we explored preorganizing the turns needed for tight knots to form. This is equivalent to adding a persistent ‘turn motif’ to the linker sequence. In globular proteins, two-residue reverse turns (β-turns) are often present in loop regions and can serve important biological functions such as regulating protein–protein and protein–nucleic acid interactions.^74–78^ Type II′ β-turns composed of ProGly, ProAla or ProAib sequences are particularly robust, and when incorporated in peptide sequences reduce conformational flexibility.^79,80^ We incorporated IleProAibVal sequences to connect the coordinating pyridinedicarboxamide units. The hydrophobic isoleucine and valine units were chosen to optimise the solubility of the strand in organic solvents.

Synthesis of pseudopeptide ligand 8 started from previously reported building block A1 (Scheme 1). Desymmetrization with allyl bromide provided building block A2, which was appended with a primary amine moiety in two steps in 80% yield to yield A3. Dual carboxylic acid moieties were attached to A1 in two steps by Williamson ether synthesis followed by hydrolysis to produce compound A4 in 81% yield. Meanwhile, an *O*-benzyl-protected *N*-to-*C* IleProAibVal peptide sequence was obtained *via* conventional peptide synthesis methods (see ESI†). Coupling of this peptide sequence to A4 proceeded readily with the HATU peptide coupling reagent to deliver the extended strand A5 in 67% yield, followed by removal of the benzyl groups by hydrogenation and a final peptide coupling with A3 to yield ligand 8 in 78% yield.

**Fig. 4:**
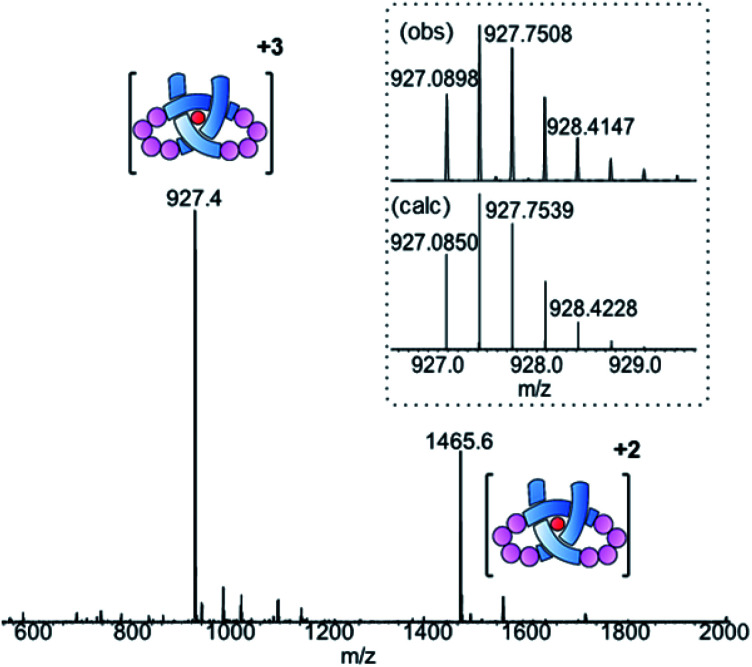
ESI-MS (positive mode) of pseudopeptide overhand knot complex 8·[Lu]. Inset: isotopic distribution for [M]^3+^ signal from HR-MS.

Ligand 8 has a 113-atom backbone, incorporating 14 amide bonds, making it one of the longest synthetic molecular strands to be tested for overhand knot folding. Attempts to coordinate the strand to Lu(CF_3_SO_3_)_3_ under the previously developed conditions (MeCN, 80 °C) gave no reaction. The poor solubility of 8 likely impeded reactivity but we explored alternative solvents and reaction conditions without success. However, running the complexation experiment in MeCN under microwave irradiation led to smooth, apparently quantitative, complex formation as evidenced by ESI-MS (*m*/*z*8·[Lu]^3+^ 927.4, 8·[Lu][CF_3_SO_3_]^2+^ 1465.6, see Fig. 4 and spectrum S54 for DOSY). High-resolution mass spectrometry produced a fragment pattern identical to the computationally-predicted spectrogram for a 1 : 1 ligand–Lu complex (Fig. 4, inset).

**Fig. 5:**
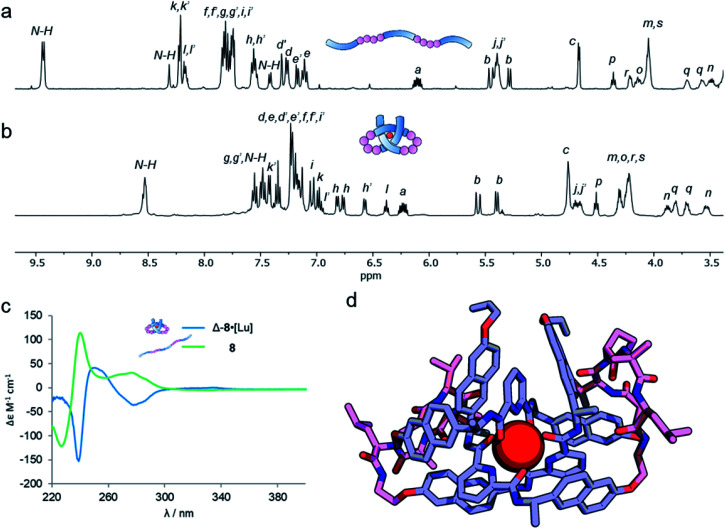
A metal-coordinated pseudopeptide overhand knot. (a) ^1^H NMR spectrum (600 MHz, 298 K, CDCl_3_) of strand 8. (b) ^1^H NMR spectrum (500 MHz, 298 K, MeCN-*d*_3_) of metallopeptide overhand knot Δ-8·[Lu]. (c) Overlaid circular dichroism spectra of strand 8 (green, CH_2_Cl_2_) and metal-coordinated pseudopeptide overhand knot Δ-8·[Lu] (blue, MeCN), normalized for absorbance. (d) Molecular model of Δ-8·[Lu], geometry-optimized with B3LYP-D3/6-311g(d,p)//6-31g(d), with dispersion correction, in MeCN.

Analysis of the overhand knot formation by ^1^H NMR spectroscopy confirmed the entangled nature of the complex (Fig. 5a and b). The chemical shifts for the H_*j*_ protons change significantly as a result of complexation, and the shifts in H_*k*_, and H_*i*_ confirm the π–π interactions present in the structure. Protons H_*h*_ belonging to the peripheral naph-7 positions are invariant in compounds 1–6·[Lu], but split into several signals for 8·[Lu] as a result of the linker chirality. Finally, the key pyr-4 position H_*l*_ appears at 6.36 ppm. The magnitude of this shift is significant, as it shows that the β-hairpins have preorganized the turn in a manner that aids knot formation, pinching the structure to achieve closer inter-aromatic distances and resulting in a tighter overhand knot. For comparison (Table 1), the ‘pinching effect’ (the enforced conformation of the turn structure around the pyridyl unit) achieved with the 21-atom long β-hairpin peptide spacers is equivalent to a PEG-linker with 13 atoms or an aliphatic linker with 10 CH_2_ groups, highlighting how turn-inducing secondary structural elements can promote formation of a knotted tertiary structure.

**Table 1:** Comparison of key ^1^H NMR shifts in overhand knots of varying tightness^a^

Compound	BCR^b^	δH_Pyr-4_ (ppm)	ΔδH_Me_ (ppm)
1·[Lu]	26	5.87	0.19
2·[Lu]	28	6.06	0.14
3·[Lu]	30	6.20	0.10
4·[Lu]	32	6.56	0.07
5·[Lu]	28	6.25	0.12
6·[Lu]	31	6.70	0.04
7_3_·[Lu]	N/A	6.91	0.02
8·[Lu]	37	6.36	0.10

a ^1^H NMR spectroscopy (600 MHz, 298 K, MeCN-*d*_3_).

b Based on knot backbone from peripheral naphthalene *O*-substituent, see ESI Section 9.

The pseudopeptide strand and corresponding metal-coordinated knot were also analysed by circular dichroism (Fig. 5c). Strong Cotton effects and red shifts of exciton coupling maxima upon knotting indicate that the entanglement is localized around the pyridine dicarboxamide units and that the chromophores occupy a helical conformation around the metal ion. The presence of the chiral linkers does not affect the handedness of the resulting knotted conformation as both wavelength and magnitude of the exciton coupling maxima are similar to the overhand knots with achiral linkers (Fig. 2c). By comparing Fig. 5a and b it is apparent that the environment of some protons in ligand 8 changes significantly upon knotting. This is in line with molecular modeling (geometry-optimized with DFT at the B3LYP-D3/6-311g(d,p)//6-31g(d) level of theory), which indicates that the β-hairpins pinch the knotted core tightly together (Fig. 5d). This imposes an inter-atomic naphthol O–O distance of 4.67 and 5.18 Å, indicating tighter pinching than in PEG-based overhand knot 4·[Lu]. The model also indicates the β-turns themselves fold inwards towards the knotted core to maximize attractive dispersion interactions between the greasy Ile and Val side chains and the hydrophobic knot core.

The metal-coordinated pseudopeptide overhand knot 8·[Lu] was closed by ring closing olefin metathesis with Hoveyda–Grubbs 2nd generation catalyst to produce trefoil knot Δ-9·[Lu], which was isolated in 90% yield (Scheme S12†).^46^

## Conclusions

Unlike the knots in proteins and DNA, which commonly span hundreds of atoms, most synthetic molecular knots are small and tight, typically comprising ∼30 atoms per crossing (BCR). Here we have observed direct correlation between the tightness of the central entanglement section and the structure of crossing-free turn regions. A pseudopeptide knot system, with a BCR of 37, demonstrates that with appropriate linker motifs even long strands with multiple degrees of freedom can fold into well-defined entangled tertiary structures. We anticipate that these concepts will be adaptable to biocompatible knots and useful in incorporating knotting motifs into larger biopolymers, such as proteins.

## Conflicts of interest

The authors declare no conflicts of interests.

## Supplementary Material

SC-012-D0SC05897A-s001

SC-012-D0SC05897A-s002
