# Supplementary material for: Effects of turn-structure on folding and entanglement in artificial molecular overhand knots
Source: Chem Sci. 2020 Dec 8;12(5):1826–33. doi: 10.1039/d0sc05897a (PMC8179330; doi:10.1039/d0sc05897a)
Supplement: SC-012-D0SC05897A-s001 [file SC-012-D0SC05897A-s001.pdf]

Electronic Supplementary Material (ESI) for Chemical Science.
This journal is © The Royal Society of Chemistry 2020

Effects of turn-structure on folding and entanglement in artificial molecular overhand knots

Yiwei Song[a], Fredrik Schaufelberger[b], Zoe Ashbridge[b], Lucian Pirvu[b], Iñigo J. Vitorica-Yrezabal[b] and David A. Leigh[a][b]*

[a] School of Chemistry and Molecular Engineering, East China Normal University, 200062 Shanghai, China.
[b] Department of Chemistry, University of Manchester, Oxford Road, Manchester, M13 9PL, UK.

*E-mail: david.leigh@manchester.ac.uk

-Supporting Information-

Table of Contents

S1. Abbreviations S3

S2. General experimental S4

S3. Reaction schemes S6

S4. Experimental procedures S12

S5. Mass spectra S33

S6. NMR spectra S41

S7. Circular dichroism and absorption spectra S68

S8. Crystallography S77

S9. BCR calculations S79

S10. References S80

Appendix. XYZ coordinates for **8**•[Lu] S81

S1. ABBREVIATIONS

BCR backbone-to-crossing ratio; Bn benzyl; Boc *tert*-butyloxycarbonyl; COSY correlation spectroscopy; DEPT distortionless enhancement by polarization transfer; DIPEA *N,N*-diisopropylethylamine; DMF *N*,*N*-dimethylformamide; DMSO dimethylsulfoxide; EDC 1-Ethyl-3-(3-dimethylaminopropyl)carbodiimide; HATU hexafluorophosphate azabenzotriazole tetramethyl uronium; HMBC heteronuclear multiple-bond correlation; HOBt hydroxybenzotriazole; HRMS high-resolution mass spectrometry; HSQC heteronuclear single quantum coherence; MALDI-TOF matrix assisted laser desorption/ionization time-of-flight; MeCN acetonitrile; NMR nuclear magnetic resonance; RT room temperature; THF tetrahydrofuran; TLC thin layer chromatography.

S2. GENERAL EXPERIMENTAL

Unless stated otherwise, reagents were obtained from commercial sources and used without purification. Reactions were carried out in anhydrous solvents under an N_2 atmosphere. Anhydrous solvents were obtained by passing the solvent through an activated alumina column on a Phoenix SDS (solvent drying system; JC Meyer Solvent Systems, CA, USA). Compounds **S1**[1], **S2**[2], **S3**[3], **S4**[3], **S5**[4], **S14**[5], **S15**[6], **S17**[6], **S18**[7], **S20**[8], **A1**[5], **A2**[6], **2**[6], **5**[8] and **2**•[Lu][6] were synthesized as previously described. ^{1}H NMR spectra were recorded on a Bruker Avance III with an Oxford AS600 magnet equipped with a cryoprobe [5mm CPDCH ^{13}C-^{1}H/D] (600 MHz) or a Bruker Ascend 500 MHz spectrometer. Chemical shifts are reported in parts per million (ppm) from high-to-low frequency using the residual solvent peak as the internal reference ($CDCl_3$ = 7.26 ppm, $(CD_3)_2SO$ = 2.50 ppm, CD_3CN = 1.94 ppm). All ^{1}H resonances are reported to the nearest 0.01 ppm. The multiplicity of ^{1}H signals are indicated as: s = singlet; d = doublet; t = triplet; q = quartet; m = multiplet; br = broad; app = apparent. Coupling constants (*J*) are quoted in Hz and reported to the nearest 0.1 Hz. Where appropriate, averages of the signals from peaks displaying multiplicity were used to calculate the value of the coupling constant. ^{13}C NMR spectra were recorded on the same spectrometer with the central resonance of the solvent peak as the internal reference (CD_3CN = 118.26 ppm, $CDCl_3$ = 77.16 ppm, and $(CD_3)_2SO$ = 39.52 ppm). All ^{13}C resonances are reported to the nearest 0.01 ppm. DEPT, COSY, HSQC and HMBC experiments were used to aid structural determination and spectral assignment. Fully characterized compounds were chromatographically homogeneous. DOSY measurements were performed using the standard Bruker pulse program dstebpgp3s. Smoothed square gradients were used with a total duration of 1.6 ms. The gradient recovery delay was 200 µs, and diffusion time Δ = 10 ms was used for the experiments. Ten gradient increments were acquired, ranging from 5.9–53.2 G•cm^{-1} in equal steps of gradient squared.

Microwave-assisted reactions were carried out using the CEM Focused Microwave™ Synthesis System, Discover® SP (CEM, North Carolina, USA). Flash column chromatography was carried out using Silica 60 Å (particle size 40–63 µm, Sigma Aldrich, UK) as the stationary phase. Size exclusion chromatography was carried out using Sephadex LH-20 (MeOH) and Bio-Beads SX-1 (CH_2Cl_2) as the stationary phase. TLC was performed on precoated silica gel plates (0.25 mm thick, 60 F_{254}, Merck, Germany) and visualized using both short and long wave ultraviolet light in combination with standard laboratory stains (basic potassium permanganate, acidic ammonium molybdate and ninhydrin). Low resolution ESI mass spectrometry was performed on a Thermo Scientific LCQ Fleet Ion Trap Mass Spectrometer or an Agilent Technologies 1200 LC system with either an Agilent 6130 single quadrupole MS detector or an Advion Expression LCMS single quadrupole MS detector. CD and UV/Vis

spectroscopy was measured on an Applied Photophysics Ltd Chirascan CD Spectrometer. High-resolution mass spectrometry (HRMS) and MALDI-TOF (matrix assisted laser desorption/ionization time-of-flight) was carried out by staff at the Mass Spectrometry Service, Department of Chemistry, The University of Manchester and the Mass Spectrometry Service, East China Normal University.

S3. REACTIONS SCHEMES

3.1. Synthesis of linkers

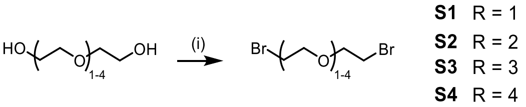

Scheme S1. Synthesis of polyethylene glycol linkers **S1-S4**. Reagents and conditions: CBr_4, PPh_3, THF, 0°C to RT, 16 h, 57% / 73% / 52% / 48% (for **S1**/**S2**/**S3**/**S4**).

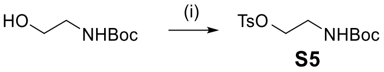

Scheme S2. Synthesis of intermediate linker **S5**. Reagents and conditions: (i) *p*-toluenesulfonyl chloride, NEt_3, CH_2Cl_2, RT, 3 h, 74%.

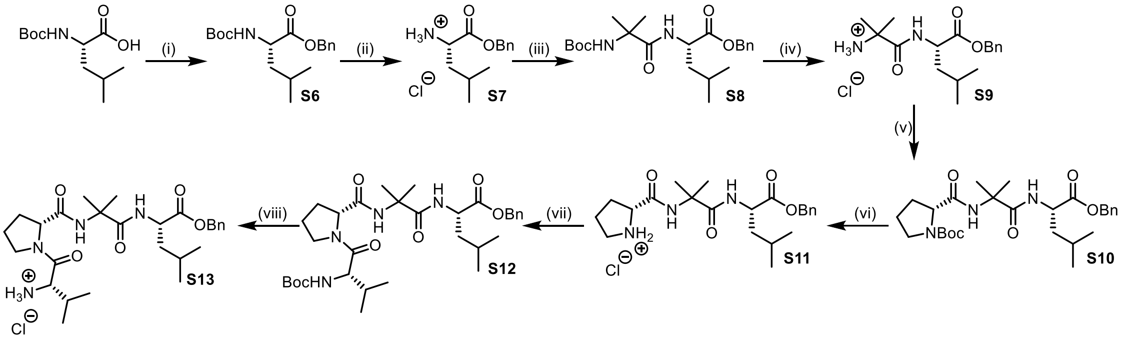

Scheme S3. Synthesis of tetrapeptide hairpin **S13**. Reagents and conditions: (i) BnBr, Cs_2CO_3, DMF, 0 °C to RT overnight, 72%; (ii) HCl (4 M), 1,4-dioxane, RT, 1 h; (iii) 2-(Boc-amino)isobutyric acid, HOBt·H_2O, EDC·HCl, DIPEA, CH_2Cl_2, RT, 4 h, 86% over two steps; (iv) HCl (4 M), 1,4-dioxane, 1 h; (v) *N*-Boc-D-proline, HATU, DIPEA, RT, 2 h, 82% over two steps; (vi) HCl (4 M), 1,4-dioxane, RT, 1 h; (vii) *N*-Boc-L-valine, HATU, DIPEA, RT, 2 h, 82% over two steps; (viii) HCl (4 M), 1,4-dioxane, RT, 1 h, quant.

3.2 Synthesis of ligands 1-7

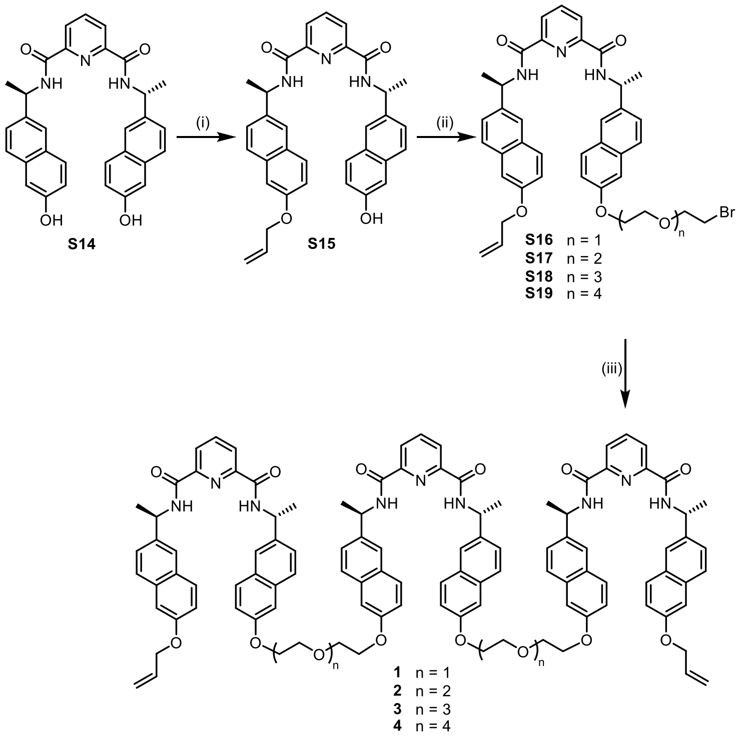

Scheme S4. Synthesis of tridentate ligands 1-4. Reagents and conditions: (i) allyl bromide, K_2CO_3, DMF, 80°C, 2 h, 43%. (ii) **S1**/**S2**/**S3**/**S4**, K_2CO_3, DMF, 80°C, 4 h (for **S16**, **S17, S18**) *or* RT, 16 h (**S19**), 42% / 73% / 49% / 72% (for **S16**/**S17**/**S18**/**S19**). (iii) **S14**, K_2CO_3, DMF, 80°C, 16-24 h, 38% / 68% / 43% / 68% (for **1**/**2**/**3**/**4**).

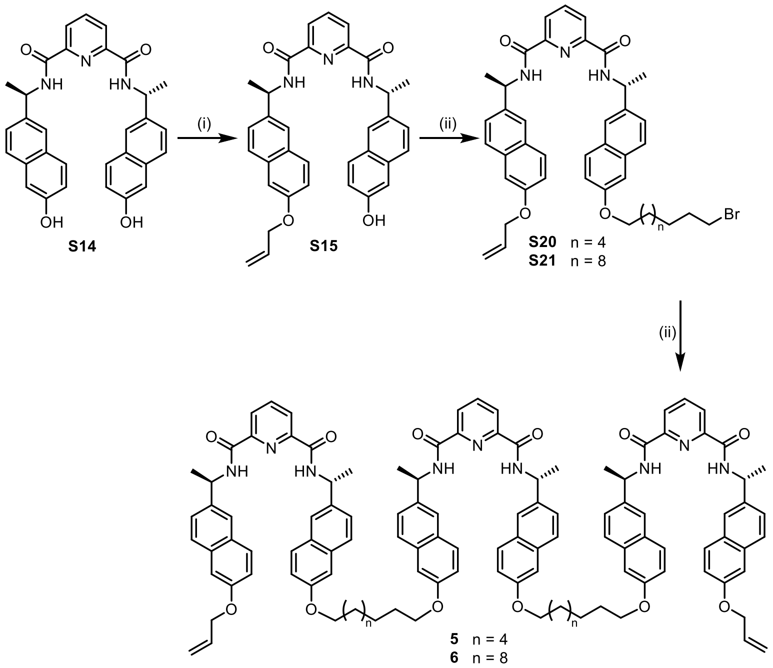

Scheme S5. Synthesis of tridentate ligands **5**-**6**. Reagents and conditions: (i) allyl bromide, K_2CO_3, DMF, 80°C, 2 h, 43%. (ii) 1,8-dibromo-octane *or* 1,12-dibromo-dodecane, K_2CO_3, DMF, RT, 72h, 72% / 72% (for **S20**/**S21**). (iii) **S14**, K_2CO_3, DMF, 80°C, 16 h, 52% / 74% (for **5**/**6**).

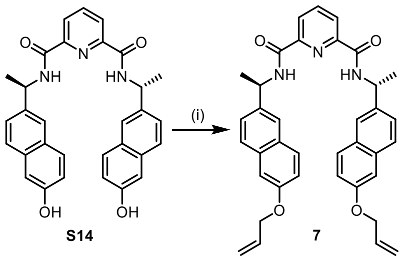

Scheme S6. Synthesis of ligand **7**. Reagents and conditions: (i) allyl bromide, K_2CO_3, DMF, RT, 16 h, 63%.

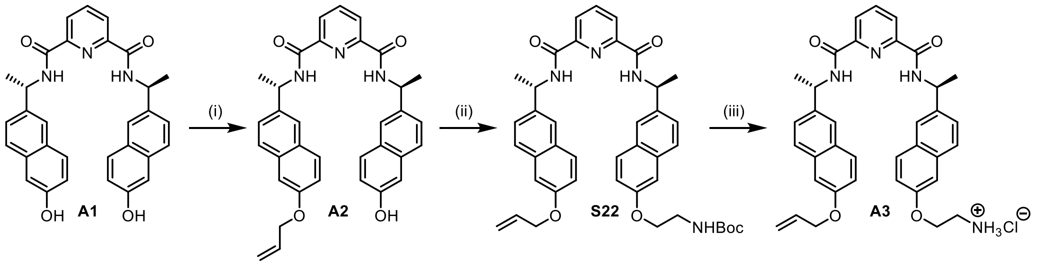

Scheme S7. Synthesis of amine-appended ligand intermediate **A3**. Reagents and conditions: (i) allyl bromide, K_2CO_3, DMF, 80°C, 2 h, 43% or allyl bromide, NaOH (s, ground), DMF, 60 ˚C, overnight, 46%. (ii) **S5**, Cs_2CO_3, DMF, 80°C, 16 h, 98%. (iii) HCl (4 M), 1,4-dioxane, 1 h, quant.

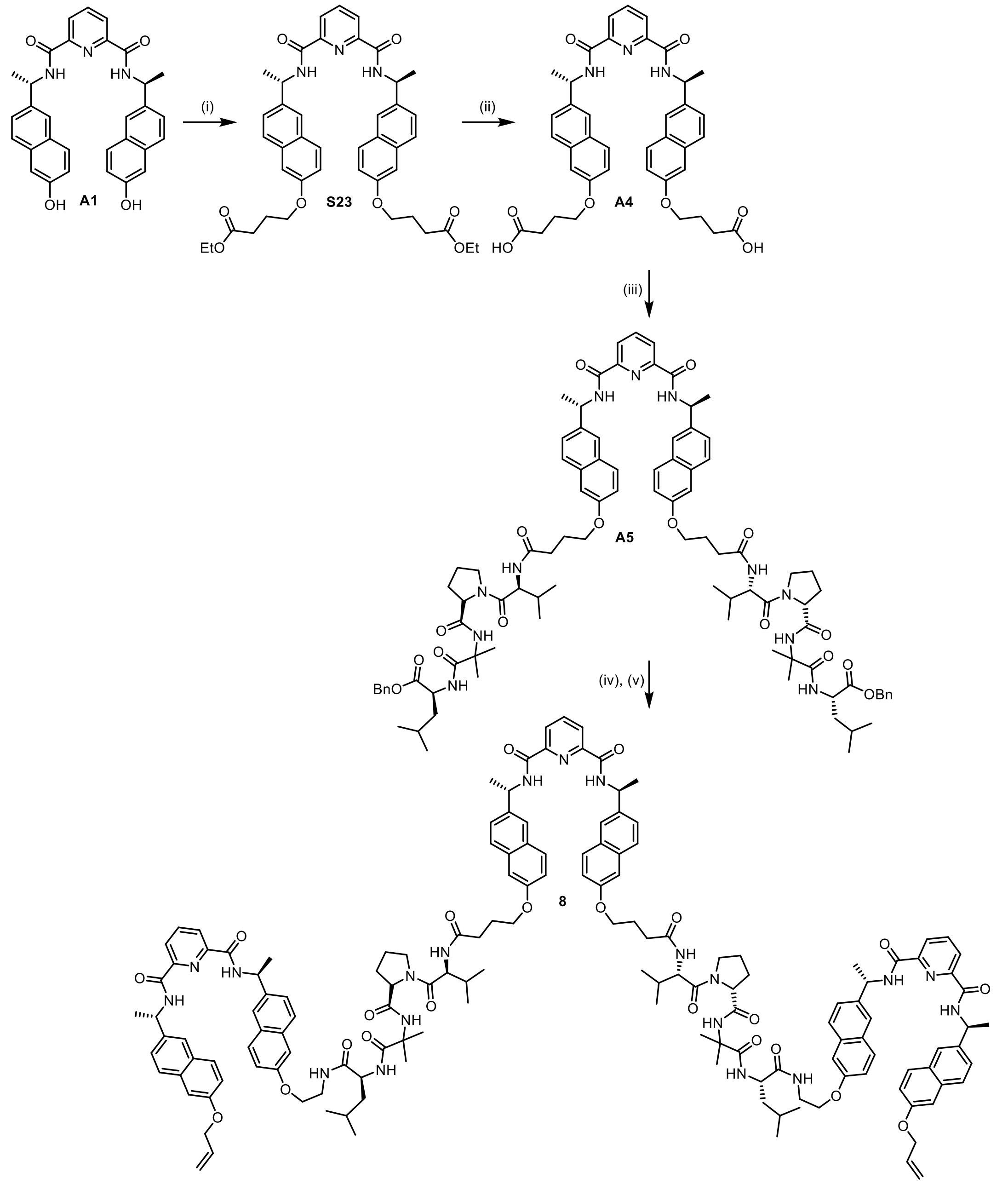

Scheme S8. Synthesis of metallopeptide ligand **8**. Reagents and conditions: (i) ethyl-4-bromobutyrate, Cs_2CO_3, DMF, RT, overnight, 81%; (ii) NaOH, EtOH/H_2O/THF (1:1:1), 40 °C, 3 h; (iii) **S13**, HATU, DIPEA, DMF, 4 h, 67%; (iv) $Pd(OH)_2$/C, MeOH, 35 °C, overnight; (v) **A3**, HATU, DIPEA, DMF, 5 h, 78%.

3.3 Synthesis of overhand knots and circular helicates

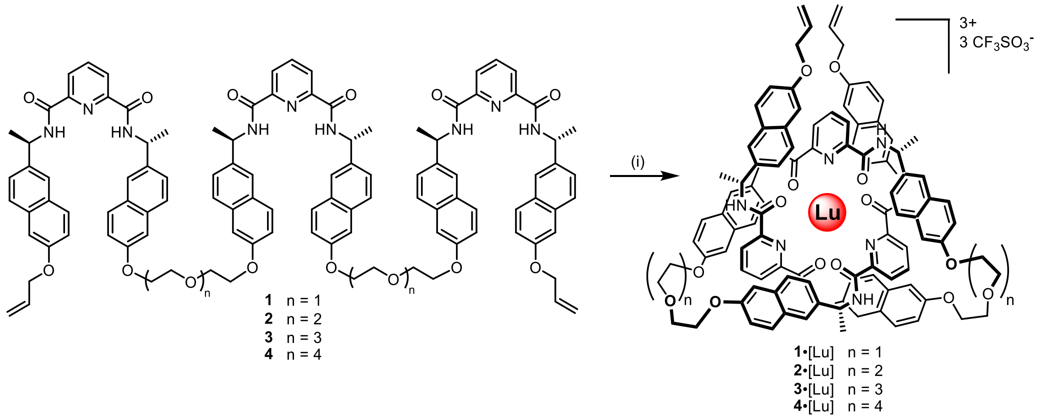

Scheme S9. Synthesis of overhand knots **1**•[Lu], **2**•[Lu],**3**•[Lu] and **4**•[Lu]. Reagents and conditions: $Lu(CF_3SO_3)_3$, MeCN, 80 °C, 16 h, 12% / 90% / 76% / 64% (for **1**•[Lu]/**2**•[Lu]/**3**•[Lu]/**4**•[Lu]).

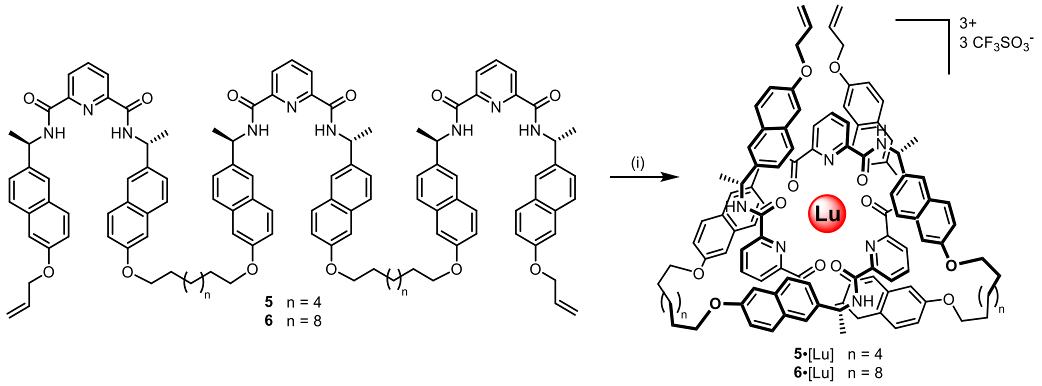

Scheme S10. Synthesis of overhand knots **5**•[Lu] and **6**•[Lu]. Reagents and conditions: $Lu(CF_3SO_3)_3$, MeCN, 80 °C, 16 h, 53% / 52% (for **5**•[Lu]/**6**•[Lu]).

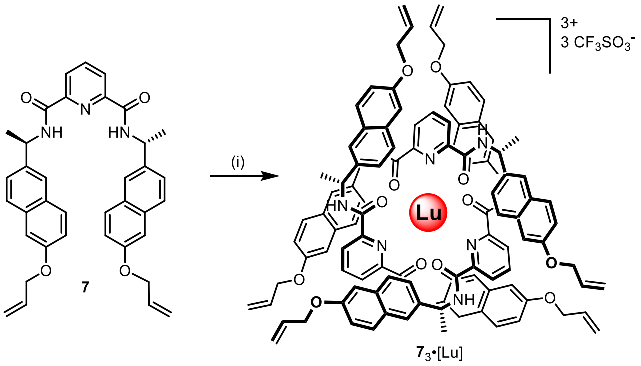

Scheme S11. Synthesis of circular helicate 7_3•[Lu]. Reagents and conditions: (i) $Lu(CF_3SO_3)_3$, MeCN, RT, 16 h, 86%.

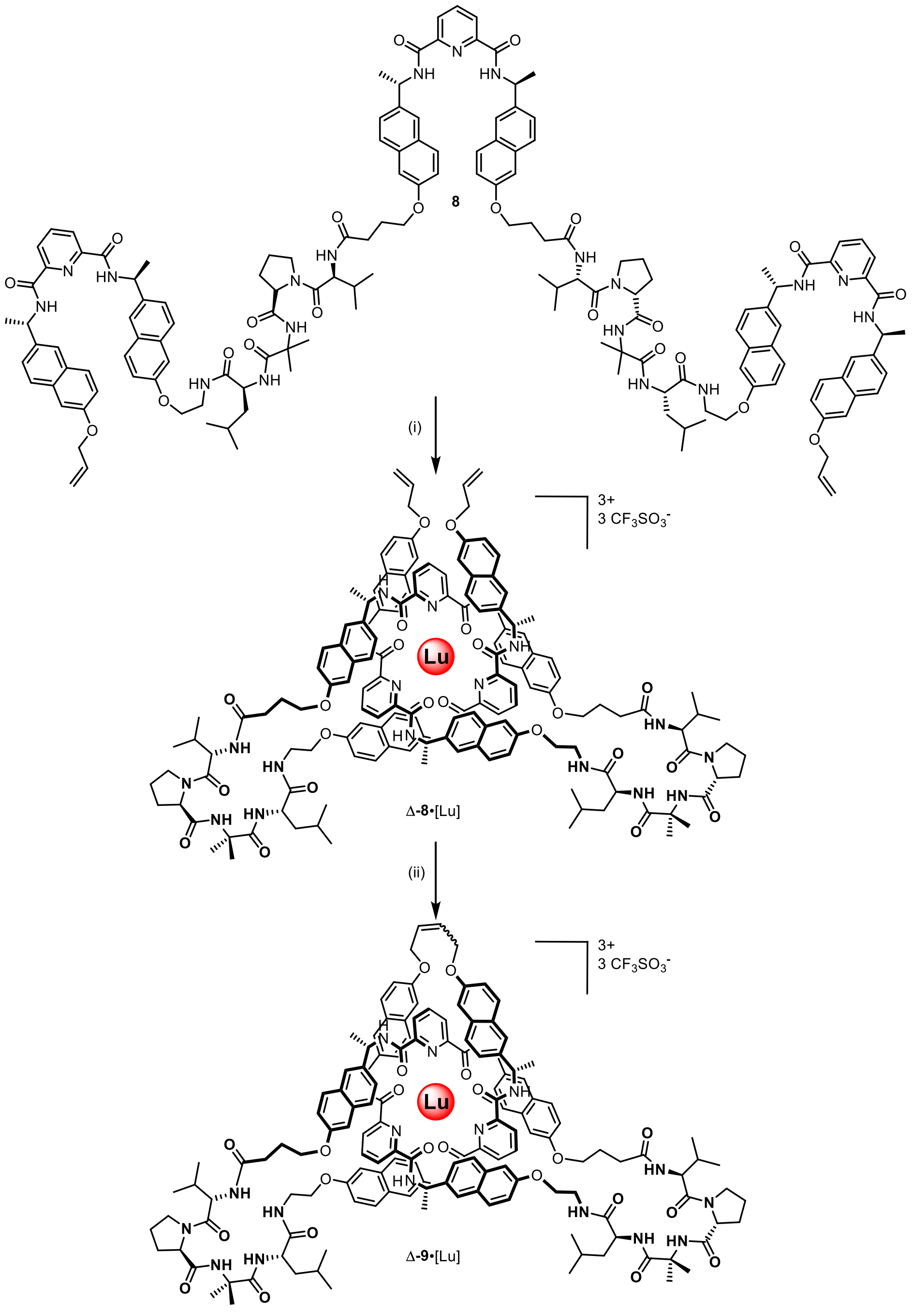

Scheme S12. Synthesis of metallopeptide overhand knot **8**•[Lu] and covalent capture conditions. Reagents and conditions: (i) $Lu(CF_3SO_3)_3$, MeCN, MW (80 W, 80 °C), 8 h, 97%. (ii) Hoveyda-Grubbs 2nd generation catalyst, CH_2Cl_2/$MeNO_2$ 3:2, 50°C, 24 h, 90%.

S4. EXPERIMENTAL PROCEDURES

S4.1. Synthetic procedures and characterization

General method A for deprotection of *tert*-butoxycarbonyl (Boc) group:

The Boc-protected starting material was dissolved in a solution of hydrochloric acid in 1,4-dioxane (4.0 M) and the resulting mixture was left stirring at room temperature for 1 h. The reaction mixture was concentrated under reduced pressure after diluting with dichloromethane and the crude product was carried through to the next step without further purification.

General method B for amino acid coupling:

A mixture of carboxylic acid and appropriate coupling reagents was stirred in CH_2Cl_2 or DMF for 10 min, after which the amine in CH_2Cl_2 was added. The resulting solution was stirred for 10 min followed by the dropwise addition of di-*iso*propylethylamine (DIPEA). The reaction mixture was left stirring at room temperature for the appropriate amount of time. The reaction mixture was diluted by CH_2Cl_2 and washed successively with citric acid (aq, 10%), sodium bicarbonate (sat. aq.) and brine. The combined organic phases were dried over sodium sulphate and concentrated under reduced pressure to give the crude product. Flash column chromatography, eluting with hexane and EtOAc in the appropriate ratio, was used to yield the pure products.

S6

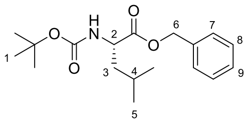

A solution of *N*-Boc-L-leucine (2.5 g, 10.03 mmol) and caesium carbonate (6.53 g, 20.06 mmol) in DMF (40 mL) was stirred at 0 °C for 1 h, after which benzyl bromide (1.78 mL, 15.04 mmol) was added. The resulting solution was left stirring at room2temperature overnight after which it was concentrated under reduced pressure to give the crude product as a yellow oil. Flash column chromatography (hexane/EtOAc 7:1) yielded the pure compound **S6** as a colourless oil (2.31 g, 72%). **^{1}H NMR** (500 MHz, $CDCl_3$) δ = 7.38 – 7.31 (m, 5H, $H_{7,8,9}$), 5.19 (d, *J* = 12.5 Hz, 1H, H_6), 5.13 (d, *J* = 12.5 Hz, 1H, H_6), 4.89 (d, *J* = 8.9 Hz, 1H, *N-H*), 4.36 (td, *J* = 8.9, 5.2 Hz, 1H, H_2), 1.72 – 1.58 (m, 2H, H_3), 1.52 – 1.48 (m, 1H, H_4), 1.43 (s, 9H, H_1), 0.93 (d, *J* = 4.1 Hz, 3H, H_5), 0.91 (d, *J* = 4.1, 3H, H_5); **^{13}C NMR** (126 MHz, $CDCl_3$) δ = 173.48, 155.54, 135.65, 128.69, 128.44, 128.30, 80.03, 67.02, 52.34, 41.87, 28.45, 24.91, 22.96, 22.10. **HRMS** (ESI+): Calcd. for $C_{18}H_{27}NO_4Na^+$ 344.1828, found 344.1832 $[M+Na]^+$.

S7

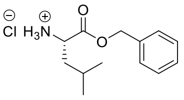

Using the general method A, the compound **S6** (2.31 g, 7.19 mmol) was converted into the title compound as a colourless solid. The crude compound was carried through to the next step without further purification.

S8

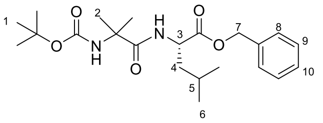

Using the general method B, the compound **S7** (1.85 g, 7.19 mmol) was converted into the title compound (2.51 g, 86%) as a colourless solid in the presence of 2-(Boc-amino)isobutyric acid (1.60 g, 7.91 mmol), HOBt·H_2O (1.16 g, 8.63 mmol), EDC·HCl (1.70 g, 8.63 mmol) and DIPEA (3.0 mL, 17.27 mmol). **^{1}H NMR** (500 MHz, $CDCl_3$) δ = 7.41-7.29 (m, 5H, $H_{8,9,10}$), 6.88 (m, 1H, *N-H*), 5.17 (d, *J* = 12.3, 1H, H_7), 5.12 (d, *J* = 12.3, 1H, H_7), 4.93 - 4.80 (m, 1H, *N-H*), 4.66-4.59 (m, 1H, H_3), 1.72 – 1.59 (m, 2H, H_4), 1.59 – 1.52 (m, 1H, H_5), 1.50 (app s, 3H, H_2), 1.45 (app s, 3H, H_2), 1.43 (app s, 9H, H_1), 0.92 (d, *J* = 6.2, 3H, H_6), 0.90 (d, *J* = 6.2, 3H, H_6); **^{13}C NMR** (126 MHz, $CDCl_3$) δ = 174.48, 173.03, 154.73, 135.60, 128.70, 128.48, 128.36, 80.35, 67.08, 56.90, 51.02, 41.69, 28.39, 24.83, 23.03, 21.95. **HRMS** (ESI+): Calcd. for $C_{22}H_{35}N_2O_5$ 407.2536, found 407.2540 $[M+H]^+$.

S9

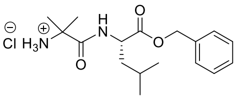

Using the general method A, the compound **S8** (2.46 g, 3.61 mmol) was converted into the title compound as a white foam. The crude compound was carried through to the next step without further purification.

S10

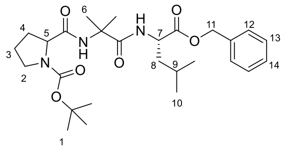

Using the general method B, the compound **S9** (2.0 g, 5.85 mmol) was converted into the title compound (2.40 g, 82%) as a colourless solid by the addition of *N*-Boc-D-proline (1.26 g, 5.85 mmol), HATU (2.22 g, 5.85 mmol) and DIPEA (2.2 mL, 12.86 mmol). **^{1}H NMR** (500 MHz, $CDCl_3$) δ = 7.40 – 7.29 (m, 6H, $H_{12,13,14,N\text{-}H}$), 6.71 (s, 1H, *N-H*), 5.20 - 5.06 (m, 2H, H_{11}), 4.63 - 4.51 (m, 1H, H_7), 4.18 - 4.12 (m, 1H, H_5), 3.48 - 3.40 (m, 2H, H_2), 2.13 - 2.07 (m, 2H, H_4), 1.88 (dt, *J* = 12.2, 6.9, 2H, H_3), 1.74 - 1.61 (m, 5H, $H_{6,8}$), 1.55 (s, 3H, H_6), 1.46 (s, 9H, H_1), 1.43 (m, 1H, H_9), 0.90 (m, 6H, H_{10}); **^{13}C NMR** (126 MHz, $CDCl_3$) δ = 174.29, 173.05, 171.54, 156.05, 135.87, 128.62, 128.28, 81.07, 66.81, 61.30, 57.28, 51.33, 47.40, 40.69, 28.47, 26.95, 24.81, 24.49, 23.11, 21.70. **HRMS** (ESI+): Calcd. for $C_{27}H_{42}N_3O_6$: 504.3068, found 504.3064 $[M+H]^+$.

S11

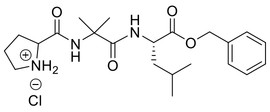

Using the general method A, the compound **S10** (2.40 g, 4.77 mmol) was converted into the title compound as a white foam. The crude compound was carried through to the next step without further purification.

S12

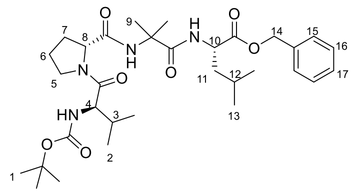

Using the general method B, the compound **S11** (2.0 g, 4.59 mmol) was converted into the title compound (2.27 g, 82%) as a colourless solid by the addition of *N*-Boc-L-valine (1.0 g, 4.59 mmol), HATU (1.75 g, 4.59 mmol) and DIPEA (1.8 mL, 10.1 mmol). **^{1}H NMR** (500 MHz, $CDCl_3$) δ = 7.36 – 7.31 (m, 5H, $H_{15,16,17}$), 7.23 - 7.19 (m, 1H, *N-H*), 6.73 (s, 1H, *N-H*), 5.41 (d, *J* = 8.5, 1H, *N-H*), 5.17 (d, *J* = 12.4, 1H, H_{14}), 5.10 (d, *J* = 12.4, 1H, H_{14}), 4.65 - 4.58 (m, 1H, H_{10}), 4.37 - 4.32 (m, 1H, H_8), 4.18 (dd, *J* = 8.6, 7.4, 1H, H_4), 3.93 - 3.86 (m, 1H, H_5), 3.62 - 3.53 (m, 1H, H_5), 2.29 -2.19 (m, 1H, H_7), 2.13 - 2.06 (m, 1H, H_7), 2.04 -1.94 (m, 3H, $H_{3,6}$), 1.70 - 1.61 (m, 2H, H_{11}), 1.58 -1.56 (m, 1H, H_{12}), 1.50 (app s, 3H, H_9), 1.42 (app s, 3H, H_9), 1.42 (s, 9H, H_1), 0.97 (d, *J* = 6.7, 6H, $H_{2/13}$), 0.90 (d, *J* = 6.2, 6H, $H_{2/13}$); **^{13}C NMR** (126 MHz, $CDCl_3$) δ = 174.12, 173.32, 172.57, 170.66, 156.14, 135.87, 128.63, 128.29, 79.92, 66.86, 61.19, 57.77, 57.43, 50.99, 47.67, 41.27, 30.75, 28.53, 28.42, 26.00, 25.52, 25.03, 24.89, 23.14, 21.88, 19.78, 18.02. **HRMS** (ESI+): Calcd. for $C_{32}H_{51}N_4O_7$: 603.3713, found 603.3748 $[M+H]^+$.

S13

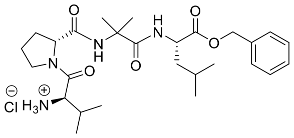

Using the general method A, the compound **S12** (1.01 g, 4.88 mmol) was converted into the title compound as a white foam. The crude compound was carried through to the next step without further purification.

S16

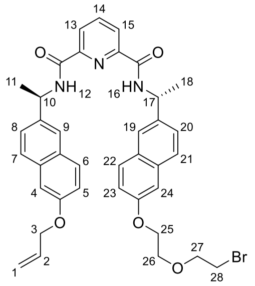

To a solution of **S15** (39 mg, 71.9 µmol) and potassium carbonate (40 mg, 288 µmol) in degassed DMF (2.5 mL) was added **S1** (66 mg, 288 µmol). The solution was heated to 80 °C for 4 h after which it was concentrated under reduced pressure. Flash column chromatography (CH_2Cl_2 to CH_2Cl_2/EtOAc, 3:1) yielded the pure compound **S16** as a colourless solid (21 mg, 42 %). **^{1}H NMR** (600 MHz, $CDCl_3$) δ 8.38 (d, *J* = 7.8 Hz, 2H, $H_{13,15}$), 8.05 (t, *J* = 7.8 Hz, 1H, H_{14}), 7.89 (d, *J* = 8.1 Hz, 2H, $H_{12,16}$), 7.75 (s, 2H, $H_{9,19}$), 7.70 (d, *J* = 9.0 Hz, 2H, $H_{6,22}$), 7.66 (m, 2H, $H_{7,21}$), 7.45 (m, 2H, $H_{8,20}$), 7.21 (dd, *J* = 8.9, 2.5 Hz, 2H, $H_{5,23}$), 7.16 – 7.13 (m, 2H, $H_{4,24}$), 6.15 (ddt, *J* = 17.0, 10.5, 5.3 Hz, 1H, H_2), 5.53 - 5.43 (m, 3H, $H_{1(trans),10,17}$), 5.36 - 5.33 (m, 1H, $H_{1(cis)}$), 4.69 - 4.65 (m, 2H, H_3), 4.32 – 4.27 (m, 2H, H_{25}), 4.00 – 3.97 (m, 2H, H_{26}), 3.94 (t, *J* = 6.3 Hz, 2H, H_{27}), 3.54 (t, *J* = 6.3 Hz, 2H, H_{28}), 1.68 (d, *J* = 6.8 Hz, 6H, $H_{11,18}$). **^{13}C NMR** (151 MHz, $CDCl_3$) δ 162.55, 156.88, 156.77, 148.81, 139.07, 137.97, 137.89, 133.90, 133.86, 133.05, 129.40, 128.91, 128.84, 127.58, 127.55, 125.16, 124.93, 124.90, 124.58, 119.51, 119.49, 117.89, 106.90, 106.69, 71.45, 69.64, 68.86, 67.49, 49.06, 30.23, 21.67, 21.62; Due to high degree of apparent symmetry, several ^{13}C signals overlap. **HRMS** (ESI+): Calcd. for $C_{38}H_{38}O_5BrN_3Na$: 718.1887, found 718.1869 $[M+Na]^+$.

S19

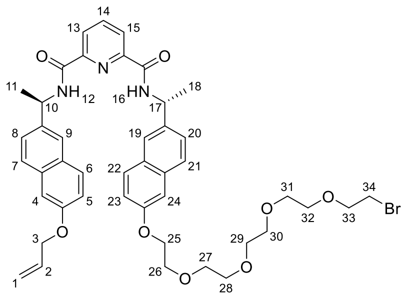

To a solution of **S15** (33 mg, 0.06 mmol) and potassium carbonate (34 mg, 0.24 mmol) in degassed DMF (2.0 mL) was added **S4** (88 mg, 0.24 mmol). The solution was stirred at room temperature for 16 h after which it was concentrated under reduced pressure. Flash column chromatography (CH_2Cl_2 to EtOAc) yielded the pure compound **S19** as a colourless solid (36 mg, 72%) . **^{1}H NMR** (600 MHz, $CDCl_3$) δ 8.39 (d, *J* = 7.8 Hz, 2H, $H_{13,15}$), 8.05 (t, *J* = 7.8 Hz, 1H, H_{14}), 7.88 (d, *J* = 8.1 Hz, 2H, $H_{12,16}$), 7.77 - 7.74 (m, 2H, $H_{9,19}$), 7.72 - 7.65 (m, 4H, $H_{6,7,21,22}$), 7.48 – 7.44 (m, 2H, $H_{8,20}$), 7.23 - 7.20 (m, 2H, $H_{5,23}$), 7.16 - 7.13 (m, 2H, $H_{4,24}$), 6.15 (ddt, *J* = 17.2, 10.6, 5.3 Hz, 1H, H_2), 5.53 - 5.43 (m, 3H, $H_{1(trans),10,17}$), 5.37 - 5.34 (m, 1H, $H_{1(cis)}$), 4.70 - 4.65 (m, 2H, H_3), 4.28 (t, *J* = 4.9 Hz, 2H, H_{25}), 3.98 – 3.94 (m, 2H, H_{26}), 3.81 (t, *J* = 6.4 Hz, 2H, H_{33}), 3.80 – 3.78 (m, 2H, H_{27}), 3.74 – 3.72 (m, 2H, H_{32}), 3.70 – 3.68 (m, 4H, $H_{28,31}$), 3.68 (br s, 4H, $H_{29,30}$), 3.47 (t, *J* = 6.3 Hz, 2H, H_{34}), 1.71 - 1.67 (m, 6H, $H_{11,18}$); **^{13}C NMR** (151 MHz, $CDCl_3$) δ 162.54, 157.02, 156.76, 148.80, 139.09, 137.89, 137.87, 133.89, 133.04, 129.41, 129.35, 128.83, 127.56, 125.17, 124.92, 124.87, 124.59, 119.58, 119.52, 117.91, 106.86, 106.55, 71.19, 70.91, 70.67, 70.60, 70.54,

69.73, 68.85, 67.48, 49.06, 30.35, 21.67, 21.63; Due to high degree of apparent symmetry, several ^{13}C signals overlap; **HRMS** (ESI+): Calcd. for $C_{44}H_{50}O_8BrN_3Na$: 850.2673, found 850.2662 $[M+Na]^+$.

S21

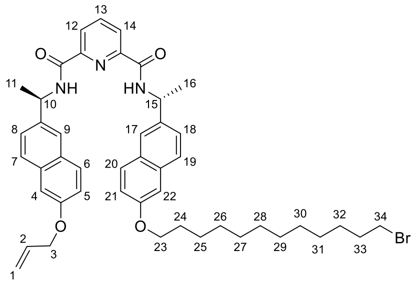

To a solution of **S15** (136 mg, 0.25 mmol) and potassium carbonate (103 mg, 0.75 mmol) in degassed DMF (25 mL) was added 1,12-dibromododecane (245 mg, 0.75 mmol). The reaction was stirred for 3 days at room temperature, after which the mixture was concentrated under reduced pressure. Flash column chromatography (CH_2Cl_2 to CH_2Cl_2/EtOAc 3:1) yielded the pure compound **S21** as a colourless solid (318 mg, 72 %). **^{1}H NMR** (600 MHz, $CDCl_3$) δ 8.38 (t, *J* = 7.4 Hz, 2H, $H_{12,14}$), 8.05 (t, *J* = 7.7 Hz, 1H, H_{13}), 7.88 (d, *J* = 8.0 Hz, 2H, *N-H*), 7.72 (s, 2H, $H_{9,17}$), 7.70 (d, *J* = 9.0 Hz, 1H, $H_{6/20}$), 7.66 (d, *J* = 8.9 Hz, 1H, $H_{6/20}$), 7.65 (m,1H, $H_{7,19}$), 7.44 (m, 2H, $H_{8,18}$), 7.21 (dd, *J* = 2.5, 8.9 Hz, 1H, $H_{5/21}$), 7.16 (dd, *J* = 2.5, 8.9 Hz, 1H, $H_{5/21}$), 7.14 (m, 1H, $H_{4/22}$), 7.12 (m, 1H, $H_{4/22}$), 6.18 – 6.10 (m, 1H, H_2), 5.49 (m, 1H, H_{1trans}), 5.43 (q, *J* = 7.3 Hz, 2H, $H_{10,15}$), 5.34 (m, 1H, H_{1cis}), 4.66 (d, *J* = 5.3 Hz, 2H, H_3), 4.08 (t, *J* = 6.5 Hz, 2H, H_{23}), 3.42 (t, *J* = 6.8 Hz, 2H, H_{34}), 1.90 – 1.84 (m, 4H, $H_{24,33}$), 1.64 (d, *J* = 6.8 Hz, 6H, $H_{11,16}$), 1.56 – 1.49 (m, 2H, $H_{25,32}$), 1.48 - 1.30 (m, 12H, $H_{26,27,28,29,30,31}$); **^{13}C NMR** (151 MHz, $CDCl_3$) δ 162.89, 157.73, 157.12, 149.18, 149.16, 139.42, 138.24, 137.98, 134.37, 134.25, 133.39, 129.74, 129.62, 129.18, 129.03, 127.92, 127.83, 125.49, 125.23, 125.16, 124.93, 119.93, 119.86, 118.23, 107.23, 106.78, 69.21, 68.44, 49.43, 34.43, 33.18, 29.91, 29.89, 29.87, 29.78, 29.77, 29.60, 29.12, 28.52, 26.46, 22.00, 21.97. Due to high degree of apparent symmetry, several ^{13}C signals overlap. **HRMS** (ESI^-): Calcd. for $C_{46}H_{55}N_3O_4Br$: 792.3370, found 792.3384 $[M+H^-]^-$.

S22

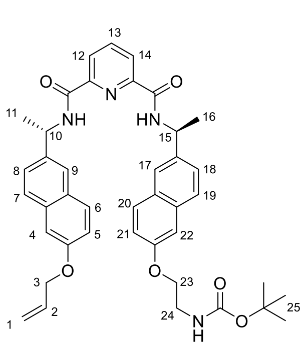

To a stirred mixture of **A2** (190 mg, 0.35 mmol) and caesium carbonate (568 mg, 1.74 mmol) in degassed DMF (3 mL) was added **S5** (549 mg, 1.74 mmol) in degassed DMF (3 mL). The reaction mixture was stirred at 80 °C for 16 h, cooled to room temperature and diluted with EtOAc (15 mL). The resulting mixture was washed with lithium chloride (aq, 5%, 10 mL) and extracted with EtOAc (3 ×10 mL). The combined organics were dried over sodium sulphate and concentrated under reduced pressure to give the crude compound as a yellow oil. Flash column chromatography (CH_2Cl_2/EtOAc 5:1) yielded the pure compound **S22** as a colourless

solid (236 mg, 98%). **^{1}H NMR** (500 MHz, $CDCl_3$) δ = 8.36 (d, *J* = 7.8, 2H, $H_{12,14}$), 8.03 (t, *J* = 7.8, 1H, H_{13}), 7.87 (d, *J* = 2.6, 1H, *N-H*), 7.85 (d, *J* = 2.6, 1H, *N-H*), 7.73 (m, 2H, $H_{9,17}$), 7.71 – 7.61 (m, 4H, $H_{6,7,19,20}$), 7.49 – 7.38 (m, 2H, $H_{8,18}$), 7.19 (dd, *J* = 8.9, 2.5, 1H, $H_{5/21}$), 7.15 (dd, *J* = 8.9, 2.5 Hz, 1H, $H_{5/21}$), 7.13 (d, *J* = 2.5, 1H, $H_{4/22}$), 7.09 (d, *J* = 2.5, 1H, $H_{4/22}$), 6.13 (m, 1H, H_2), 5.53 – 5.40 (m, 3H, $H_{1trans,10,15}$), 5.36 - 5.32 (m, 1H, H_{1cis}), 4.68 - 4.65 (m, 2H, H_3), 4.15 (t, *J* = 5.4, 2H, H_{23}), 3.64 - 3.58 (m, 2H, H_{24}), 1.67 (d, *J* = 6.9, 3H, $H_{11/16}$), 1.66 (d, *J* = 6.9, 3H, $H_{11/16}$), 1.46 (app s, 9H, H_{25}); **^{13}C NMR** (126 MHz, $CDCl_3$) δ = 162,70, 156.93, 148.96, 139.24, 138.18, 138.04, 134.06, 133.19, 133.19, 129.61, 129.55, 129.00, 127.74, 127.71, 125.32, 125.08, 124.75, 124.72, 119.66, 119.35, 118.05, 107.06, 106.76, 79.75, 69.02, 67.42, 49.14, 40.66, 28.56, 21.76. Due to high degree of apparent symmetry, several ^{13}C signals overlap. **HRMS** (ESI+): Calcd. for $C_{41}H_{44}N_4O_6Na^+$: 711.3153, found 711.3146 $[M+Na]^+$.

A3

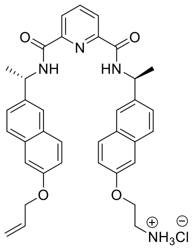

Using the general method A, **S22** (50 mg, 0.073 mmol) was converted into the title compound as an off-white solid. The crude compound was carried through to the next step without further purification

S23

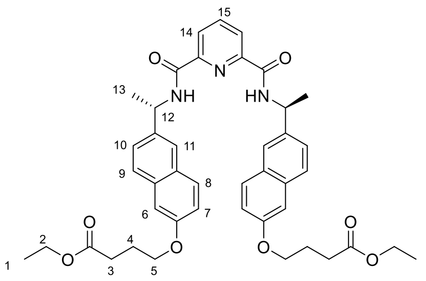

To a stirred solution of compound **A1** (311 mg, 0.62 mmol) and caesium carbonate (0.8 g, 2.46 mmol) in degassed DMF (8 mL) was added ethyl-4-bromobutyrate (0.2 mL, 1.35 mmol). The reaction mixture was stirred at room temperature overnight, after which it was diluted with EtOAc (15 mL). The resulting solution was washed with lithium chloride (aq, 5%, 10 mL) and extracted with EtOAc (3 ×15 mL). The combined organics were dried over sodium sulphate and concentrated under reduced pressure to give the crude compound as a yellow oil. Flash column chromatography (CH_2Cl_2/EtOAc 5:1) yielded the pure compound **S23** as a white solid (366 mg, 81%). **^{1}H NMR** (500 MHz, $CDCl_3$) δ = 8.35 (d, *J* = 7.8, 2H, H_{14}), 8.02 (t, *J* = 7.8, 1H, H_{15}), 7.91 – 7.86 (m, 2H, *N-H*), 7.72 (d, *J* = 1.7, 2H, H_{11}), 7.67 (d, *J* = 9.0, 2H, H_8), 7.65 (d, *J* = 9.0, 2H, H_9), 7.43 (dd, *J* = 8.5, 1.8, 2H, H_{10}), 7.14 (dd, *J* = 8.9, 2.5 Hz, 2H, H_7), 7.10 (d, *J* = 2.5, 2H, H_6), 5.44 (quintet, *J* = 7.0 Hz, 2H, H_{12}), 4.20 – 4.11 (m, 8H, $H_{2,5}$), 2.56 (t, *J* = 7.3, 4H, H_3), 2.18 (quintet, *J* = 7.3, 4H, H_4), 1.67 – 1.62 (m, 6H, H_{13}), 1.26 (t, *J* = 7.1, 6H, H_1); **^{13}C NMR** (126 MHz, $CDCl_3$) δ = 173.35, 162.70, 157.19, 148.96, 139.20, 138.98, 134.08, 129.48, 128.92, 127.66, 125.03, 124.71, 119.55,

106.65, 66.96, 60.63, 49.17, 31.01, 24.77, 21.78, 14.39. **HRMS** (ESI+): Calcd. for $C_{43}H_{47}N_3O_8Na^+$: 756.3255, found 756.3249 $[M+Na]^+$.

A4

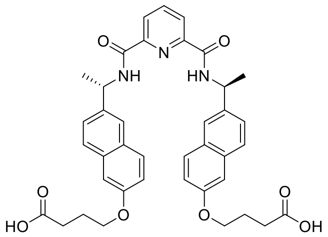

A mixture of **S23** (366 mg, 0.50 mmol) and sodium hydroxide (80 mg, 2.0 mmol) was stirred in THF (6 mL), EtOH (6 mL) and H_2O (6 mL) at 40 °C for 3 h. The reaction mixture was concentrated under reduced pressure and the resulting solid was dissolved in H_2O (25 mL). The solution was washed with hexane (30 mL) and acidified with hydrochloric acid (aq, 2.0 M, 25 mL). The aqueous phase was extracted with ethyl acetate (3×40 mL). The combined organic layers were dried over sodium sulphate and concentrated under reduced pressure to give the title compound as a colourless solid, which was carried through crude to the next step without further purification. **HRMS** (ESI+): Calcd. for $C_{39}H_{39}N_3O_8Na^+$: 700.2629, found 700.2623 $[M+Na]^+$.

A5

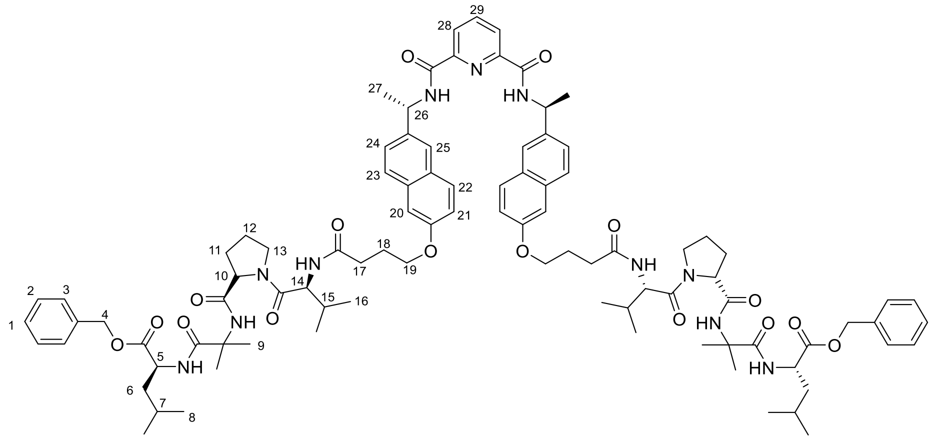

To a stirred solution of **A4** (100 mg, 0.148 mmol) and HATU (128.7 mg, 0.302 mmol) in DMF (2 mL) was added **S13** (178 mg, 0.330 mmol) in DMF (2 mL) and DIPEA (0.1 mL, 0.518 mmol). The reaction mixture was stirred at room temperature for 4 hours, after which the reaction mixture was diluted with EtOAc (10 mL). The solution was washed with lithium chloride (aq., 5%, 5 mL) and extracted with EtOAc (3 ×10 mL). The combined organics were washed with lithium chloride (aq., 5%, 5 mL), dried over sodium sulphate and concentrated under reduced pressure to give the crude product as a yellow oil. Flash column chromatography (CH_2Cl_2/EtOAc 4:1) yielded the pure compound **A5** as a colourless solid (161 mg, 67%). **^{1}H NMR** (500 MHz, $CDCl_3$) δ = 8.36 (d, *J* = 7.8, 2H, H_{28}), 8.03 (t, *J* = 7.8, 1H, H_{29}), 7.87 (dd, *J* = 7.9, 2H, *N-H*), 7.72 (d, *J* = 1.7, 2H, H_{25}), 7.65 (d, *J* = 8.6, 4H, $H_{22,23}$), 7.44 (dd, *J* = 8.6, 1.5, 2H,

H_{24}), 7.31 – 7.23 (m, 12H, $H_{1,2,3,\textit{N-H}}$), 7.13 - 7.08 (m, 4H, $H_{20,21}$), 6.56 - 6.52 (m, 2H, *N-H*), 6.48 (s, 2H, *N-H*), 5.44 (quintet, *J* = 7.1, 2H, H_{26}), 5.10 (d, *J* = 12.5, 2H, H_4), 5.03 (d, *J* = 12.5, 2H, H_4), 4.65 - 4.59 (m, 2H, H_5), 4.32 - 4.24 (m, 4H, $H_{10,14}$), 4.11 - 4.01 (m, 6H, $H_{13,19}$), 3.65 - 3.56 (m, 2H, H_{13}), 2.50 (t, *J* = 7.4, 4H, H_{17}), 2.19 – 1.93 (m, 14H, $H_{11,12,15,18}$), 1.77 – 1.69 (m, 4H, H_6), 1.67 - 1.63 (m, 6H, H_{27}), 1.61-1.57 (m, 2H, H_7), 1.55 (s, 6H, H_9), 1.44 (s, 6H, H_9), 0.97 (d, *J* = 6.7, 6 H, H_{16}), 0.95 (d, *J* = 6.7, 6H, H_{16}), 0.91-0.87 (m, 12H, H_8); **^{13}C NMR** (126 MHz, $CDCl_3$) δ = 174.53, 173.91, 173.87, 172.08, 170.84, 162.70, 157.15, 148.97, 139.23, 138.05, 134.09, 129.51, 128.95, 128.60, 128.24, 127.93, 127.67, 125.35, 125.01, 124.71, 119.46, 106.74, 67.14, 66.71, 61.77, 57.47, 51.04, 49.16, 47.93, 40.97, 32.53, 30.32, 29.27, 29.17, 26.78, 25.20, 25.08, 24.97, 24.78, 23.20, 21.87, 21.69, 19.34, 18.91. Due to the apparent symmetry, two ^{13}C signals overlap. **HRMS** (ESI+): Calcd. for $C_{93}H_{120}N_{11}O_{16}$: 1646.8870, found 1646.8884 $[M+H]^+$.

1

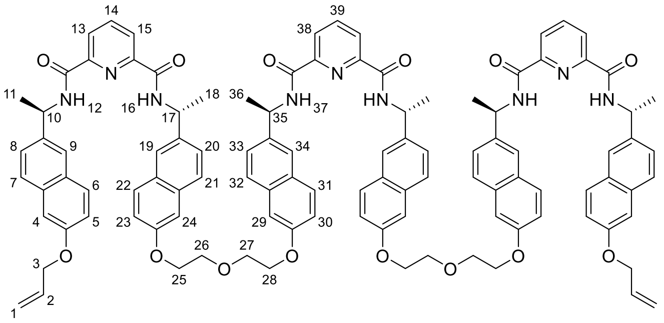

To a solution of **S16** (21 mg, 0.03 mmol) and potassium carbonate (4.2 mg, 0.03 mmol) in degassed DMF (3.0 mL) was added **S14** (7.6 mg, 0.015 mmol). The solution was stirred at 80 °C for 24 h after which the mixture was concentrated under reduced pressure. Flash column chromatography (CH_2Cl_2/EtOAc, 2:1 to CH_2Cl_2/CH_3OH, 20:1) followed by size exclusion chromatography (Bio-Beads SX-1, CH_2Cl_2) yielded the pure compound **1** as a colourless solid (10 mg, 38%). **^{1}H NMR** (600 MHz, $CDCl_3$) δ 8.37 – 8.30 (m, 6H, $H_{13,15,38}$), 8.02 – 7.94 (m, 9H, $H_{12,14,16,37,39}$), 7.69 – 7.66 (m, 6H, $H_{9,19,34}$), 7.66 – 7.61 (m, 12H, $H_{6,7,21,22,31,32}$), 7.44 - 7.39 (m, 6H, $H_{8,20,33}$), 7.20 – 7.14 (m, 6H, $H_{5,23,30}$), 7.13 – 7.10 (m, 6H, $H_{4,24,29}$), 6.14 (ddt, *J* = 17.2, 10.6, 5.3 Hz, 2H, H_2), 5.52 - 5.32 (m, 10H, $H_{1(trans),1(cis),10,17,35}$), 4.68 - 4.65 (m, 4H, H_3), 4.33 - 4.28 (m, 8H, $H_{25,28}$), 4.07 – 4.01 (m, 8H, $H_{26,27}$), 1.63 (d, *J* = 6.8 Hz, 6H, $H_{11/18/36}$), 1.57 (d, *J* = 6.8 Hz, 6H, $H_{11/18/36}$), 1.54 (d, *J* = 6.8 Hz, 6H, $H_{11/18/36}$); **^{13}C NMR** (151 MHz, $CDCl_3$) δ 162.64, 162.63, 156.93, 156.92, 156.71, 148.76, 148.73, 138.96, 138.93, 137.95, 137.94, 137.93, 133.81, 133.05, 129.33, 129.30, 128.82, 128.76, 127.50, 127.46, 125.12, 125.02, 124.49, 124.47, 119.51, 119.49, 119.47, 117.88, 106.85, 106.68, 106.66, 70.01, 68.84, 67.53, 48.93, 48.90, 48.88, 21.48, 21.43, 21.40; Due to high degree of apparent symmetry, several ^{13}C

signals overlap; **HRMS** (ESI+): Calcd. for $C_{107}H_{101}O_{14}N_9Na$: 1758.7360, found 1758.7388 $[M+Na]^+$.

3

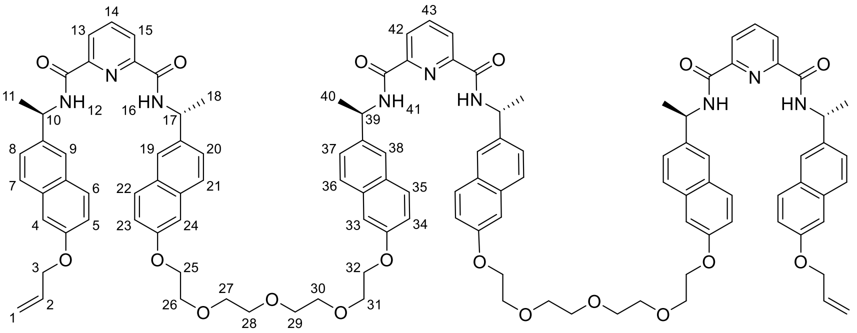

To a solution of **S18** (50 mg, 0.064 mmol) and potassium carbonate (8.8 mg, 0.064 mmol) in degassed DMF (3.2 mL) was added **S14** (61 mg, 0.032 mmol). The solution was stirred at 80 °C for 16 h after which the mixture was concentrated under reduced pressure. Flash column chromatography (EtOAc to CH_2Cl_2/CH_3OH, 20:1) followed by size exclusion chromatography (Bio-Beads SX-1, CH_2Cl_2) yielded the pure compound **3** as a colourless solid (26 mg, 43%). **^{1}H NMR** (600 MHz, $CDCl_3$) δ 8.33 (d, *J* = 7.7 Hz, 2H, $H_{13/15/42}$), 8.33 (d, *J* = 7.7 Hz, 2H, $H_{13/15/42}$), 8.29 (d, *J* = 7.8 Hz, 2H, $H_{13/15/42}$), 8.05 – 7.99 (m, 6H, $H_{12,16,41}$), 7.97 (t, *J* = 7.7 Hz, 2H, H_{14}), 7.93 (t, *J* = 7.8 Hz, 1H, H_{43}), 7.69 – 7.59 (m, 18H, $H_{6,7,9,19,21,22,35,36,38}$), 7.43 – 7.37 (m, 6H, $H_{8,20,37}$), 7.20 – 7.14 (m, 6H, $H_{5,23,34}$), 7.11 – 7.07 (m, 6H, $H_{4,24,33}$), 6.14 (ddt, *J* = 17.3, 10.5, 5.3 Hz, 2H, H_2), 5.51 - 5.47 (m, 2H, $H_{1(trans)}$), 5.46 – 5.32 (m, 8H, $H_{1(cis),10,17,39}$), 4.68 - 4.64 (m, 4H, H_3), 4.23 (t, *J* = 4.8 Hz, 8H, $H_{25,32}$), 3.92 (t, *J* = 4.8 Hz, 8H, $H_{26,31}$), 3.78 – 3.75 (m, 8H, $H_{27,30}$), 3.74 – 3.71 (m, 8H, $H_{28,29}$), 1.62 (d, *J* = 6.9 Hz, 6H, H_{11}), 1.56 – 1.52 (m, 12H, $H_{18,40}$); **^{13}C NMR** (151 MHz, $CDCl_3$) δ 162.70, 162.69, 156.95, 156.93, 156.69, 148.74, 148.71, 148.68, 138.86, 138.81, 137.96, 133.79, 133.76, 133.07, 129.30, 129.27, 129.24, 128.76, 128.72, 127.44, 127.42, 127.41, 125.08, 125.06, 125.03, 125.01, 124.44, 124.42, 119.48, 119.44, 117.86, 106.82, 106.56, 70.87, 70.71, 69.71, 68.83, 67.45, 48.84, 48.79, 21.49, 21.40, 21.38; Due to high degree of apparent symmetry, several ^{13}C signals overlap**;** **HRMS** (ESI+): Calcd. for $C_{115}H_{117}O_{18}N_9Na$: 1934.8409, found 1934.8364 $[M+Na]^+$.

4

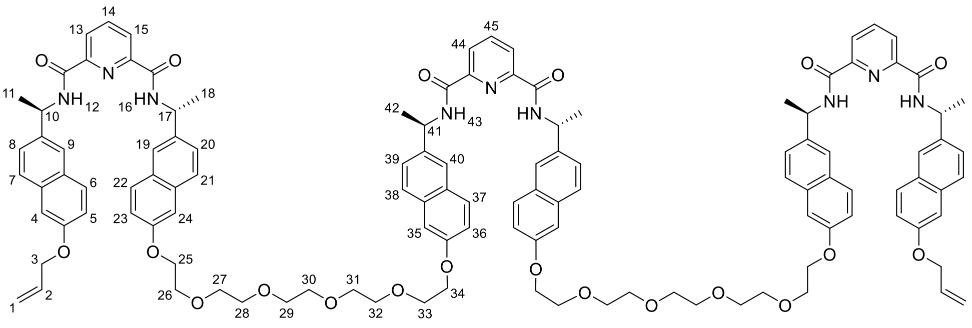

To a solution of **S19** (34 mg, 0.04 mmol) and potassium carbonate (5.7 mg, 0.04 mmol) in degassed DMF (4.0 mL) was added **S14** (10.4 mg, 0.02 mmol). The solution was stirred at 80 °C for 16 h after which the mixture was concentrated under reduced pressure. Flash column chromatography (EtOAc to CH_2Cl_2/CH_3OH, 20:1) followed by size exclusion chromatography (Bio-Beads SX-1, CH_2Cl_2) yielded the pure compound **4** as a colourless solid (28 mg, 68%). **^{1}H NMR** (600 MHz, $CDCl_3$) δ 8.34 - 8.31 (m, 4H, $H_{13/15/44}$), 8.29 (d, *J* = 7.8 Hz, 2H, $H_{13/15/44}$), 8.06 – 7.99 (m, 6H, $H_{12,16,43}$), 7.98 (t, *J* = 7.8 Hz, 2H, H_{14}), 7.93 (t, *J* = 7.8 Hz, 1H, H_{45}), 7.69 – 7.59 (m, 18H, $H_{6,7,9,19,21,22,37,38,40}$), 7.44 – 7.39 (m, 6H, $H_{8,20,39}$), 7.21 – 7.14 (m, 6H, $H_{5,23,36}$), 7.12 – 7.07 (m, 6H, $H_{4,24,35}$), 6.14 (ddt, *J* = 17.1, 10.6, 5.3 Hz, 2H, H_2), 5.51 - 5.47 (m, 2H, $H_{1(trans)}$), 5.46 – 5.37 (m, 6H, $H_{10,17,41}$), 5.36 – 5.32 (m, 2H, $H_{1(cis)}$), 4.68 - 4.64 (m, 4H, H_3), 4.23 (t, *J* = 4.3 Hz, 8H, $H_{25,34}$), 3.92 (t, *J* = 4.8 Hz, 8H, $H_{26,33}$), 3.77 – 3.73 (m, 8H, $H_{27,32}$), 3.71 – 3.69 (m, 8H, $H_{28,31}$), 3.68 (br s, 8H, $H_{29,30}$), 1.61 (d, *J* = 6.8 Hz, 6H, H_{11}), 1.57 – 1.53 (m, 12H, $H_{18,42}$); **^{13}C NMR** (151 MHz, $CDCl_3$) δ 162.63, 162.61, 156.97, 156.95, 156.71, 148.76, 148.70, 138.95, 138.88, 137.93, 137.91, 133.83, 133.80, 133.06, 129.33, 129.29, 129.26, 128.79, 128.76, 127.48, 127.47, 127.44, 125.11, 125.07, 124.99, 124.95, 124.48, 124.45, 119.51, 119.49, 119.46, 117.86, 106.84, 106.55, 70.89, 70.66, 69.70, 68.83, 67.46, 48.92, 48.88, 48.84, 21.57, 21.48, 21.45; Due to high degree of apparent symmetry, several ^{13}C signals overlap; **HRMS** (ESI+): Calcd. for $C_{119}H_{125}O_{20}N_9Na_2$: 1022.9413, found 1022.9395 $[M+2Na]^+$.

6

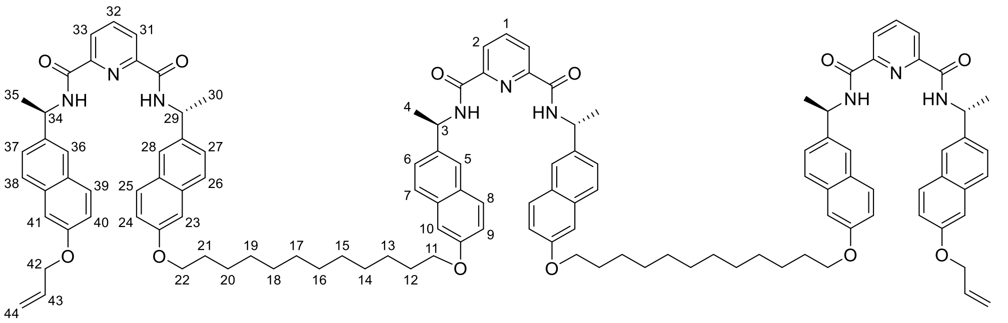

To a solution of **S21** (69 mg, 0.087 mmol) and potassium carbonate (60 mg, 0.43 mmol) in degassed DMF (8.7 mL) was added **S14** (22 mg, 0.043 mmol). The reaction was stirred for 16 hours at 80 °C. The mixture was allowed to cool to room temperature and concentrated under reduced pressure. Flash column chromatography (EtOAc to CH_2Cl_2/MeOH 9:1) yielded the pure compound **6** as a colourless solid (47 mg, 74 %). **^{1}H NMR** (600 MHz, $CDCl_3$) δ 8.36 – 8.32 (m, 6H, $H_{2,31,33}$), 8.02 – 7.94 (m, 9H, *N-H*, $H_{1,32}$), 7.72 – 7.67 (m, 6H, $H_{5,28,36}$), 7.65 – 7.60 (m, 12H, $H_{7,8,25,26,38,39}$), 7.44 – 7.39 (m, 6H, $H_{6,27,37}$), 7.21 – 7.14 (m, 6H, $H_{9,24,40}$), 7.12 – 7.08 (m, 6H, $H_{10,23,41}$), 6.19 –6.10 (m, 2H, H_{43}), 5.49 (m, 2H, $H_{44trans}$), 5.47 – 5.39 (m, 6H, $H_{3,29,34}$), 5.35 (m, 2H, H_{44cis}), 4.67 (d, *J* = 5.3 Hz, 4H, H_{42}), 4.10 – 4.06 (m, 8H, $H_{11,22}$), 1.89 – 1.83 (m, 8H, $H_{12,21}$), 1.65 – 1.57 (m, 18H, $H_{4,30,35}$), 1.55 – 1.48 (m, 8H, $H_{13,20}$), 1.43 – 1.37 (m, 24H, $H_{14,15,16,17,18,19}$); **^{13}C NMR** (151 MHz, $CDCl_3$) δ 162.94, 157.68, 157.07, 149.14, 149.10, 139.32, 138.26, 138.02, 134.32, 134.18, 133.39, 129.69, 129.58, 129.12, 128.98, 127.85, 127.78, 125.46, 125.44, 125.29, 125.22, 125.20, 124.86, 119.89, 119.82, 118.22, 107.19, 106.75, 69.19, 68.40, 49.31, 29.91, 29.89, 29.73, 29.57, 26.44, 21.91, 21.89, 21.87. Due to high degree of apparent symmetry many ^{13}C signals overlap. **HRMS** (ESI$^+$): Calcd. for $C_{123}H_{134}N_9O_{12}$: 1930.0180, found 1930.0123 $[M+H]^+$.

7

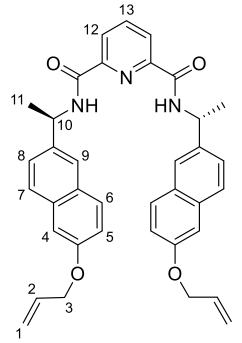

To a solution of **S14** (65 mg, 0.13 mmol) and potassium carbonate (88 mg, 0.64 mmol) in degassed DMF (13 mL) was added allyl bromide (32 µl, 0.38 mmol). The reaction was stirred for 16 h at room temperature, after which the mixture was concentrated under reduced pressure. Flash column chromatography (CH_2Cl_2/EtOAc 3:1) yielded the pure compound **7** as a colourless solid (47 mg, 63 %). **^{1}H NMR** (600 MHz, $CDCl_3$) δ 8.37 (d, *J* = 7.8 Hz, 2H, H_{12}), 8.04 (t, *J* = 7.8 Hz, 1H, H_{13}), 7.85 (d, *J* = 8.1 Hz, 2H, *N-H*), 7.74 (s, 2H, H_9), 7.69 (d, *J* = 8.9 Hz, 2H, H_6), 7.64 (d, J = 8.5 Hz, 2H, H_7), 7.44 (dd, *J* = 1.9, 8.5 Hz, 2H, H_8), 7.20 (dd, *J* = 2.5, 8.9 Hz, 2H, H_5), 7.13 – 7.12 (m, 2H, H_4), 6.18 – 6.10 (m, 2H, H_2), 5.84 (m, 2H, H_{1trans}), 5.44 (quintet, *J* = 7.3 Hz, 2H, H_{10}), 5.34 (m, 2H, H_{1cis}), 4.68 – 4.66 (m, 4H, H_3), 1.67 (d, *J* = 6.9 Hz, 6H, H_{11}); **^{13}C NMR** (151 MHz, $CDCl_3$) δ 162.80, 157.02, 149.06, 139.31, 138.13, 134.14, 133.28, 129.64, 129.08, 127.80, 125.40, 125.14, 124.83, 119.76, 118.13, 107.13, 69.10, 49.31, 21.87. Due to high degree of apparent symmetry many ^{13}C signals overlap. **HRMS** (ESI$^-$): Calcd. for $C_{37}H_{36}N_3O_4$: 586.2700, found 586.2700 [M+H$^-$]$^-$

8

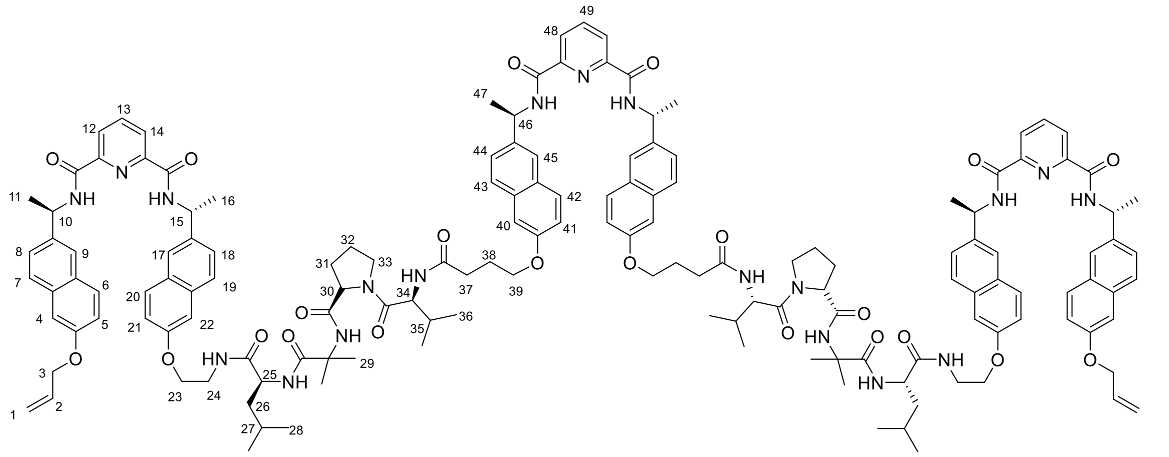

The compound **A5** (253 mg, 0.15 mmol) and $Pd(OH)_2$ on carbon (35 wt%) were stirred overnight in MeOH (10 mL) at 35 °C under an atmosphere of H_2. The reaction mixture was filtered through Celite® and concentrated under reduced pressure to give the deprotected peptide as a colourless solid, which was carried through crude to the next step without further purification. To a stirred solution of the crude product and HATU (118 mg, 0.31 mmol) in DMF (4 mL) was added **A3** (222.6 mg, 0.36 mmol) in DMF (4 mL), followed by DIPEA (112 µL, 0.64 mmol). The reaction mixture was left stirring at room temperature for 5 hours, after which the mixture was diluted with ethyl acetate (10 mL). The suspension was washed with lithium chloride (aq 5%, 5 mL). and extracted with fresh EtOAc (3 × 10 mL), followed by drying over

sodium sulphate and concentration under reduced pressure to give the crude product as a yellow oil. Flash column chromatography (CH_2Cl_2/MeOH 35:1) yielded the pure compound **8** as an off-white solid (234 mg, 78%). **^{1}H NMR** (500 MHz, DMSO-*d$_6$*) δ = 9.43 (d, *J* = 8.4, 6H, *N-H*), 8.31 (br s, 1H, *N-H*), 8.24 – 8.22 (m, 2H, H_{48}), 8.22 – 8.21 (m, 4H, $H_{12,14}$), 8.19 – 8.14 (m, 3H, $H_{13,49}$), 7.87 – 7.76 (m, 20H, $H_{6,7,9,17,18,19,20,42,43,45}$), 7.58 - 7.53 (m, 6H, $H_{8,18,44}$), 7.42 (m, 2H, *N-H*), 7.31 (d, *J* = 2.4, 2H, H_{40}), 7.26 (m, 4H, $H_{4,22}$), 7.17 (m, 2H, H_{41}), 7.15 - 7.07 (m, 4H, $H_{5,21}$), 6.10 (m, 2H, H_2), 5.48 - 5.34 (m, 8H, $H_{1trans,10,15,46}$), 5.29 (m, 2H, H_{1cis}), 4.67 (m, 4H, H_3), 4.36 (app t, *J* = 7.8, 2H, H_{30}), 4.21 (m, 2H, H_{34}), 4.15 (m, 2H, H_{25}), 4.08 – 4.01 (m, 8H, $H_{23,39}$), 3.72 – 3.68 (m, 2H, H_{33}), 3.61 – 3.54 (m, 2H, H_{33}), 3.48 (m, 2H, H_{24}), 3.38 - 3.35 (m, 2H, H_{24}), 2.45 (m, 2H, H_{37}), 2.31 (m, 2H, H_{37}), 2.02 – 1.90 (m, 12H, $H_{31,32,38}$), 1.78 – 1.74 (m, 2H, H_{35}), 1.69 – 1.62 (m, 22H, $H_{11,16,26,47}$), 1.58 - 1.53 (m, 2H, H_{27}), 1.33 (s, 6H, H_{29}), 1.31 (s, 6H, H_{29}), 0.89 – 0.79 (m, 24H, $H_{28,36}$); **^{13}C NMR** (126 MHz, DMSO-*d$_6$*) δ = 174.48, 174.26, 173.77, 172.66, 172.34, 171.87, 170.74, 162.84, 156.38, 156.24, 155.93, 149.13, 139.37, 139.00, 138.91, 138.85, 133.59, 133.29, 133.24, 129.64, 129.22, 129.11, 129.07, 128.22, 128.20, 128.13, 126.89, 126.83, 125.54, 125.49, 124.73, 124.04, 118.85, 117.58, 106.91, 106.58, 106.44, 68.21, 67.00, 66.32, 65.82, 59.91, 56.09, 51.42, 48.13, 47.36, 38.34, 31.26, 29.80, 29.08, 29.02, 26.55, 26.34, 24.79, 24.50, 24.46, 24.01, 23.13, 21.68, 21.11, 20.44, 19.02, 18.23. Due to high degree of apparent symmetry many ^{13}C signals overlap; **MALDI-TOF** (+): Calcd. for $C_{151}H_{175}N_{19}O_{22}Na$: 2629.3, found 2628.8 $[M+Na]^+$.

1•[Lu]

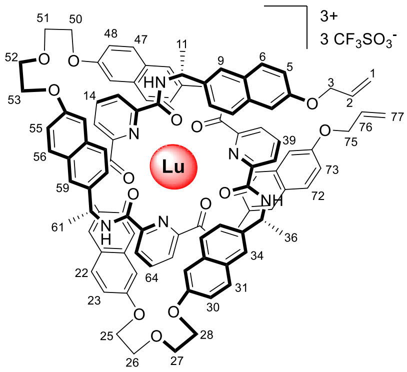

To a solution of **1** (30 mg, 0.017 mmol) in MeCN (17 mL) was added lutetium trifluoromethanesulfonate (10.7 mg, 0.017 mmol). The solution was stirred at 80 °C for 16 h. The mixture was allowed to cool to room temperature, filtered and concentrated under reduced pressure. The crude was purified using size exclusion chromatography (Sephadex LH-20, MeOH) to give **1**•[Lu] as a colourless solid (5 mg, 12 %). The yield is representative of the size exclusion fractions of the highest purity. **^{1}H NMR** (600 MHz, CD_3CN) δ 8.43 (d, *J* = 4.8 Hz, 2H, $H_{12/16/37/41/62/66}$), 8.39 (d, *J* = 3.8 Hz, 2H, $H_{12/16/37/41/62/66}$), 8.22 (d, *J* = 4.8 Hz, 2H, $H_{12/16/37/41/62/66}$), 7.65 (d, *J* = 8.8 Hz, 2H, $H_{6/22/31/47/56/72}$), 7.60 (d, *J* = 8.0 Hz, 2H, $H_{38,40}$), 7.54 (d, *J* = 8.5 Hz, 2H, $H_{7/21/32/46/57/71}$), 7.52 – 7.47 (m, 4H, $H_{6/22/31/47/56/72,\ 7/21/32/46/57/71}$), 7.34 – 7.20 (m, 15H, $H_{4/24/29/49/54/74,\ 5/23/30/48/55/73,\ 6/22/31/47/56/72,\ 7/21/32/46/57/71,\ 8/20/33/45/58/70,\ 39}$), 7.11 (dd, *J* = 8.9, 2.1 Hz, 2H, $H_{5/23/30/48/55/73}$), 7.00 (s, 2H, $H_{4/24/29/49/54/74}$), 6.95 (s, 2H, $H_{9/19/34/44/59/69}$), 6.92 (s, 2H, $H_{9/19/34/44/59/69}$), 6.90 – 6.83 (m, 6H,

$H_{8/20/33/45/58/70,\ 9/19/34/44/59/69,\ 13/15/63/65}$), 6.66 (d, *J* = 7.3 Hz, 2H, $H_{13/15/63/65}$), 6.57 (d, *J* = 7.4 Hz, 2H, $H_{8/20/33/45/58/70}$), 6.24 (ddt, *J* = 17.9, 10.5, 5.2 Hz, 2H, $H_{2,76}$), 5.82 (t, *J* = 7.9 Hz, 2H, $H_{14,64}$), 5.57 (app dd, *J* = 17.3, 1.6 Hz, 2H, $H_{1,77(trans)}$), 5.40 (app dd, *J* = 10.6, 1.4 Hz, 2H, $H_{1,77(cis)}$), 4.80 – 4.72 (m, 6H, $H_{3,35,42,75}$), 4.67 – 4.61 (m, 4H, $H_{10,17,60,67}$), 4.56 – 4.51 (m, 2H, $H_{25/28/50/53}$), 4.47 – 4.42 (m, 2H, $H_{25/28/50/53}$), 4.38 – 4.29 (m, 4H, $H_{25/28/50/53}$), 4.15 – 4.0 (m, 8H, $H_{26,27,51,52}$), 1.62 (d, *J* = 7.1 Hz, 6H, $H_{11/18/36/43/61/68}$), 1.56 (d, *J* = 7.1 Hz, 6H, $H_{11/18/36/43/61/68}$), 1.46 (d, *J* = 7.0 Hz, 6H, $H_{11/18/36/43/61/68}$); **^{13}C NMR** (151 MHz, CD_3CN) δ 167.22, 166.93, 166.66, 157.32, 157.23, 157.12, 145.09, 144.06, 143.92, 141.61, 139.10, 139.08, 139.03, 138.74, 134.19, 134.13, 133.92, 133.45, 129.75, 129.39, 128.93, 128.92, 128.49, 127.75, 127.71, 127.38, 124.88, 124.26, 123.72, 123.50, 123.10, 119.98, 119.87, 119.76, 107.82, 107.61, 70.04, 69.81, 69.32, 68.09, 68.04, 54.17, 53.59, 52.59, 23.38, 23.09, 21.71. Due to high degree of apparent symmetry many ^{13}C signals overlap. **HRMS** (ESI+): Calcd. for $C_{108}H_{101}N_9O_{17}F_3SLu$: 1029.8192, found 1029.8151 $[M-(CF_3SO_3^-)_2]^{2+}$.

3•[Lu]

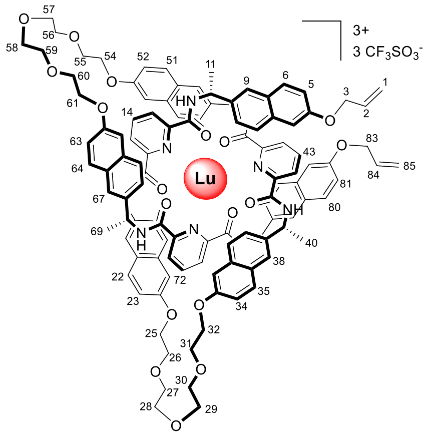

To a solution of **3** (1.5 mg, 0.785 µmol) in MeCN (0.5 mL) was added lutetium trifluoromethanesulfonate (0.5mg, 0.785 µmol). The solution was stirred at 80 °C for 48 h. The mixture was allowed to cool to room temperature, filtered, concentrated and triturated twice with CH_2Cl_2 to give **3**•[Lu] as a colourless solid (1.2 mg, 76% yield). **^{1}H NMR** (600 MHz, CD_3CN) δ 8.58 - 8.55 (m, 4H, $H_{12/16/41/45/70/74}$), 8.50 (d, *J* = 5.8 Hz, 2H, $H_{12/16/41/45/70/74}$), 7.64 (d, *J* = 8.9 Hz, 2H, $H_{6/22/35/51/64/80}$), 7.59 – 7.55 (m, 4H, $H_{6/22/35/51/64/80}$), 7.59 – 7.54 (m, 4H, $H_{7/21/36/50/65/79}$), 7.54 – 7.52 (m, 2H, $H_{42,44}$), 7.40 - 7.35 (m, 4H, $H_{4/7/21/24/33/36/50/53/62/65/79/82}$), 7.34 – 7.25 (m, 6H, $H_{5,23,34,52,63,81}$), 7.25 – 7.23 (m, 2H, $H_{9/19/38/48/67/77}$), 7.20 – 7.15 (m, 4H, $H_{4/24/33/53/62/82}$), 7.11 – 7.06 (m, 4H, $H_{13,15,71,73}$), 7.06 – 7.05 (m, 1H, H_{43}), 7.05 – 6.96 (m, 4H, $H_{9/19/38/48/67/77}$), 6.83 - 6.78 (m, 4H, $H_{8/20/37/49/66/78}$), 6.67 (dd, *J* = 8.4, 1.8 Hz, 2H, $H_{8/20/37/49/66/78}$), 6.31 – 6.24 (m, 2H, $H_{2,84}$), 6.20 (t, *J* = 7.9 Hz, 2H, $H_{14,72}$), 5.63 - 5.58 (m, 2H, $H_{1,85(trans)}$), 5.45 - 5.41 (m, 2H, $H_{1,85(cis)}$), 4.83 - 4.77 (m, 8H, $H_{3,17,39,46,68,83}$), 4.71 (quintet, *J* = 6.4 Hz, 2H, $H_{10,75}$), 4.44 – 4.37 (m, 4H, $H_{25/32/54/61}$), 4.32 – 4.25 (m, 4H, $H_{25/32/54/61}$), 4.14 – 4.08 (m, 4H, $H_{26/31/55/60}$), 4.01 – 3.96 (m, 4H, $H_{26/31/55/60}$), 3.83 – 3.79 (m, 8H, $H_{27,30,56,59}$), 3.78 – 3.72 (m, 8H, $H_{28,29,57,58}$), 1.59 (d, *J* = 7.1 Hz, 6H, $H_{11/18/40/47/69/76}$), 1.55 (d, *J* = 7.1 Hz, 6H, $H_{11/18/40/47/69/76}$), 1.52 (d, *J* = 7.1 Hz, 6H, $H_{11/18/40/47/69/76}$); **^{13}C NMR** (151 MHz, CD_3CN) δ 166.61, 166.41, 166.24, 157.10, 157.01, 156.78, 149.44, 144.38, 143.82, 143.77, 140.68, 139.52,

138.38, 138.34, 138.12, 137.75, 133.65, 133.60, 133.47, 133.30, 129.93, 129.89, 129.18, 129.10, 129.01, 128.97, 128.96, 128.48, 128.40, 128.36, 128.22, 127.23, 127.21, 127.06, 125.84, 125.39, 124.19, 124.07, 123.43, 123.33, 123.18, 122.83, 122.72, 122.39, 122.26, 121.95, 119.83, 119.46, 119.40, 107.28, 106.97, 70.68, 70.57, 70.26, 70.23, 69.44, 69.40, 68.76, 67.99, 52.78, 52.46, 51.98, 22.22, 22.12, 21.17; Due to the symmetry of the molecule, several carbon signals could not be distinguished due to overlapping peaks; **HRMS** (ESI+): Calcd. for $C_{116}H_{117}N_9O_{21}F_3SLu$: 1117.8717, found 1117.8670 $[M-(CF_3SO_3^-)_2]^{2+}$.

4•[Lu]

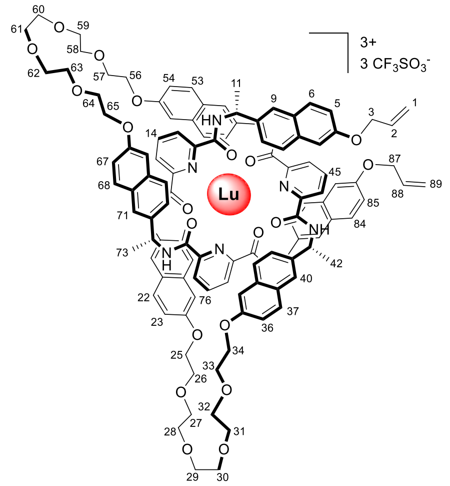

To a solution of **4** (7.0 mg, 3.5 µmol) in MeCN(1.75 mL) was added lutetium trifluoromethanesulfonate (2.2 mg, 3.5 µmol). The solution was stirred at 80 °C for 48 h. The mixture was allowed to cool to room temperature, filtered, concentrated and triturated twice with CH_2Cl_2 to give **4**•[Lu] as a colourless solid (4.9 mg, 64% yield). **^{1}H NMR** (600 MHz, CD_3CN) δ 8.59 (d, *J* = 5.9 Hz, 2H, $H_{12/16/43/47/74/78}$), 8.47 (d, *J* = 6.0 Hz, 2H, $H_{12/16/43/47/74/78}$), 8.47 (d, *J* = 6.0 Hz, 2H, $H_{12/16/43/47/74/78}$), 7.59 (dd, 4H, $H_{6/22/37/53/68/84}$), 7.54 (d, *J* = 8.0 Hz, 2H, $H_{44,46}$), 7.50 (d, *J* = 8.7 Hz, 4H, $H_{7/21/38/52/69/83}$), 7.38 (d, *J* = 8.5 Hz, 2H, $H_{6/22/37/53/68/84}$), 7.31 – 7.28 (m, 6H, $H_{5,23,36,54,67,85}$), 7.26 – 7.21 (m, 6H, $H_{4,24,35,55,66,86}$), 7.18 (dd, *J* = 8.9, 2.5 Hz, 2H, $H_{7/21/38/52/69/83}$), 7.14 (d, *J* = 8.0 Hz, 2H, $H_{13/15/75/77}$), 7.07 (t, *J* = 8.0 Hz, 1H, H_{45}), 7.03 (d, *J* = 4.0 Hz, 2H, $H_{13/15/75/77}$), 7.02 – 6.98 (m, 4H, $H_{9/19/40/50/71/81}$), 6.90 (d, *J* = 2.2 Hz, 1H, $H_{9/19/40/50/71/81}$), 6.78 – 6.74 (m, 5H, $H_{8/20/39/51/70/82,9/19/40/50/71/81}$), 6.68 (dd, *J* = 8.5, 1.9 Hz, 2H, $H_{8/20/39/51/70/82}$), 6.56 (t, *J* = 7.9 Hz, 2H, $H_{14,76}$), 6.28 (ddt, *J* = 17.3, 10.6, 5.3 Hz, 2H, $H_{2,88}$), 5.62 - 5.58 (m, 2H, $H_{1,89(trans)}$), 5.45 - 5.41 (m, 2H, $H_{1,89(cis)}$), 4.82 - 4.77 (m, 6H, $H_{3,10/17/41/48/72/79/87}$), 4.75 – 4.68 (m, 4H, $H_{10/17/41/48/72/79}$), 4.41 – 4.33 (m, 8H, $H_{25,34,56,65}$), 4.04 – 3.98 (m, 8H, $H_{26,33,57,64}$), 3.82 – 3.78 (m, 8H, $H_{27,32,58,63}$), 3.76 – 3.70 (m, 8H, $H_{28,31,59,62}$), 3.69 - 3.67 (m, 8H, $H_{29,30,60,61}$), 1.57 (d, *J* = 7.0 Hz, 6H, $H_{11/18/42/49/73/80}$), 1.55 – 1.51 (m, 12H, $H_{11/18/42/49/73/80}$); **^{13}C NMR** (151 MHz, CD_3CN) δ 166.44, 166.36, 166.22, 157.02, 156.97, 156.80, 149.42, 144.40, 143.91, 143.82, 140.21, 138.30, 138.28, 137.94, 137.74, 133.61, 133.44, 133.34, 129.89, 129.17, 129.01, 128.95, 128.93, 128.48, 128.39, 128.26, 128.19, 127.24, 127.21, 127.17, 125.84, 125.39, 124.20, 123.50, 123.47, 123.25, 122.89, 122.46, 122.32, 119.49, 119.37, 119.30, 107.24, 107.03, 106.99, 70.64, 70.47, 70.44, 69.29, 68.76, 67.66, 67.63, 52.28, 52.22, 51.94, 21.79, 21.03, 19.88; Due to the symmetry of the molecule, several carbon signals could not be distinguished

due to overlapping peaks; **HRMS** (ESI+): Calcd. for $C_{120}H_{125}N_9O_{23}F_3SLu$: 1161.8979, found 1161.8921 [M-($CF_3SO_3^-$)$_2$]$^{2+}$.

5•[Lu]

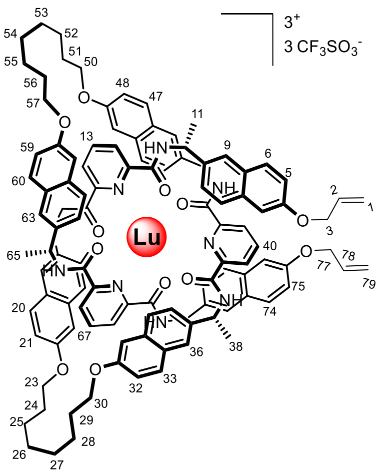

To a vigorously stirred solution of **5** (80 mg, 0.045 mmol) in MeCN (44 mL) was added lutetium trifluoromethanesulfonate (27.4 mg, 0.045 mmol). The reaction was stirred at 80 °C for 48 h. The mixture was allowed to cool to room temperature, filtered and concentrated under reduced pressure. The solid was repeatedly triturated with dichloromethane to give **5•**[Lu] as an off-white powder (57 mg, 53 %). **^{1}H NMR** (600 MHz, CD_3CN) δ 8.60 (d, *J* = 5.2 Hz, 2H, *N-H*), 8.48 – 8.43 (m, 4H, *N-H*), 7.69 – 7.58 (m, 6H, $H_{6,39,41,61,73,74}$), 7.55 – 7.48 (m, 4H, $H_{7,19,20,60}$), 7.36 – 7.19 (m, 14H, $H_{5,21,22,31,33,34,36,44,46,47,49,58,59,75}$), 7.16 (t, *J* = 7.9 Hz, 1H, H_{40}), 7.13 – 7.08 (m, 4H, $H_{4,32,48,76}$), 7.06 – 7.03 (m, 2H, $H_{12/14/66/68}$), 6.99 (s, 2H, $H_{9/17/63/71}$), 6.96 – 6.92 (m, 2H, $H_{12/14/66/68}$), 6.91 (s, 2H, $H_{9/17/63/71}$), 6.85 – 6-81 (m, 4H, $H_{8,18,62,72}$), 6.73 – 6.69 (m, 2H, $H_{35,45}$), 6.31 - 6.23 (m, 6H, $H_{2,13,67,78}$), 5.60 (m, 2H, $H_{1,79trans}$), 5.43 (m, 2H, $H_{1,79cis}$), 4.84 – 4.77 (m, 6H, $H_{3,37,42,77}$), 4.74 – 4.67 (m, 4H, $H_{10,15,64,69}$), 4.33 – 4.22 (m, 4H, $H_{23/30/50/57}$), 4.19 – 4.09(m, 4H, $H_{23/30/50/57}$), 2.13 – 2.05 (m, 4H, $H_{24/29/51/56}$), 2.01 – 1.92 (m, 4H, $H_{24/29/51/56}$), 1.80 – 1.68 (m, 12H, $H_{25/26/27/28/52/53/54/55}$), 1.64 – 1.59 (m, 10H, $H_{11/16/38/43/65/70/25/26/27/28/52/53/54/55}$), 1.56 (d, *J* = 7 Hz, 6H, $H_{11/16/38/43/65/70}$), 1.52 (d, *J* = 7 Hz, 6H, $H_{11/16/38/43/65/70}$); **^{13}C NMR** (151 MHz, CD_3CN) δ 167.00, 166.81, 166.59, 157.73, 157.61, 157.22, 144.89, 144.23, 144.08, 141.20, 139.62, 138.59, 138.57, 138.41, 134.05, 133.86, 133.66, 129.61, 129.26, 129.09, 128.83, 128.54, 128.37, 127.63, 127.47, 124.73, 124.00, 123.72, 123.66, 123.46, 123.35, 122.06, 122.01, 119.97, 119.92, 119.81, 107.69, 107.07, 69.18, 68.59, 68.57, 53.24, 52.92, 52.40, 49.33, 29.60, 27.91, 27.87, 27.62, 27.57, 25.45, 25.42, 22.52, 22.47, 21.44. Due to high degree of apparent symmetry many ^{13}C signals overlap. **HRMS** (ESI$^+$): Calcd. for $C_{115}H_{117}N_9O_{12}Lu$: 663.9415, found 663.9401 [M-3(CF_3SO_3)]$^{3+}$.

6•[Lu]

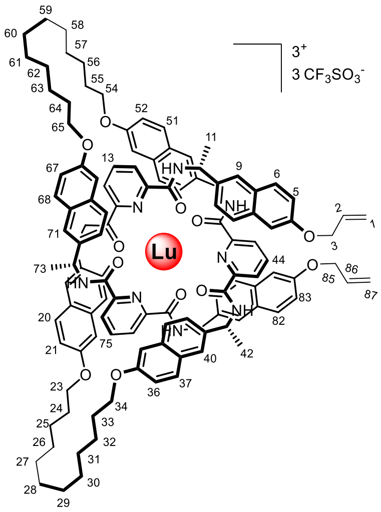

To a vigorously stirred solution of **6** (8.8 mg, 4.5 µmol) in MeCN (4.5 mL) was added lutetium trifluoromethanesulfonate (2.8 mg, 4.5 µmol). The reaction was stirred at 80 °C for 2 days. The mixture was allowed to cool to room temperature, filtered and concentrated under reduced pressure. The solid was repeatedly triturated with dichloromethane to give **6**•[Lu] as an off-white powder (6.1 mg, 52%). **^{1}H NMR** (600 MHz, CD_3CN) δ 8.61 – 8.55 (m, 6H, *N-H*), 7.52 – 7.37 (m, 14H, $H_{6,7,19,20,38,43,45,48,50,51,68,69,81,82}$), 7.31 – 7.13 (m, 18H, $H_{4,5,12,14,21,22,35,36,37,40,52,53,66,67,74,76,83,84}$), 7.08 (s, 4H, $H_{9,17,71,79}$), 7.00 – 6.96 (t, *J* = 7.9 Hz, 1H, H_{44}), 6.76 – 6.70 (m, 4H, $H_{13,75,8/18/39/49/70/80}$), 6.67 – 6.64 (m, 2H, $H_{8/18/39/49/70/80}$), 6.62 – 6.59 (m, 2H, $H_{8/18/39/49/70/80}$), 6.31 – 6.22 (m, 2H, $H_{2,86}$), 5.60 (m, 2H, $H_{1,87trans}$), 5.43 (m, 2H, $H_{1,87cis}$), 4.83 – 4.73 (m, 10H, $H_{3,10,15,41,46,72,77,85}$), 4.26 – 4.19 (m, 8H, $H_{23,34,54,65}$), 2.02 – 1.92 (m, 8H, $H_{24,33,55,64}$), 1.70 – 1.62 (m, 8H, $H_{25,32,56,63}$), 1.58 – 1.53 (m, 18H, $H_{11,16,42,47,73,78}$), 1.52 – 1.45 (m, 24H, $H_{26,27,28,29,30,31,57,58,59,60,61,62}$). **^{13}C NMR** (151 MHz, CD_3CN) δ 167.35, 167.25, 167.23, 158.29, 158.26, 157.71, 145.33, 145.15, 145.09, 141.55, 141.09, 138.90, 138.84, 138.83, 134.57, 134.53, 134.51, 134.49, 130.06, 129.89, 129.30, 129.10, 129.07, 128.15, 128.08, 128.04, 125.04, 124.75, 124.70, 124.35, 124.15, 124.10, 124.03, 123.50, 120.47, 120.43, 120.41, 108.15, 107.69, 69.70, 69.16, 52.99, 52.91, 52.88, 29.61, 29.57, 29.16, 29.13, 28.87, 28.83, 28.51, 26.22, 26.19, 22.33, 22.08. Due to high degree of apparent symmetry many ^{13}C signals overlap. **HRMS** (ESI$^+$): Calcd. for $C_{123}H_{133}N_9O_{12}Lu$: 701.3166, found 701.3143[M-3(CF_3SO_3)]$^{3+}$.

7_3•[Lu]

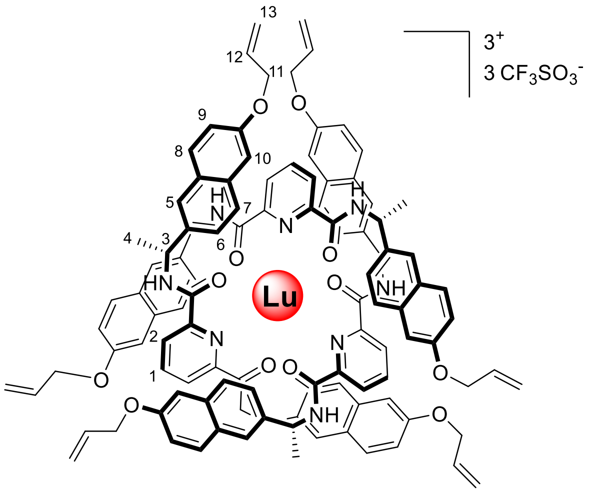

To a vigorously stirred solution of **7** (56 mg, 0.095 mmol) in MeCN (6.33 mL) was added lutetium trifluoromethanesulfonate (19.5 mg, 0.031 mmol). The reaction was stirred overnight at room temperature. The solution was concentrated and the solid was repeatedly triturated with dichloromethane to give **7_3**•[Lu] as an off-white powder (65 mg, 86%). **^{1}H NMR** (600 MHz, CD_3CN) δ 8.54 (d, *J* = 6.4 Hz, 6H, *N-H*), 7.45 – 7.41 (m, 12H, $H_{7,8}$), 7.35 (d, *J* = 8.0 Hz, 6H, H_2), 7.23 – 7.21 (m, 6H, H_5), 7.20 – 7.17 (m, 6H, H_6), 7.10 (s, 6H, H_{10}), 6.87 (t, *J* = 7.9 Hz, 3H, H_1), 6.67 – 6.65 (m, 6H, H_9), 6.28 – 6.19 (m, 6H, H_{12}), 5.57 (m, 6H, $H_{13trans}$), 5.40 (m, 6H, H_{13cis}), 4.79 – 4.70 (m, 18H, $H_{3,11}$), 1.50 (d, *J* = 7.0 Hz, 18H, H_4); **^{13}C NMR** (151 MHz, CD_3CN) δ 167.25, 157.69, 145.30, 141.41, 138.87, 134.57, 134.46, 130.04, 129.27, 128.12, 124.91, 124.30, 123.91, 120.37, 108.12, 69.69, 52.93, 22.12. Due to high degree of apparent symmetry many ^{13}C signals overlap. **HRMS** (ESI$^+$): Calcd. for $C_{111}H_{105}N_9O_{12}Lu$ [M-3(CF_3SO_3)]$^{3+}$: 643.9105, found 643.9082.

8·[Lu]

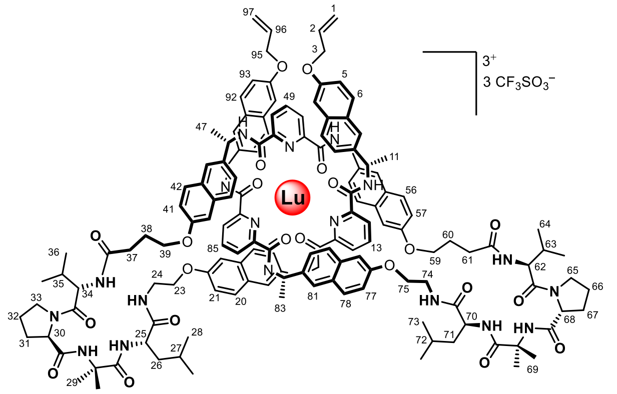

A mixture of **8** (10 mg, 3.83 µmol) and lutetium trifluoromethanesulfonate (2.4 mg, 3.83 µmol) in MeCN was stirred at 80 °C for 8 h under microwave conditions (80 W). The mixture was allowed to cool to room temperature and concentrated under reduced pressure to give the title compound as an off-white solid (12 mg, 97%). **^{1}H NMR** (500 MHz, CD_3CN) δ = 8.60 – 8.51 (m, 6H, *N-H*), 7.61 - 7.55 (m, 4H, $H_{6,92,N\text{-}H}$), 7.54 – 7.42 (m, 12H, $H_{7,19,20,42,48,50,56,78,79,91,N\text{-}H}$), 7.40 - 7.34 (m, 2H, $H_{43,55}$), 7.28 - 7.13 (m, 18H, $H_{4,5,9/17/81/89,21,22,40,41,45,53,57,58,76,77,93,94,N\text{-}H}$), 7.10 - 6.97 (m, 7H, $H_{9/17/81/89,12,14,49,84,86}$), 6.87 - 6.76 (m, 4H, $H_{8,18,80,90}$), 6.62 - 6.58 (m, 2H, $H_{44,54}$), 6.41 (t, *J* = 7.8, 2H, $H_{13,85}$), 6.31 - 6.21 (m, 2H, $H_{2,96}$), 5.63 - 5.56 (m, 2H, $H_{1,97(trans)}$), 5.45 - 5.41 (m, 2H, $H_{1,97(cis)}$), 4.83 – 4.63 (m,10H, $H_{3,10,15,46,51,82,87,95}$), 4.51 (t, *J* = 7.5, 2H, $H_{34,62}$), 4.38 - 4.18 (m, 12H, $H_{23,25,30,39,59,68,70,75}$), 3.96 - 3.80 (m, 4H, $H_{24,33,65,74}$), 3.77 - 3.70 (m, 4H, $H_{33,65}$), 3.60 – 3.51 (m, 4H, $H_{24,74}$), 2.63 (m, 4H, $H_{37,61}$), 2.33 - 2.25 (m, 4H, $H_{38,60}$), 2.21 - 2.13 (m, 10H, $H_{31,32,35,63,66,67}$), 1.85 - 1.77 (m, 4H, $H_{26,71}$), 1.63 -1.50 (m, 26H, $H_{11,16,27,47,52,72,83,88,29/69}$), 1.43 (s, 6H, $H_{29/69}$), 1.02 - 0.99 (m, 12H, $H_{36,64}$), 0.96 - 0.87 (m, 12H, $H_{28,73}$). **^{13}C NMR** (126 MHz, CD_3CN) δ = 175.69, 175.04, 174.33, 174.22, 173.72, 172.17, 167.47, 167.36, 167.18, 157.96, 157.73, 157.70, 150.24, 145.34, 144.70, 144.67, 139.90, 139.89, 139.66, 139.00, 138.90, 134.57, 134.50, 134.35, 134.19, 130.75, 130.11, 130.04, 129.84, 129.26, 129.17, 128.78, 128.13, 128.09, 128.04, 125.00, 124.47, 124.09, 124.04, 124.02, 123.88, 123.51, 123.48, 123.45, 123.35, 123.24, 121.78, 121.40, 121.26, 119.25, 108.67, 108.25, 108.14, 104.78, 69.69, 68.66, 67.73, 61.89, 57.99, 57.45, 53.85, 53.29, 52.89, 48.82, 40.76, 34.11, 32.59, 31.35, 30.31, 30.30, 30.25, 30.14, 30.02, 29.95, 29.81, 29.73, 27.30, 25.77, 25.56, 24.50, 23.35, 21.51, 19.69. Due to high degree of apparent symmetry many ^{13}C signals overlap. **HRMS** (ESI^+): Calcd. for $C_{151}H_{175}N_{19}O_{22}Lu$ $[M-3(CF_3SO_3)]^{3+}$: 927.4207, found 927.4195.

S4.2. Ring-closing metathesis for covalent capture of 8•[Lu]

All alkene-terminated molecular overhand knots can be ring-closed as previously described by us.[6] For metallopeptide knot **8**•[Lu], the procedure is as follows. Compound **8**•[Lu] (9.0 mg, 1.0 eq) and Hoveyda-Grubbs 2nd Generation catalyst (1.75 mg, 1.0 eq) were dissolved in a mixture of freshly degassed CH_2Cl_2 and $MeNO_2$ (3:2, 5 mL). The resulting mixture was left

stirring at 50°C for 24 hours (monitored by ESI-MS). The reaction was quenched with ethyl vinyl ether (1 mL), stirred for 1 h and the reaction mixture was then concentrated under reduced pressure. The residue was washed three times with a mixture of CH_2Cl_2 and diethyl ether, redissolved in methanol and concentrated under reduced pressure. This yielded the target molecule Δ-**9**•[Lu], characterised by ^{1}H NMR spectroscopy (Figure S1) and ESI-MS (Figure S2) which shows full conversion to the desired product.

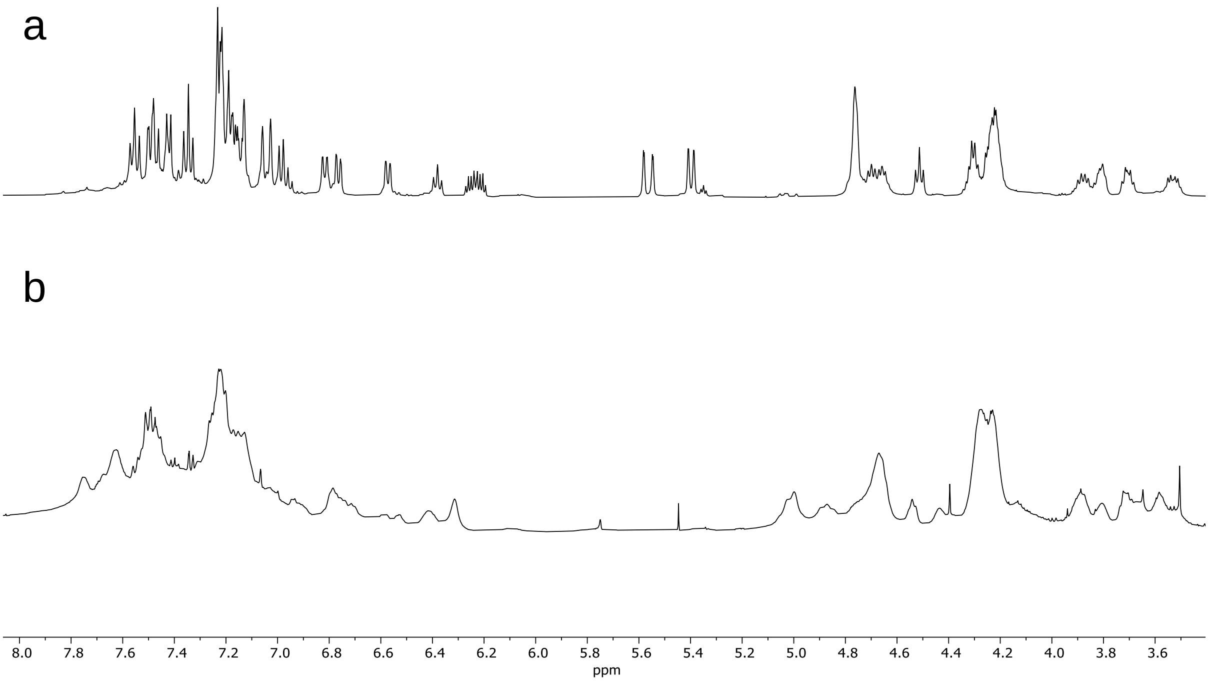

Figure S1. a) ^{1}H NMR spectrum (500 MHz, 298 K, CD_3CN of metallopeptide overhand knot Δ-**8**•[Lu]. b) ^{1}H NMR spectrum (500 MHz, 298 K, CD_3CN) of ring-closed metallopeptide knot Δ-**9**•[Lu].

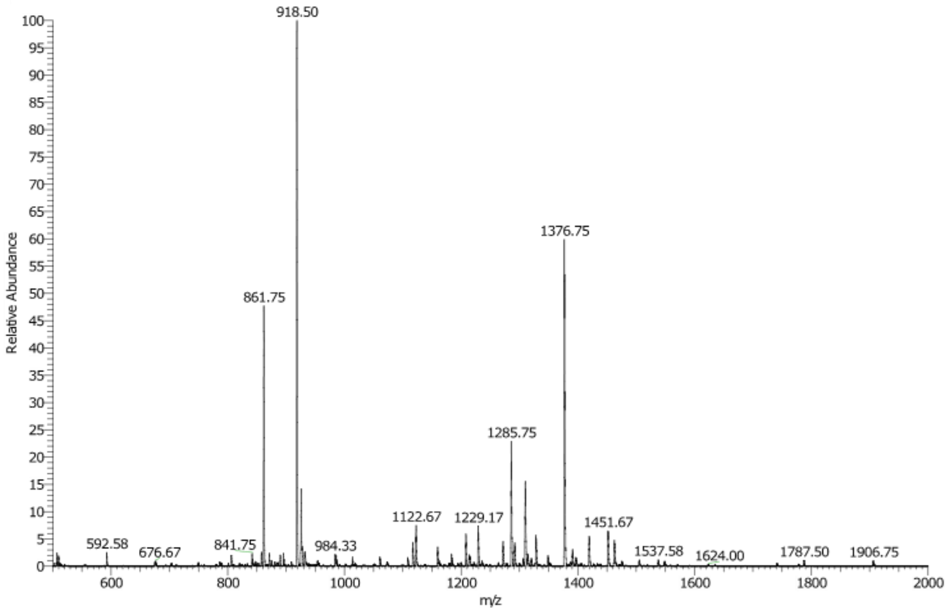

Figure S2. Low resolution ESI-MS (positive mode) of **9•**[Lu] after completion of the ring-closing olefin metathesis reaction. The main peak at m/z 918.5 corresponds to the [M-n(CF_3SO_3)]$^{3+}$ adduct.

S5. MASS SPECTRA

a

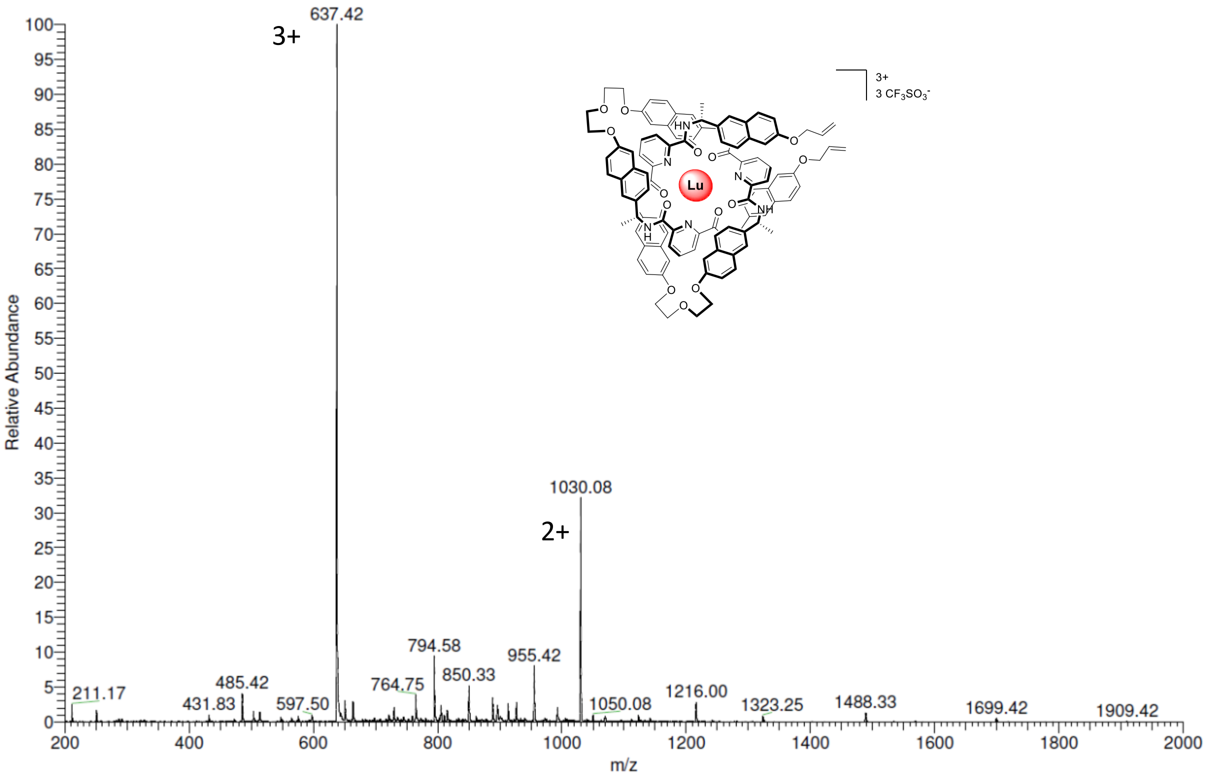

b

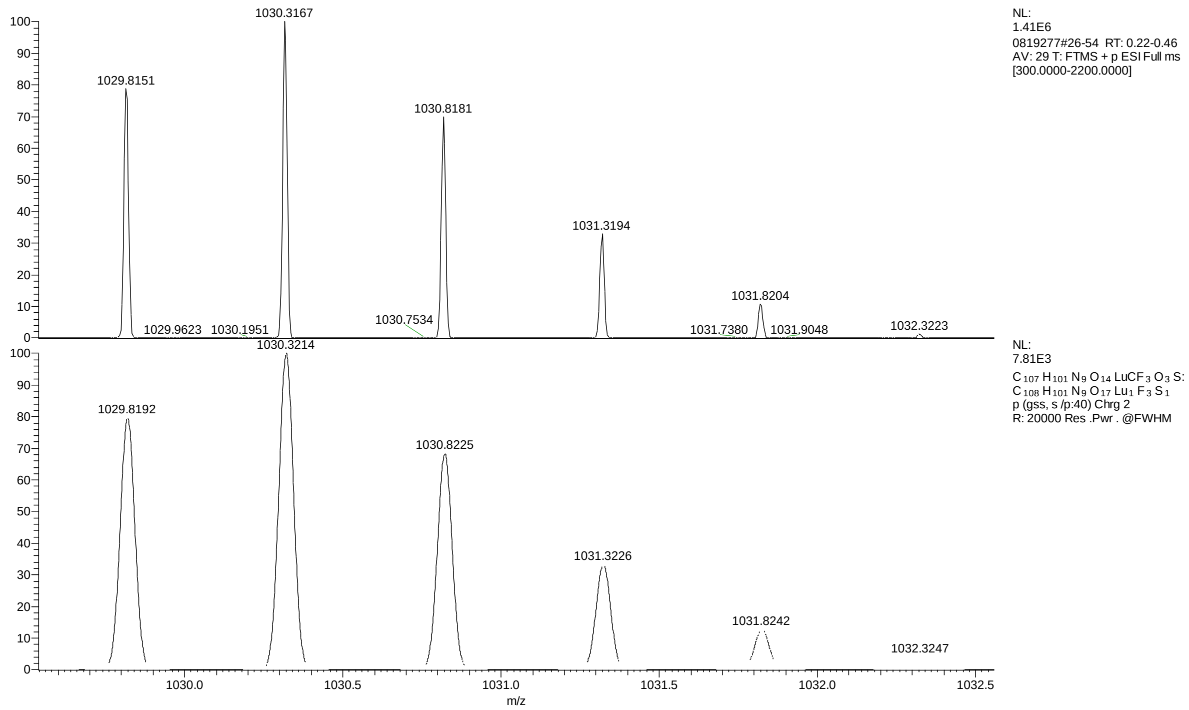

Figure S3. a) Low resolution ESI-MS (positive mode) of **1**•[Lu] (all peaks observed as the $[M\text{-}n(CF_3SO_3)]^{n+}$ adducts). b) High-resolution ESI-MS isotopic distribution of **1**•[Lu] $[M+(CF_3SO_3)]^{2+}$, with observed spectra above and predicted below.

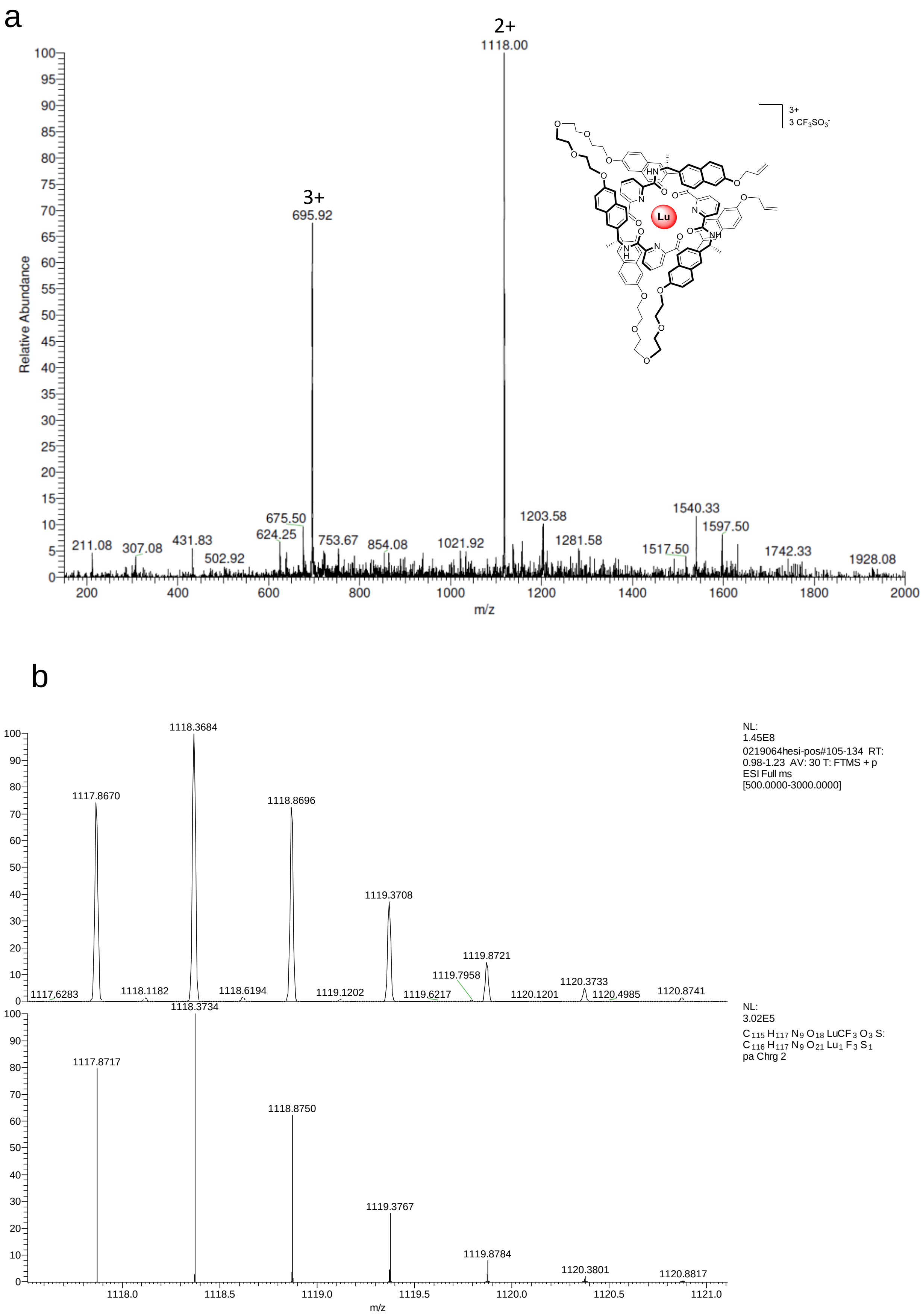

Figure S4. a) Low resolution ESI-MS (positive mode) of **3•**[Lu] (all peaks observed as the [M-n(CF_3SO_3)]$^{n+}$ adducts). b) High-resolution ESI-MS isotopic distribution of **3•**[Lu] [M+(CF_3SO_3)]$^{2+}$, with observed spectra above and predicted below.

a

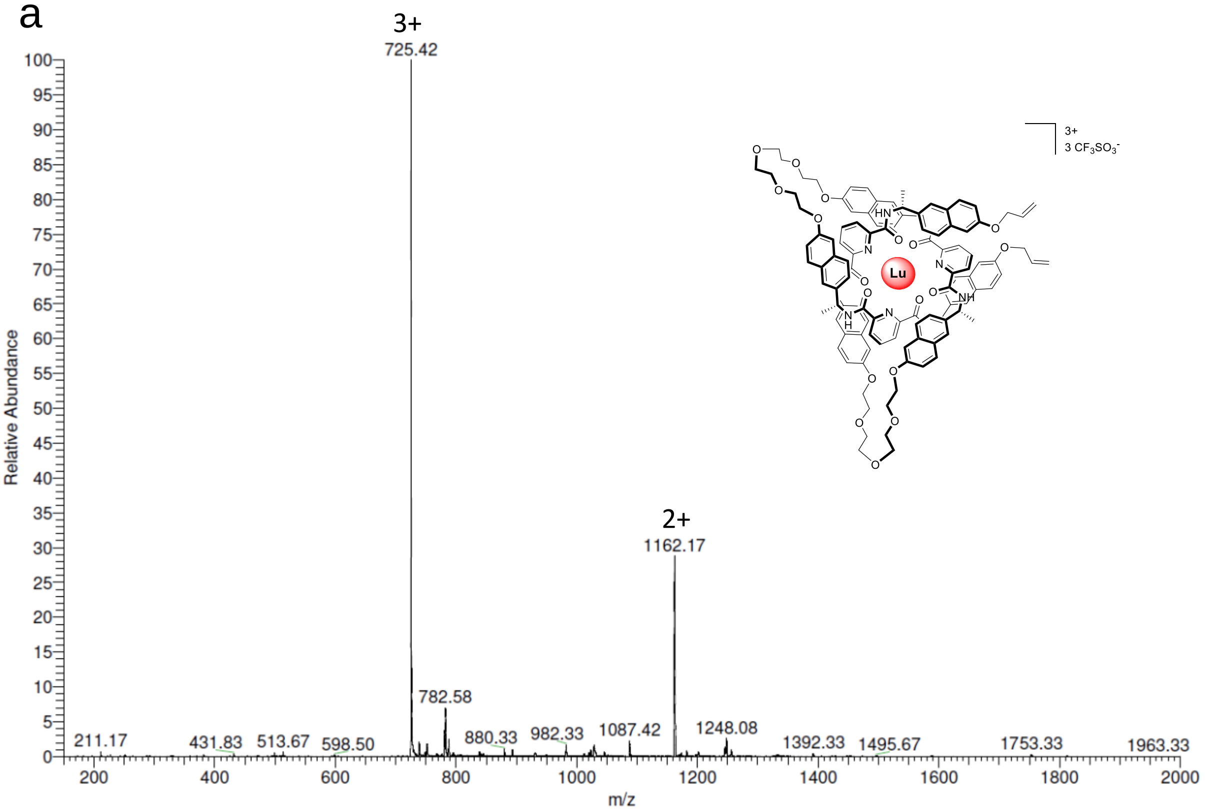

b

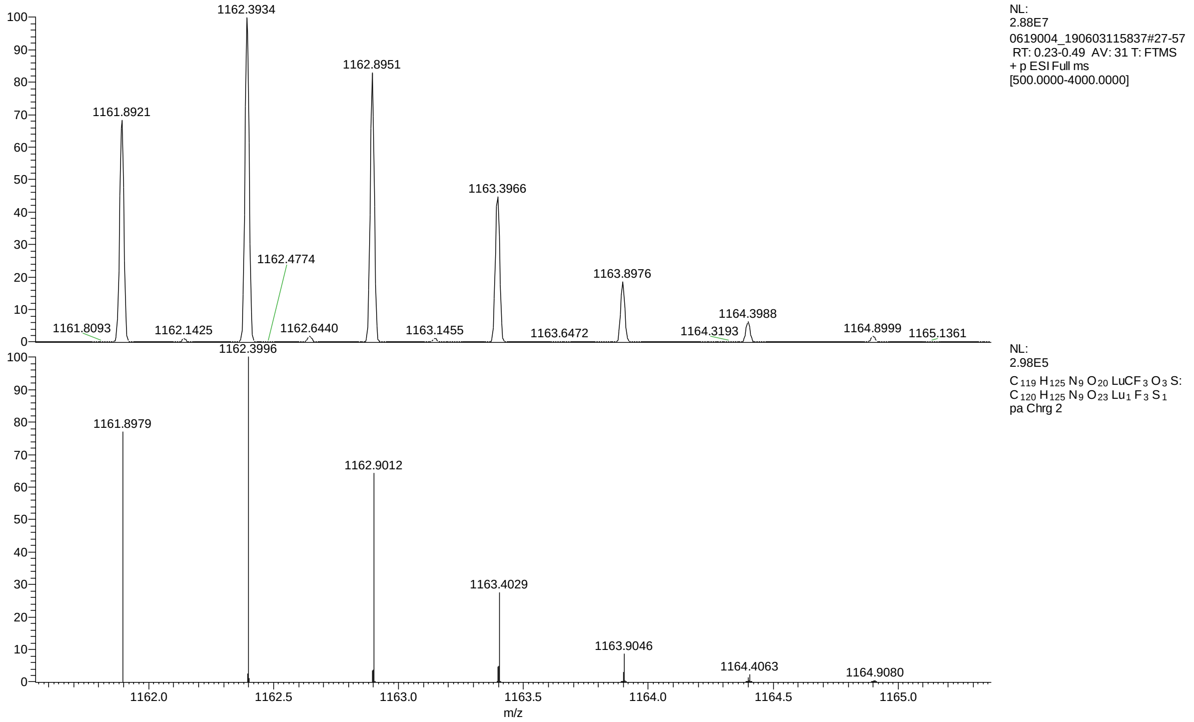

Figure S5. a) Low resolution ESI-MS (positive mode) of **4**•[Lu] (all peaks observed as the [M-n(CF_3SO_3)]$^{n+}$ adducts). b) High-resolution ESI-MS isotopic distribution of **4**•[Lu] [M+(CF_3SO_3)]$^{2+}$, with observed spectra above and predicted below.

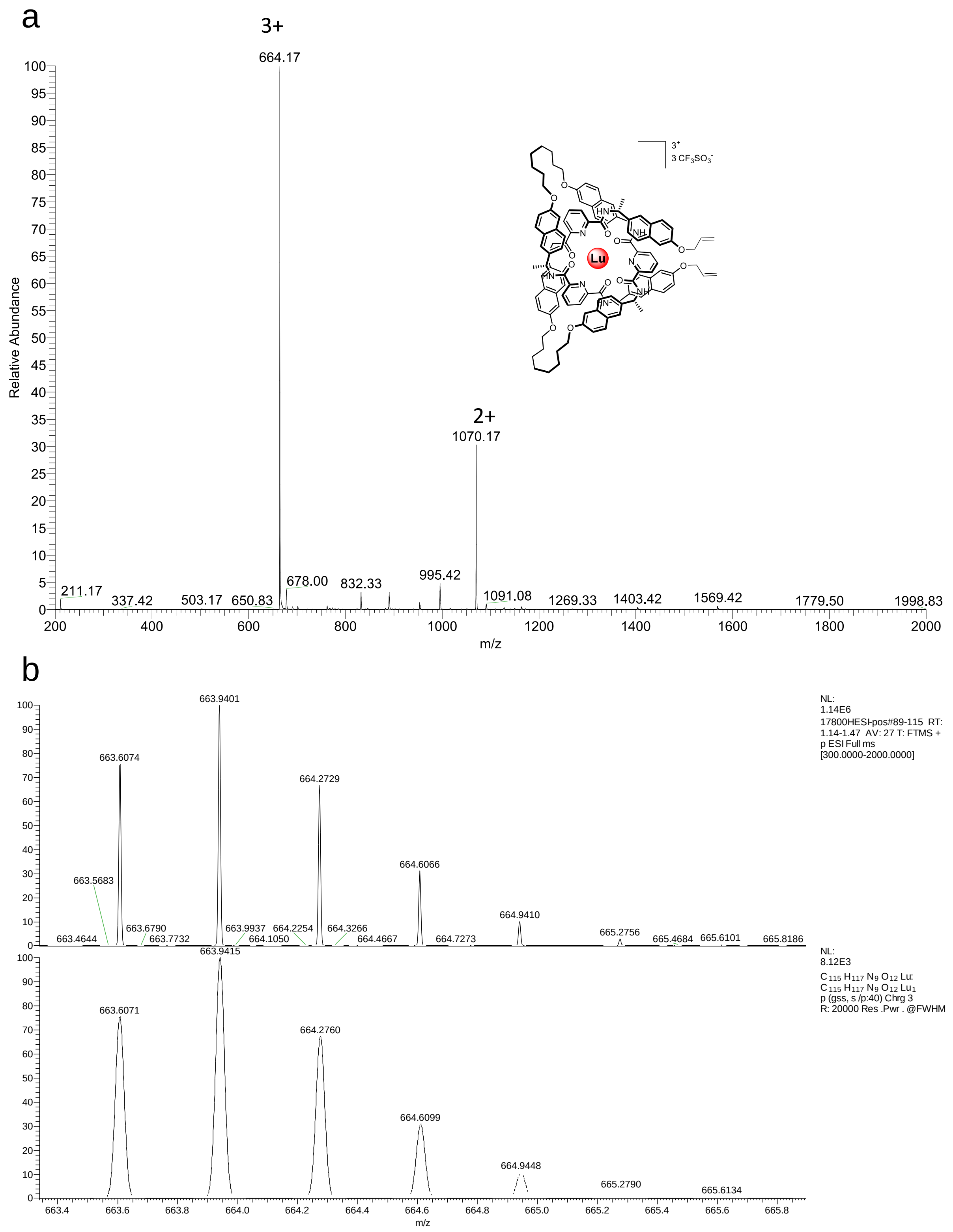

Figure S6. a) Low resolution ESI-MS (positive mode) of **5•**[Lu] (all peaks observed as the $[M\text{-}n(CF_3SO_3)]^{n+}$ adducts). b) High-resolution ESI-MS isotopic distribution of **5•**[Lu] $[M+(CF_3SO_3)]^{2+}$, with observed spectra above and predicted below.

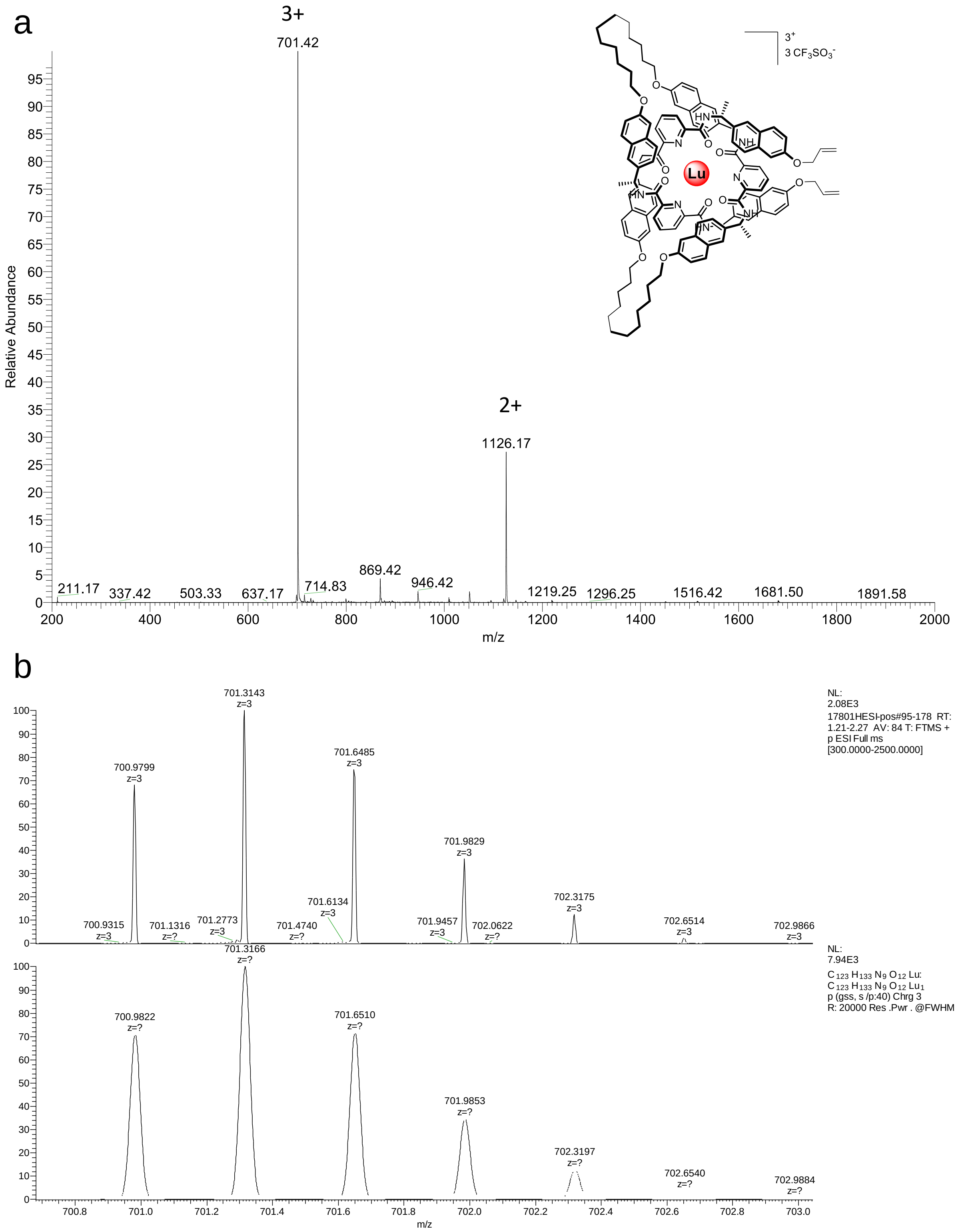

Figure S7. a) Low resolution ESI-MS (positive mode) of **6•**[Lu] (all peaks observed as the [M-n(CF_3SO_3)]$^{n+}$ adducts). b) High-resolution ESI-MS isotopic distribution of **6•**[Lu] [M+(CF_3SO_3)]$^{2+}$, with observed spectra above and predicted below.

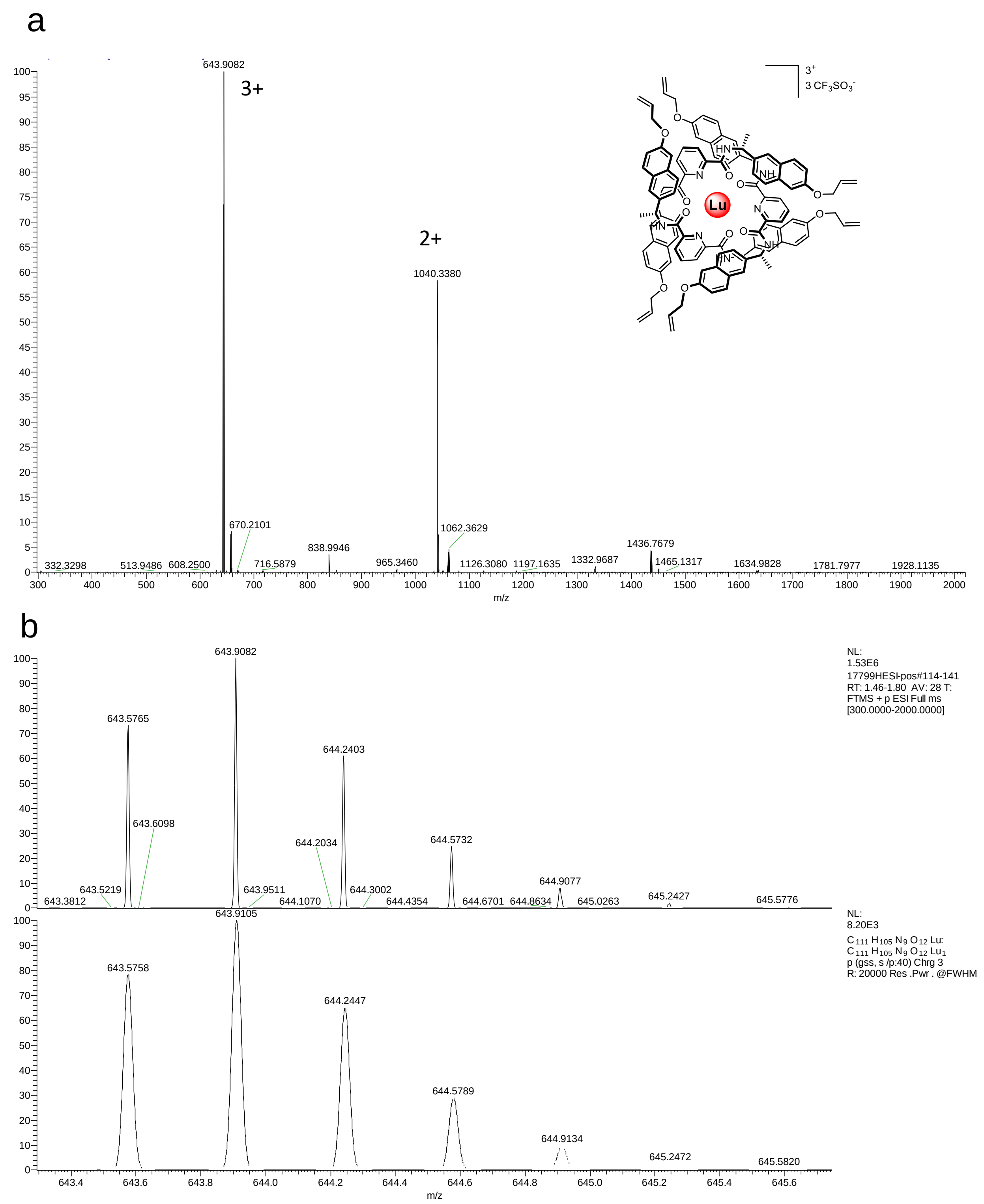

Figure S8. a) Low resolution ESI-MS (positive mode) of **7**$_3$•[Lu] (all peaks observed as the [M- n(CF_3SO_3)]$^{n+}$ adducts). b) High-resolution ESI-MS isotopic distribution of **7**$_3$•[Lu] [M+(CF_3SO_3)]$^{2+}$, with observed spectra above and predicted below.

a

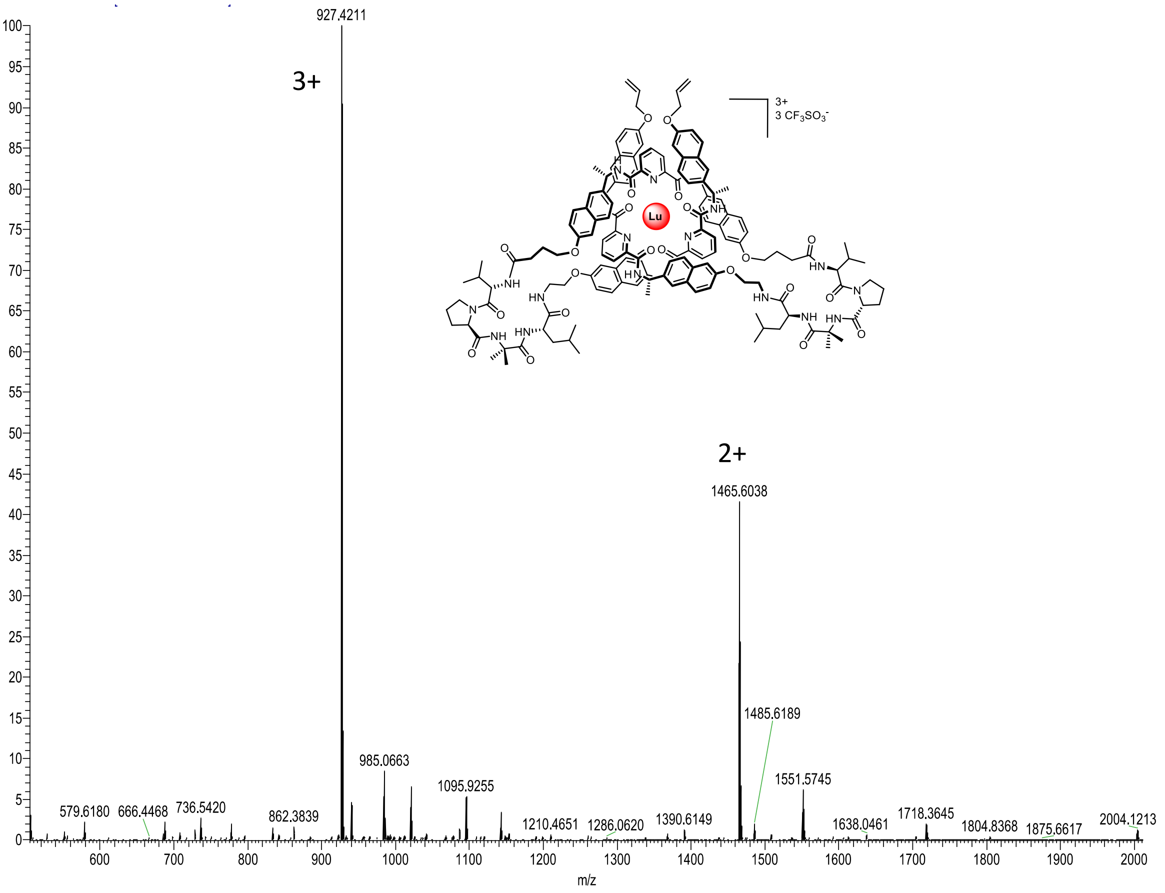

b

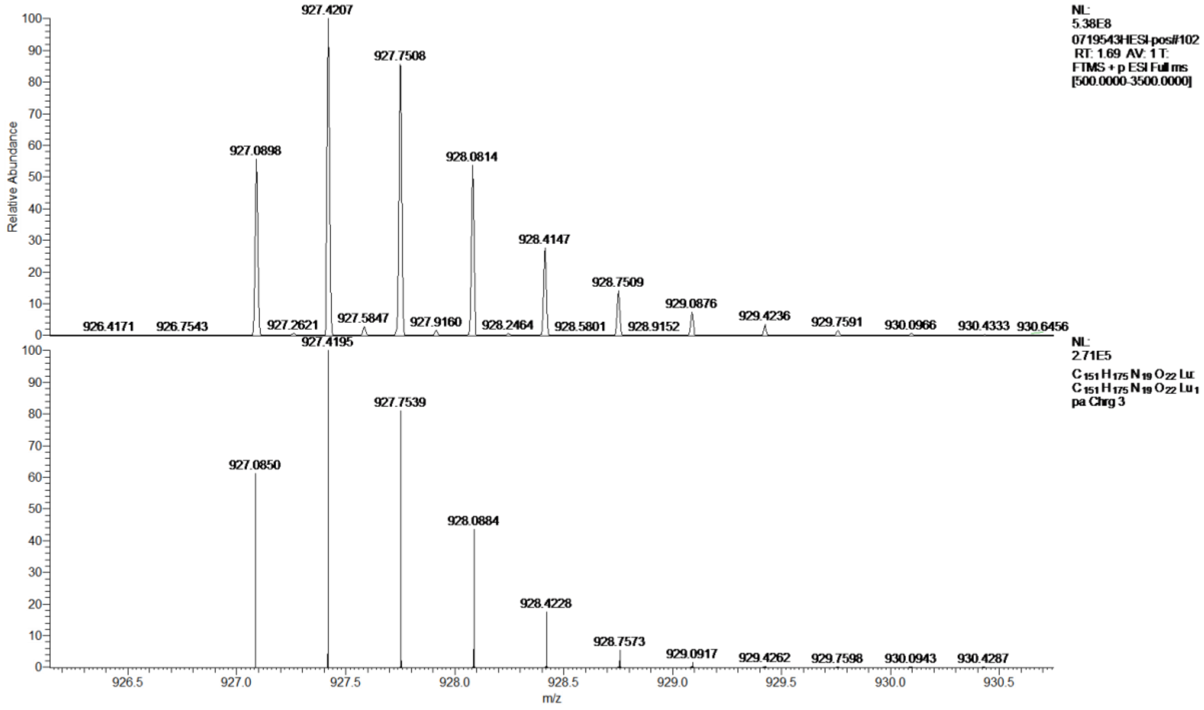

Figure S9. a) Low resolution ESI-MS (positive mode) of **8•**[Lu] (all peaks observed as the $[M\text{-}n(CF_3SO_3)]^{n+}$ adducts). b) High-resolution ESI-MS isotopic distribution of **8•**[Lu]

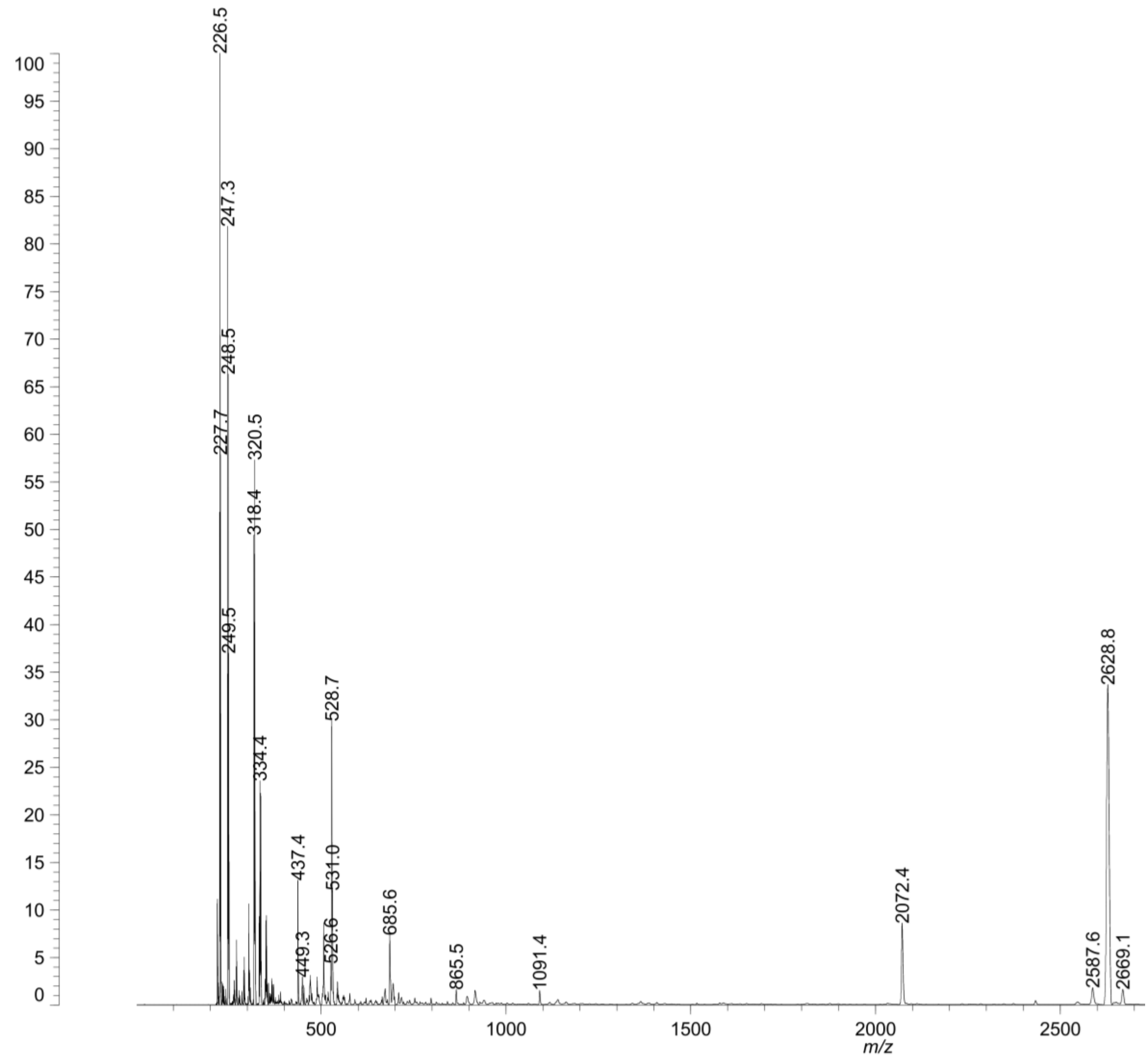

Figure S10. MALDI-TOF MS (positive mode, dithranol matrix) of ligand **8**. Peak observed as the $[M+Na]^+$ adduct. Calculated peak (m/z): 2629.3.

S6. NMR SPECTRA

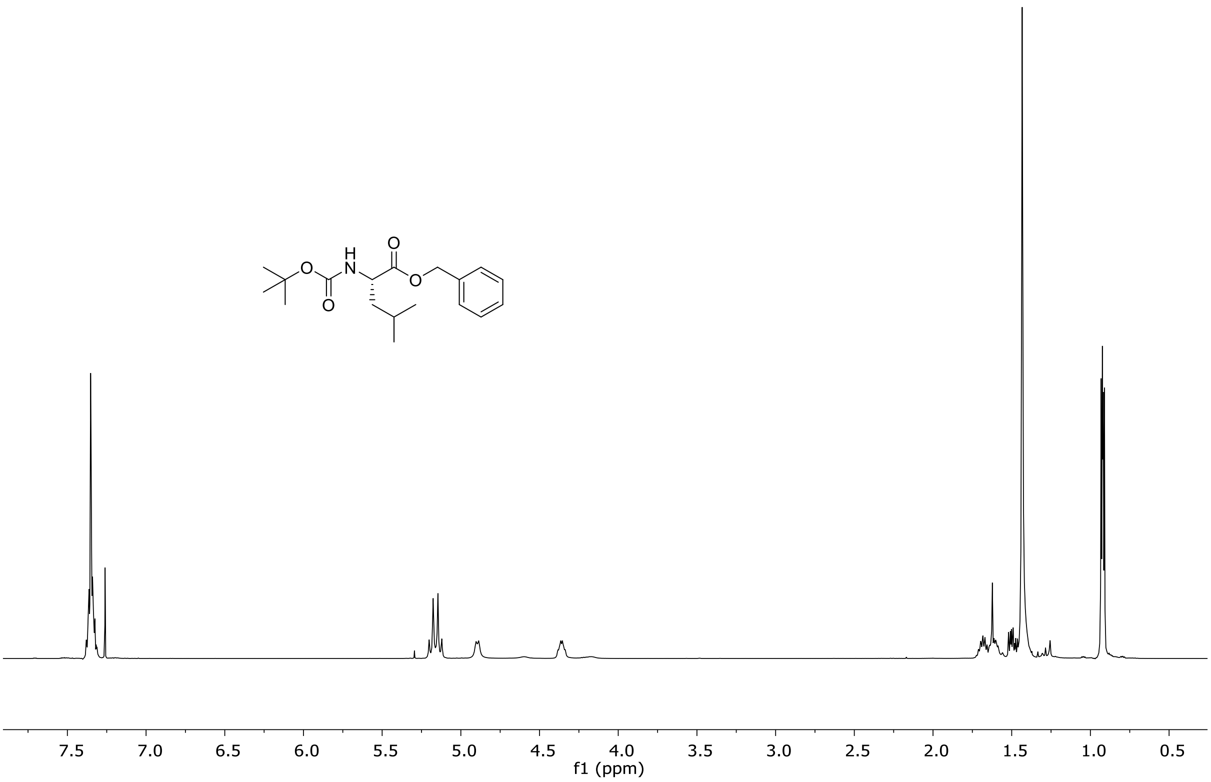

Spectrum S1. ^{1}H-NMR (500 MHz, $CDCl_3$, 298 K) of **S6**.

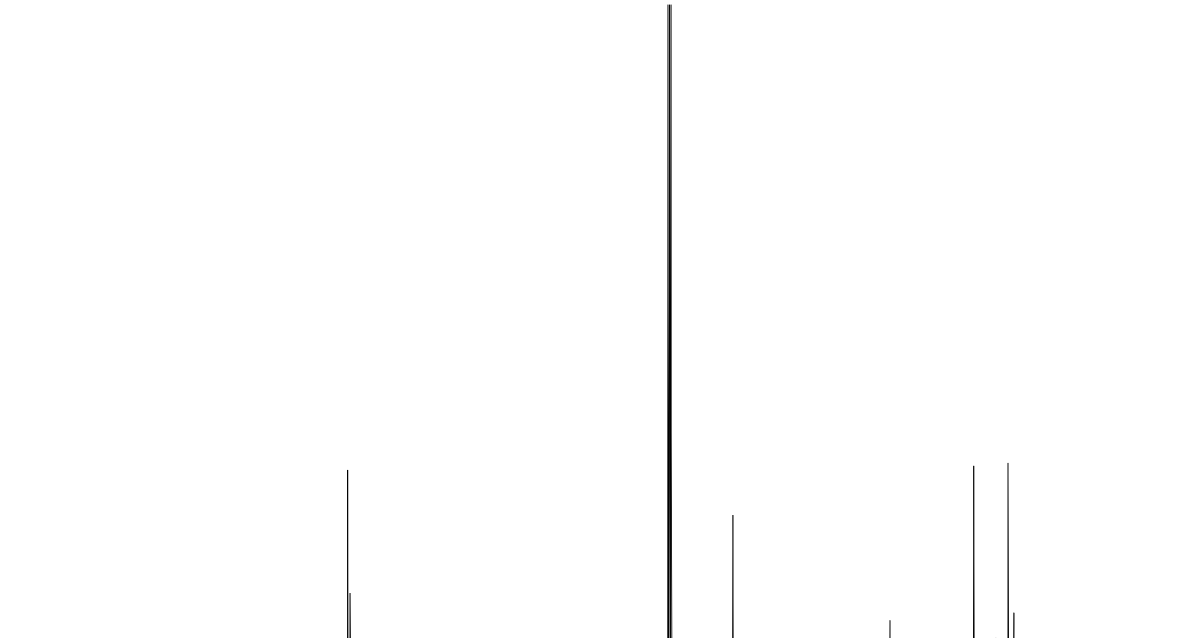

Spectrum S2. ^{13}C-NMR (125 MHz, $CDCl_3$, 298 K) of **S6**.

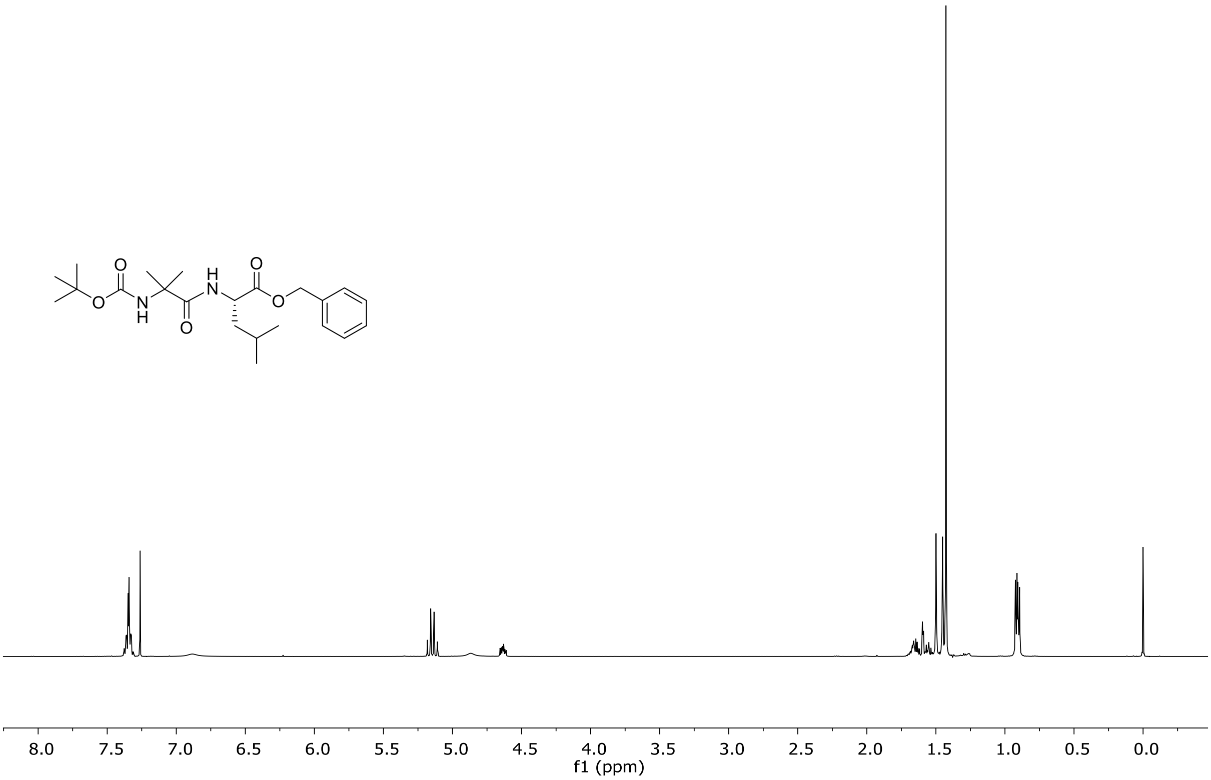

Spectrum S3. ^{1}H-NMR (500 MHz, $CDCl_3$, 298 K) of **S8**.

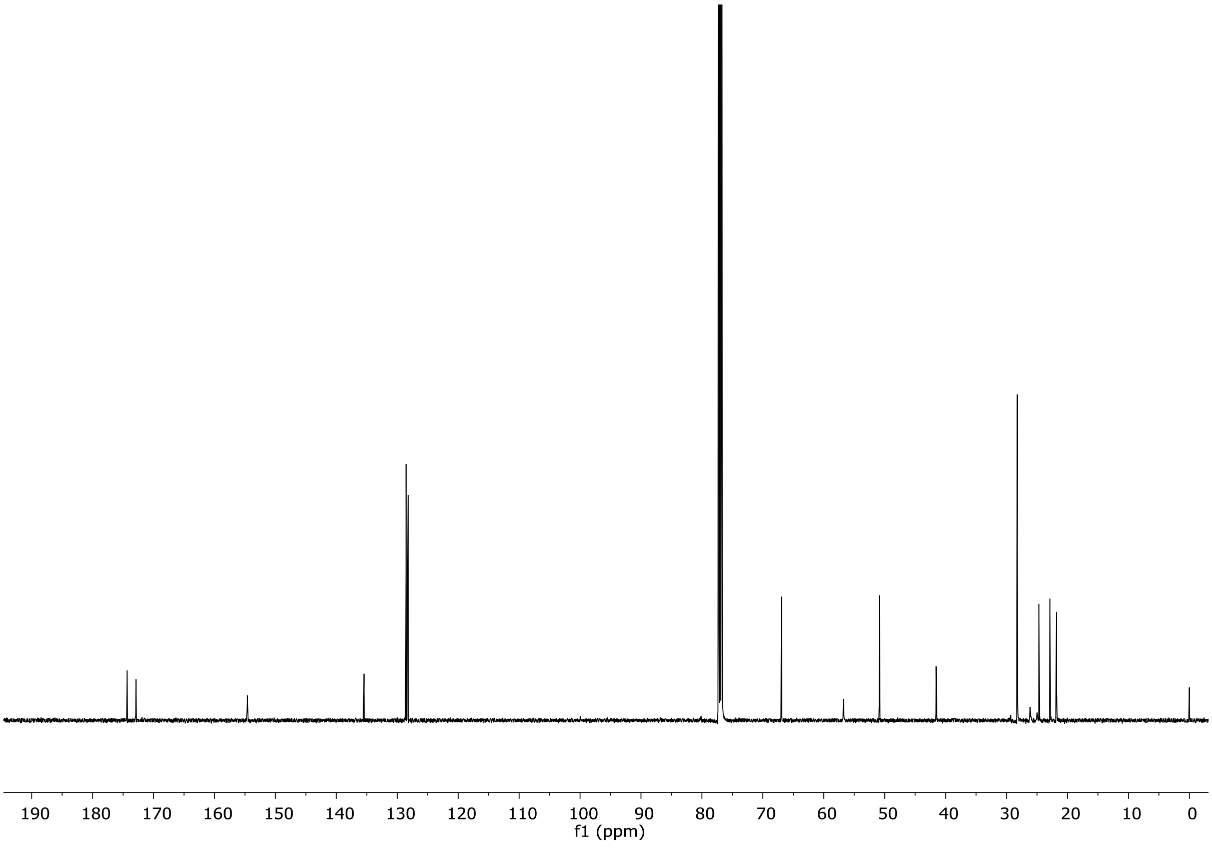

Spectrum S4. ^{13}C-NMR (125 MHz, $CDCl_3$, 298 K) of **S8**.

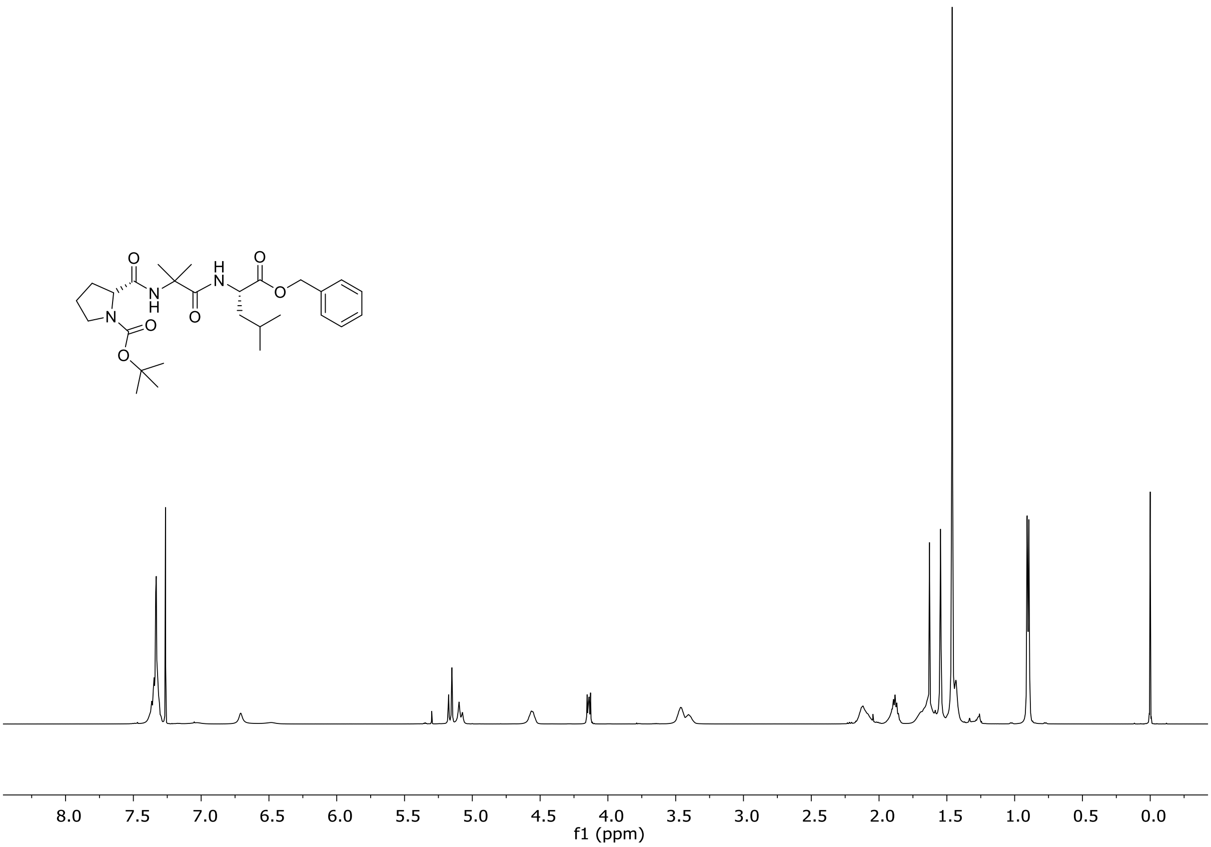

Spectrum S5. ^{1}H-NMR (500 MHz, $CDCl_3$, 298 K) of **S10**.

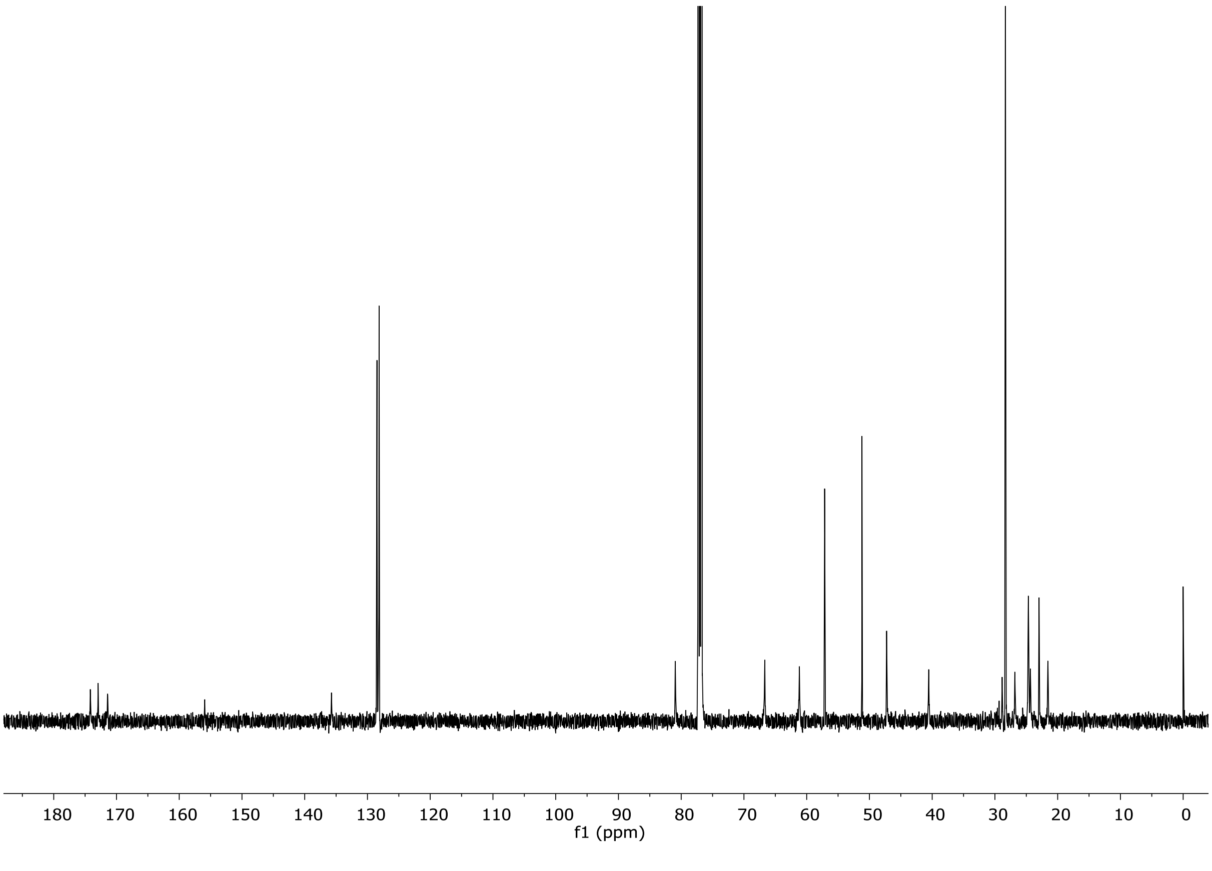

Spectrum S6. ^{13}C-NMR (125 MHz, $CDCl_3$, 298 K) of **S10**.

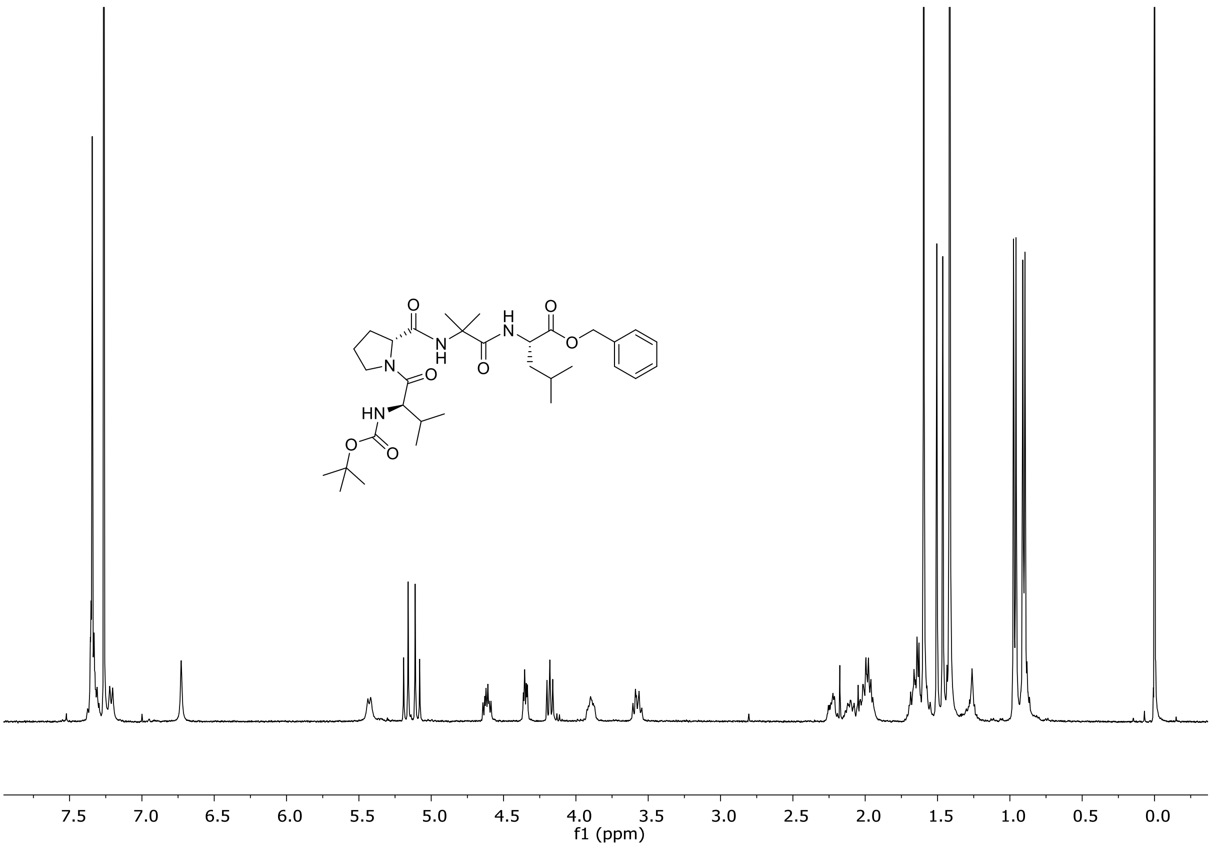

Spectrum S7. ^{1}H-NMR (500 MHz, $CDCl_3$, 298 K) of **S12**.

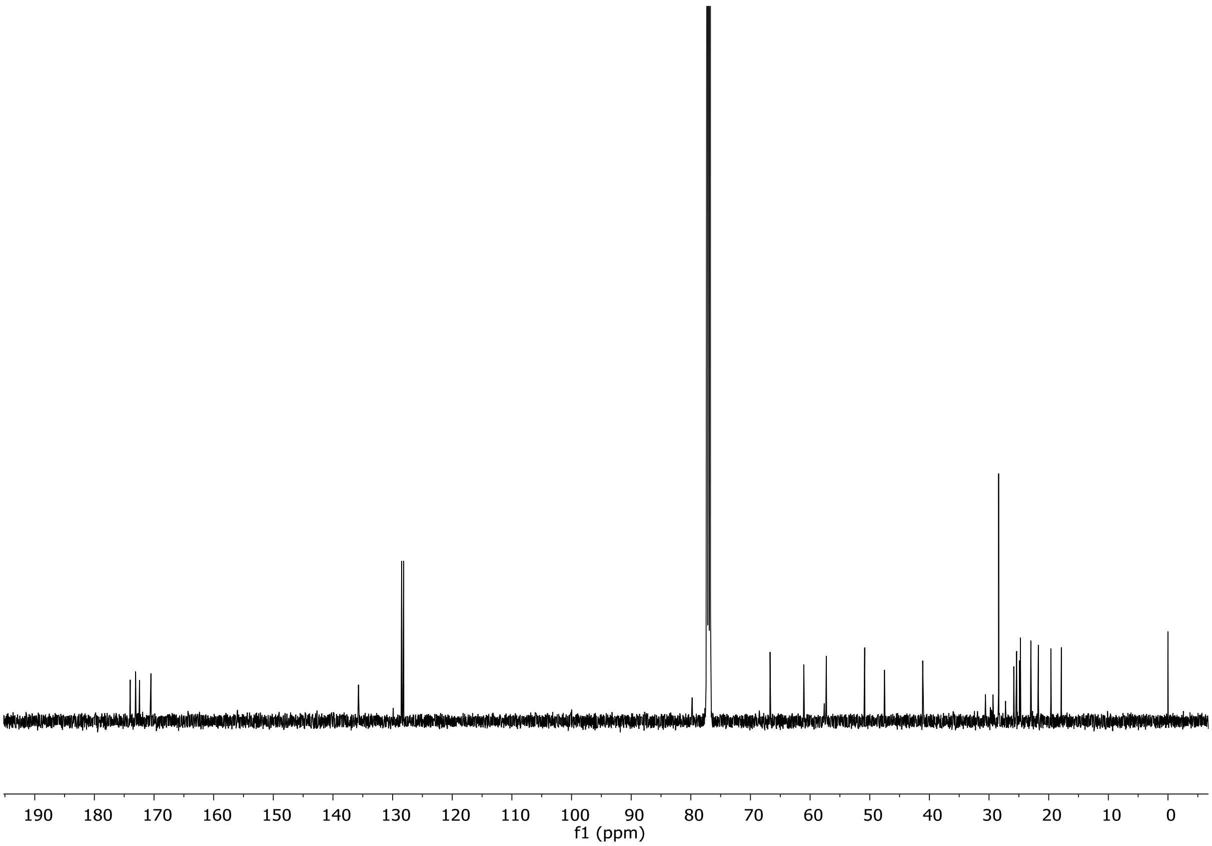

Spectrum S8. ^{13}C-NMR (125 MHz, $CDCl_3$, 298 K) of **S12**.

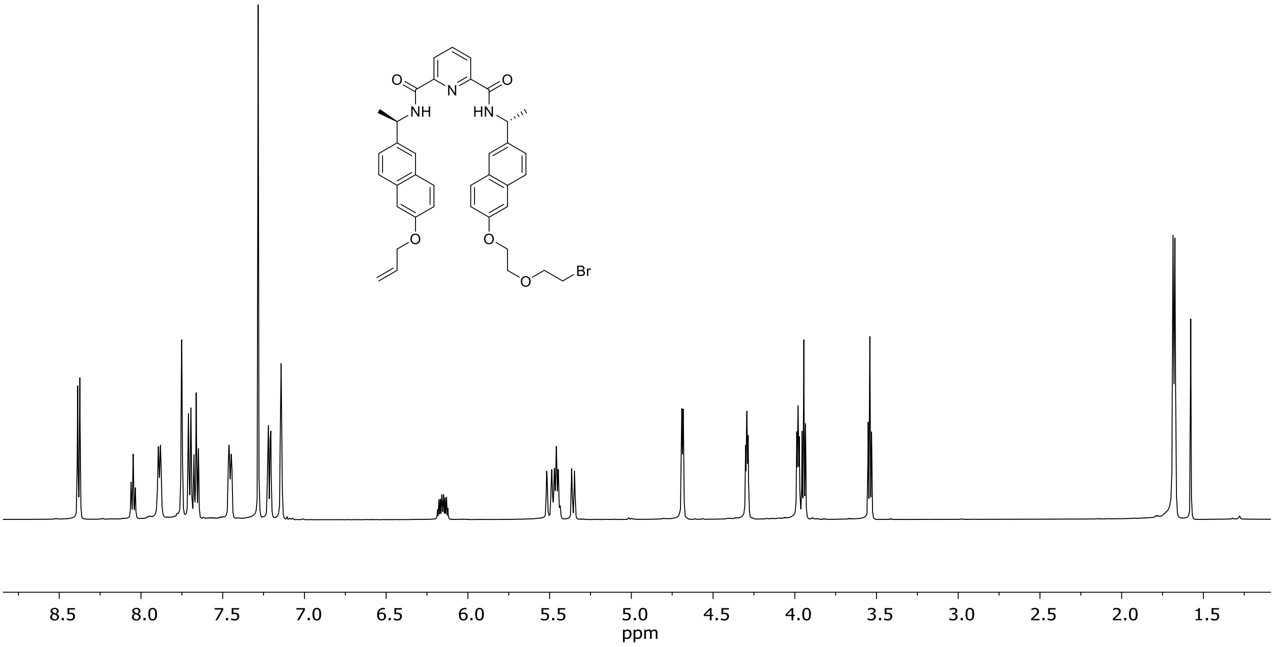

Spectrum S9. ^{1}H-NMR (600 MHz, $CDCl_3$, 298 K) of **S16**.

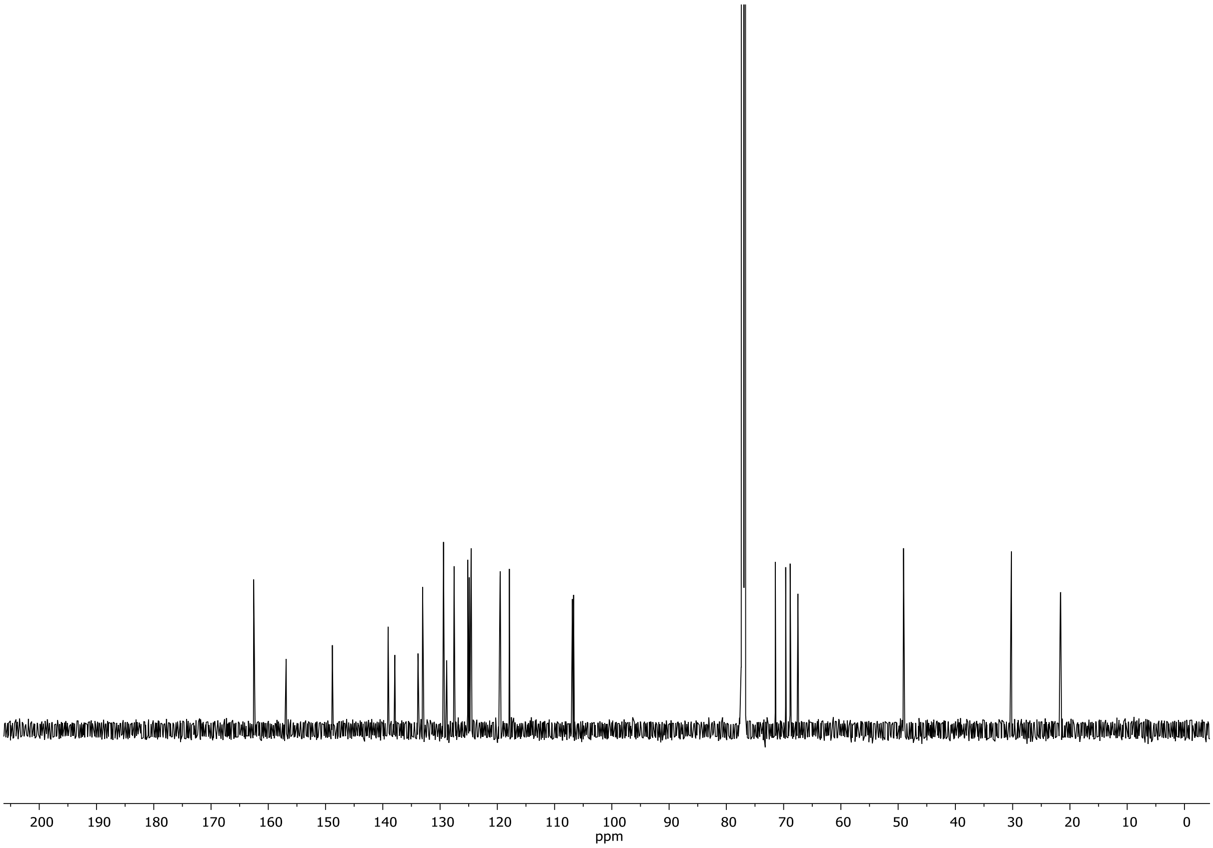

Spectrum S10. ^{13}C-NMR (151 MHz, $CDCl_3$, 298 K) of **S16.**

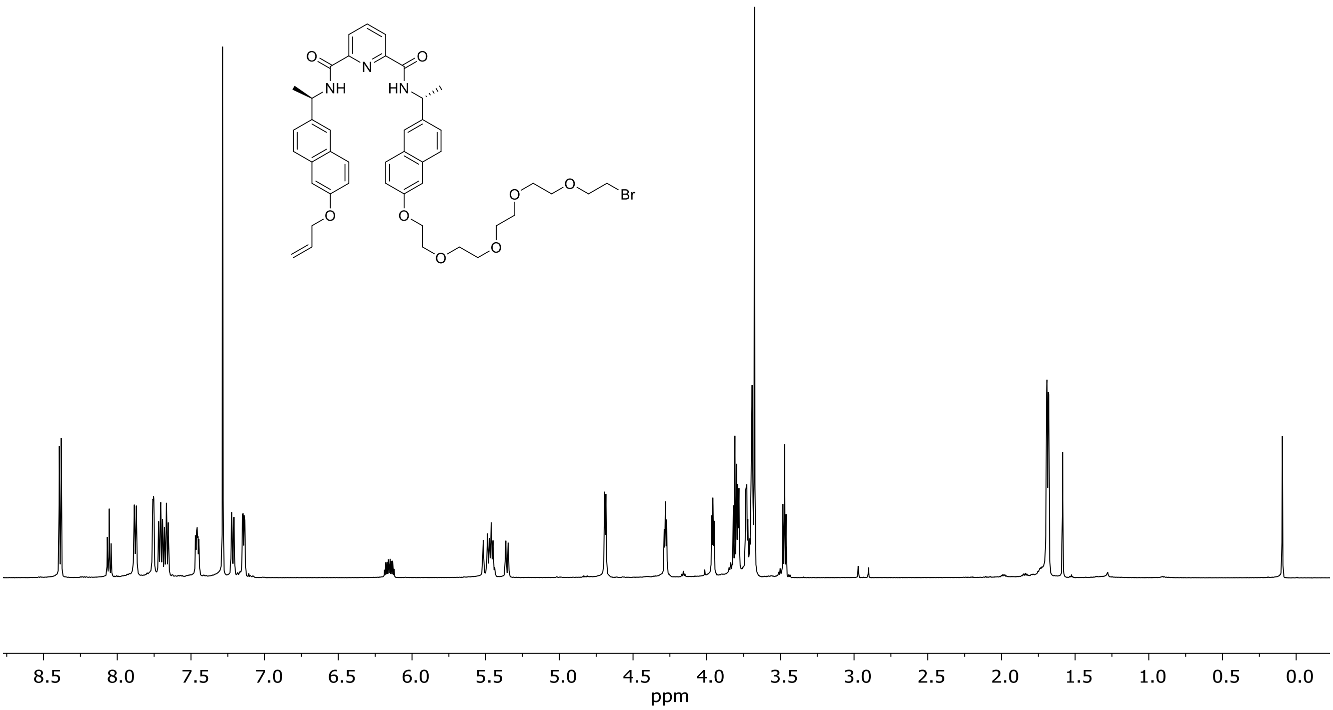

Spectrum S11. ^{1}H-NMR (600 MHz, $CDCl_3$, 298 K) of **S19**.

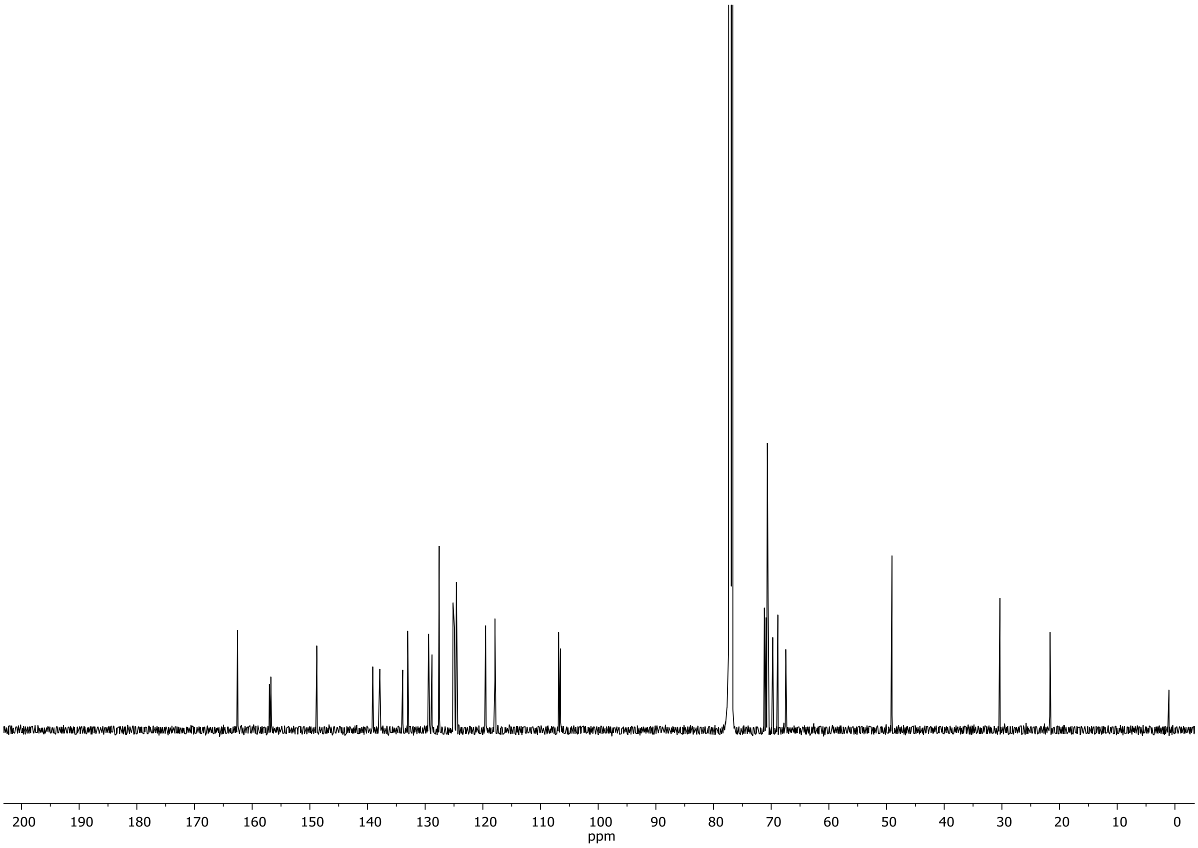

Spectrum S12. ^{13}C-NMR (151 MHz, $CDCl_3$, 298 K) of **S19.**

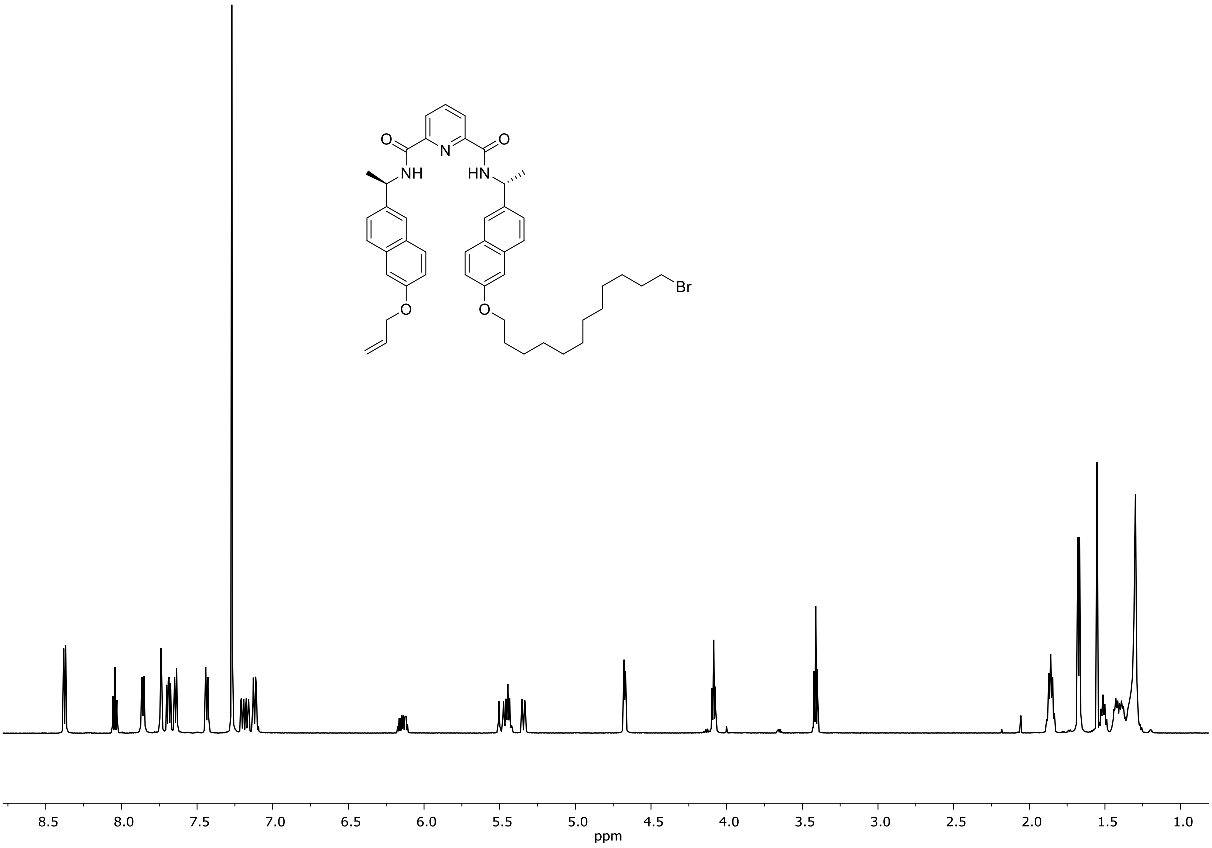

Spectrum S13. ^{1}H-NMR (600 MHz, $CDCl_3$, 298 K) of **S21**.

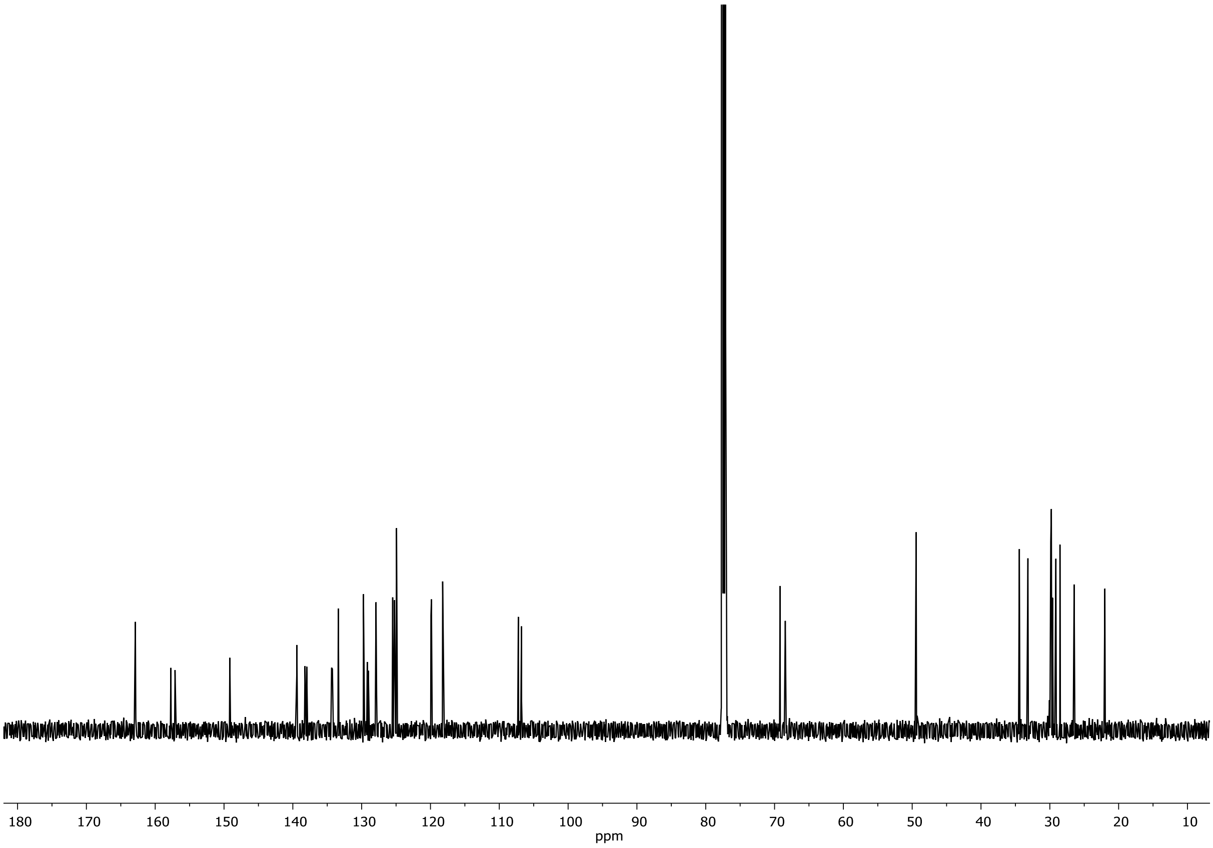

Spectrum S14. ^{13}C-NMR (151 MHz, $CDCl_3$, 298 K) of **S21.**

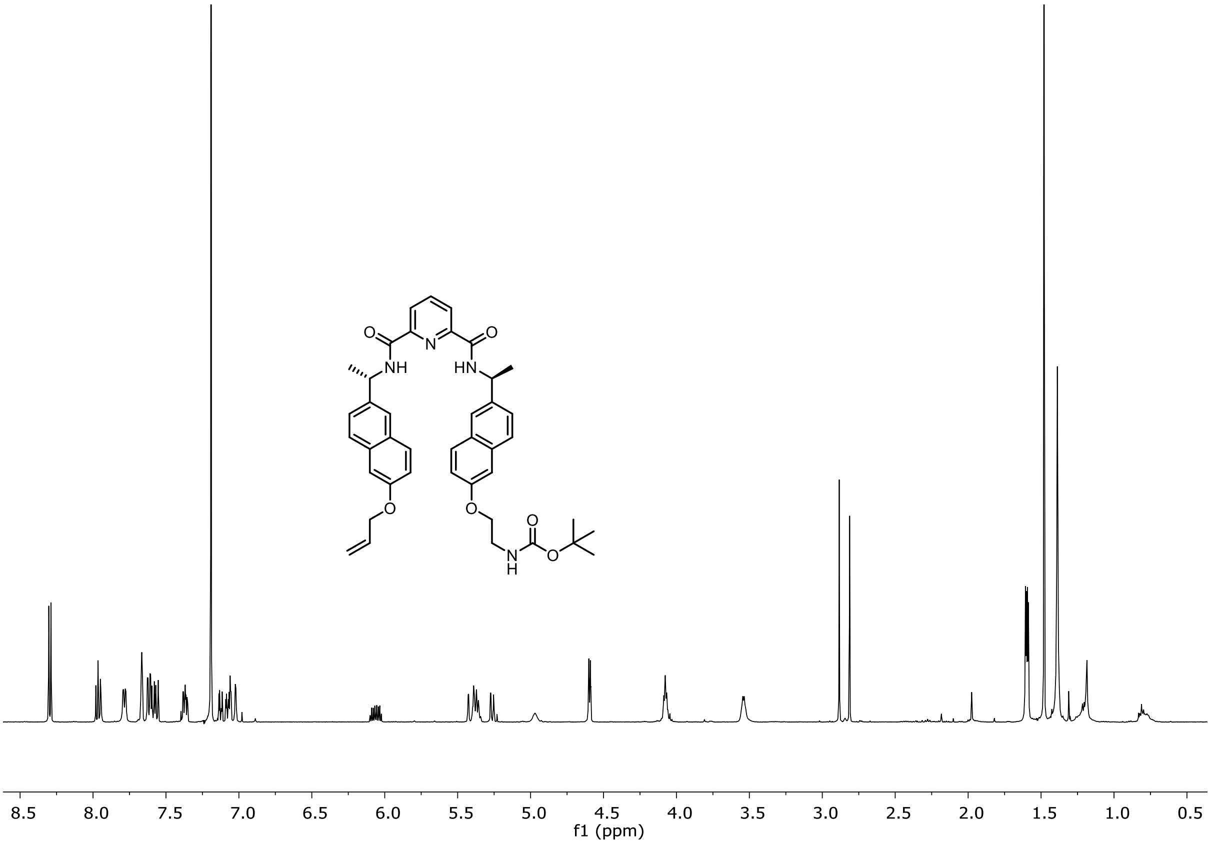

Spectrum S15. ^{1}H-NMR (500 MHz, $CDCl_3$, 298 K) of **S22**.

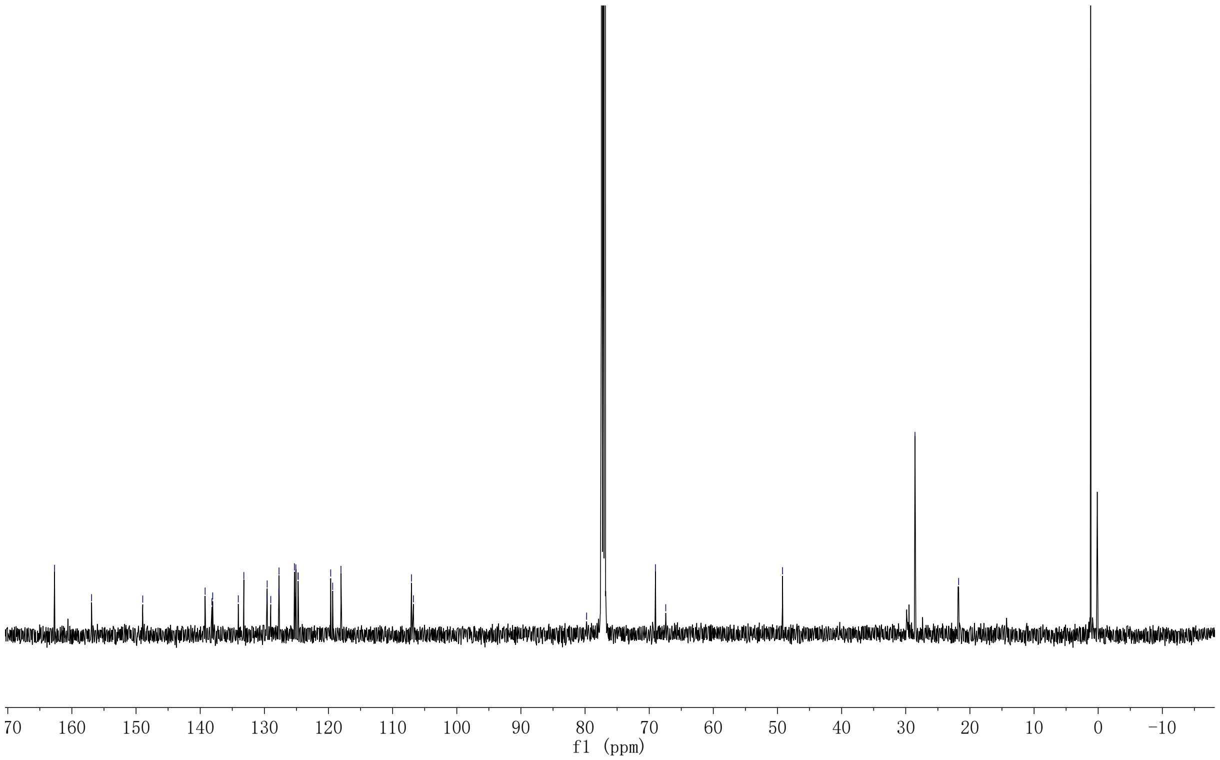

Spectrum S16. ^{13}C-NMR (125 MHz, $CDCl_3$, 298 K) of **S22**.

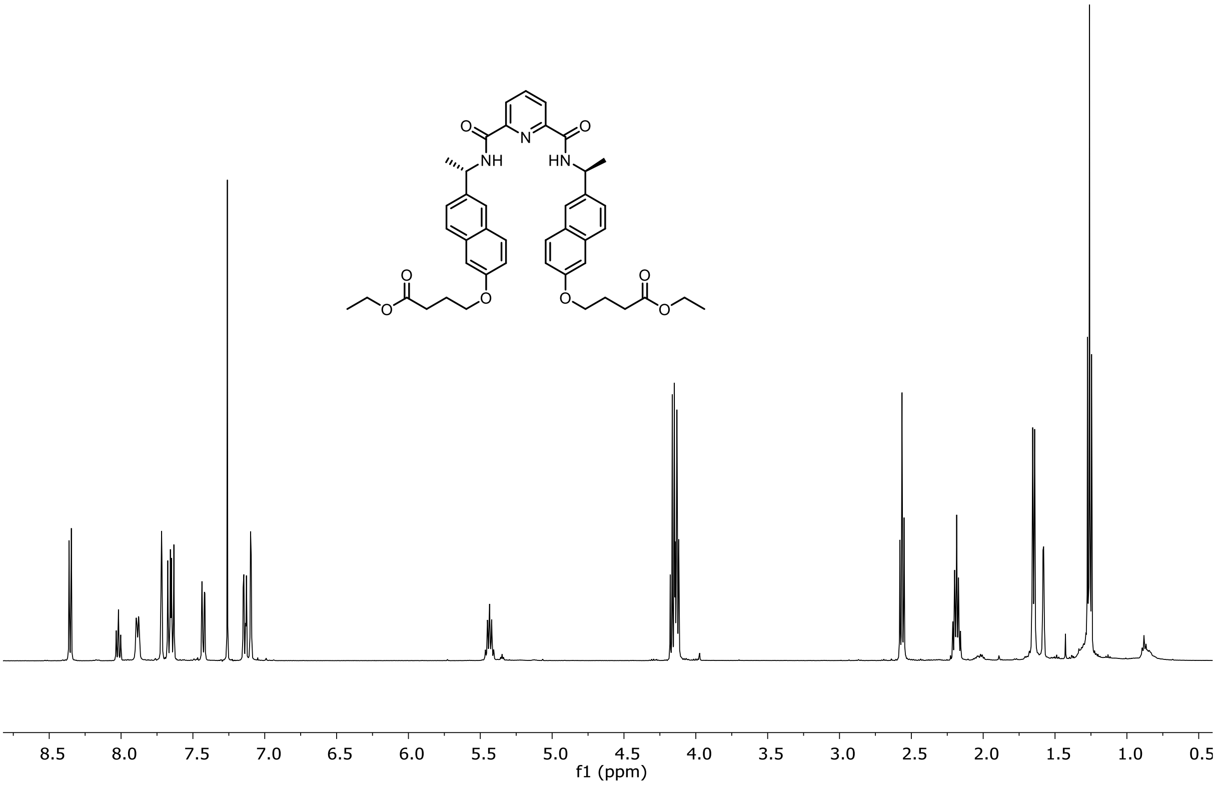

Spectrum S17. ^{1}H-NMR (500 MHz, $CDCl_3$, 298 K) of **S23**.

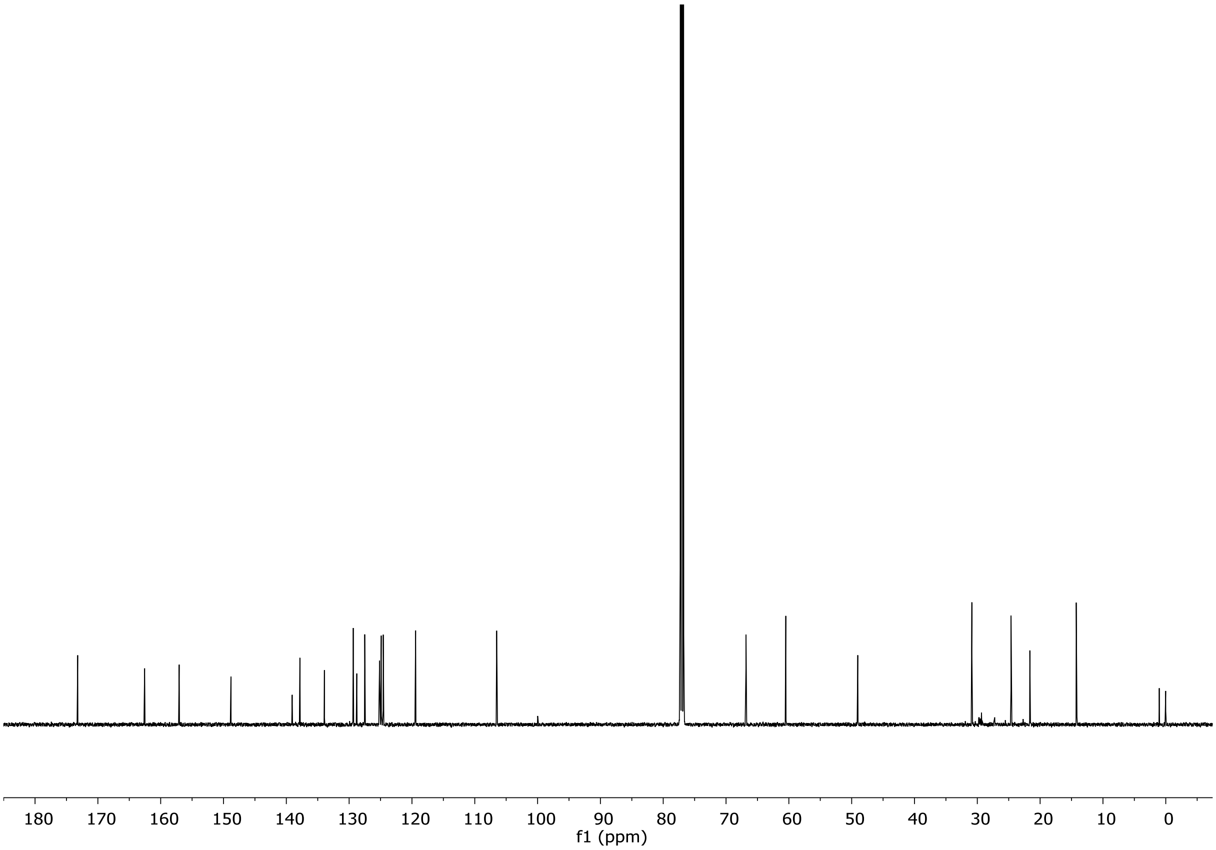

Spectrum S18. ^{13}C-NMR (125 MHz, $CDCl_3$, 298 K) of **S23**.

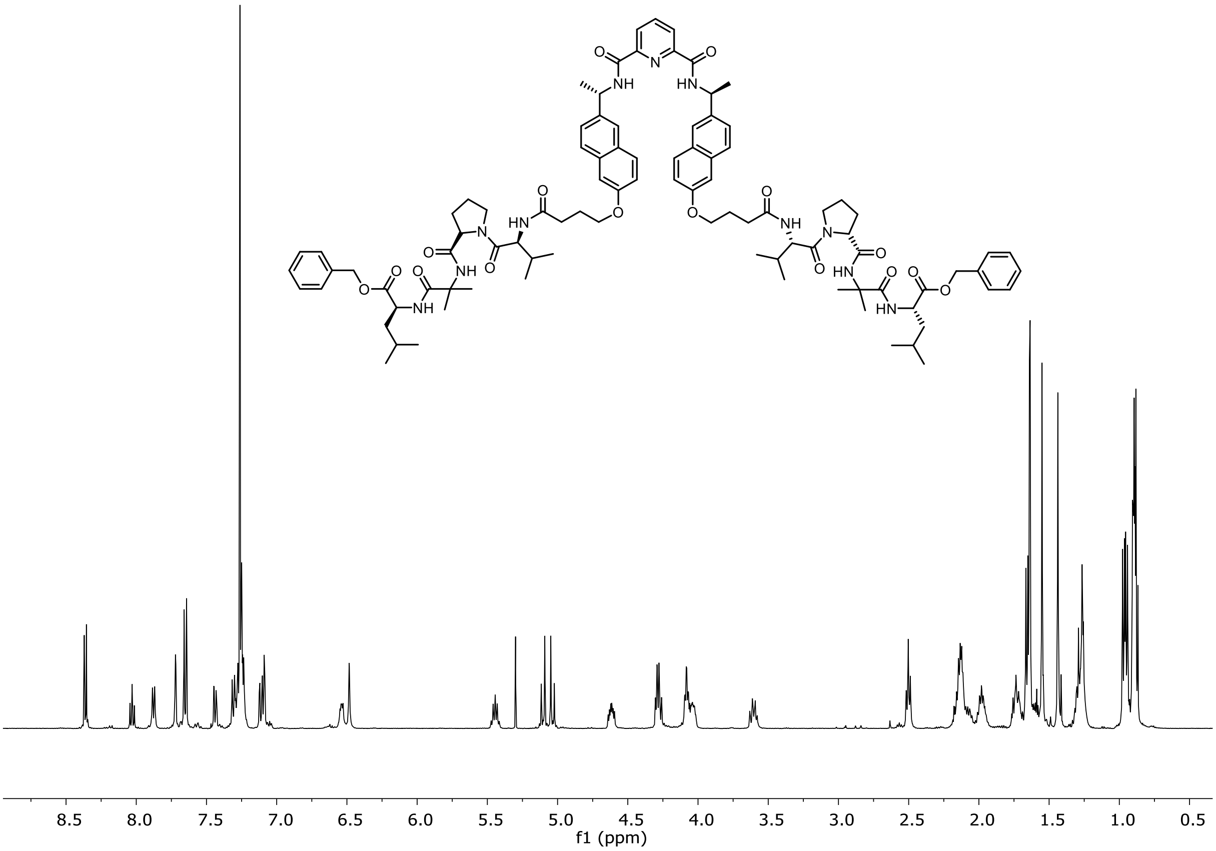

Spectrum S19. ^{1}H-NMR (500 MHz, $CDCl_3$, 298 K) of **A5**.

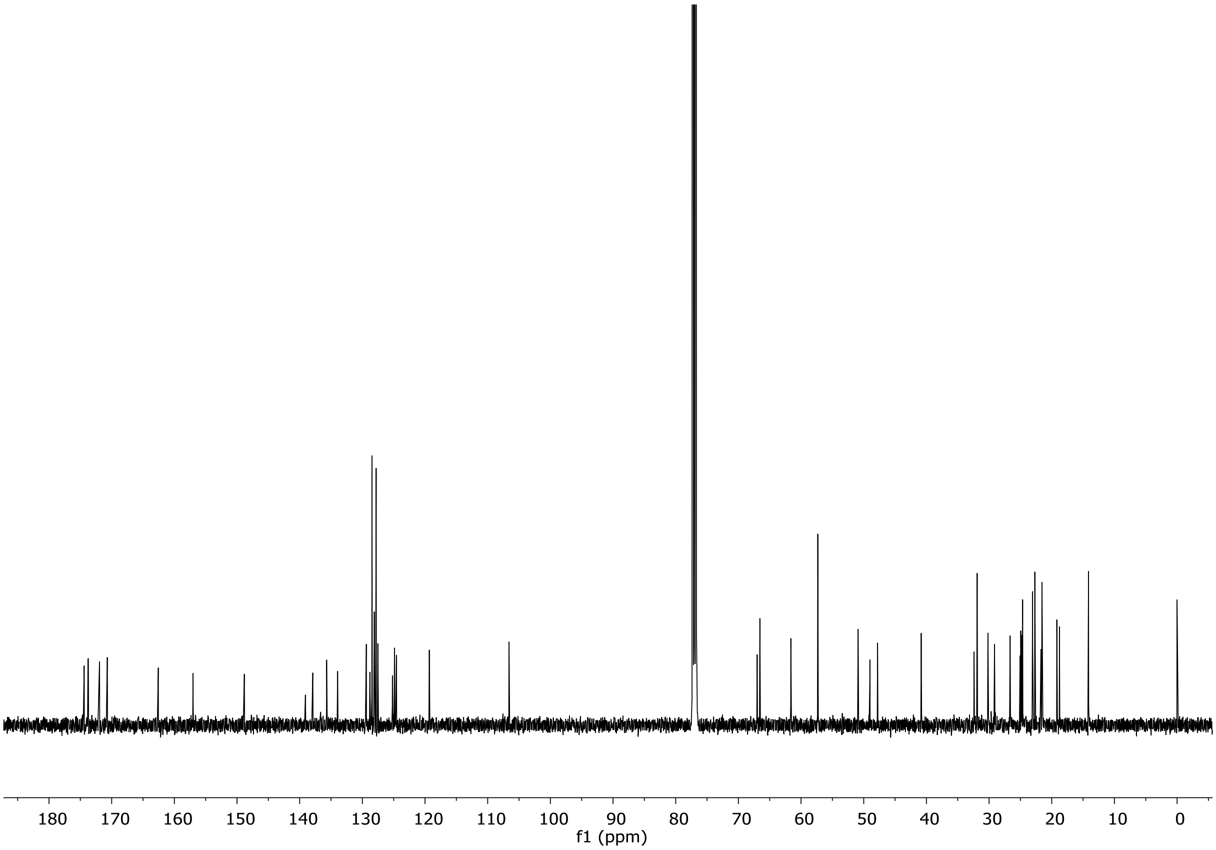

Spectrum S20. ^{13}C-NMR (125 MHz, $CDCl_3$, 298 K) of **A5**.

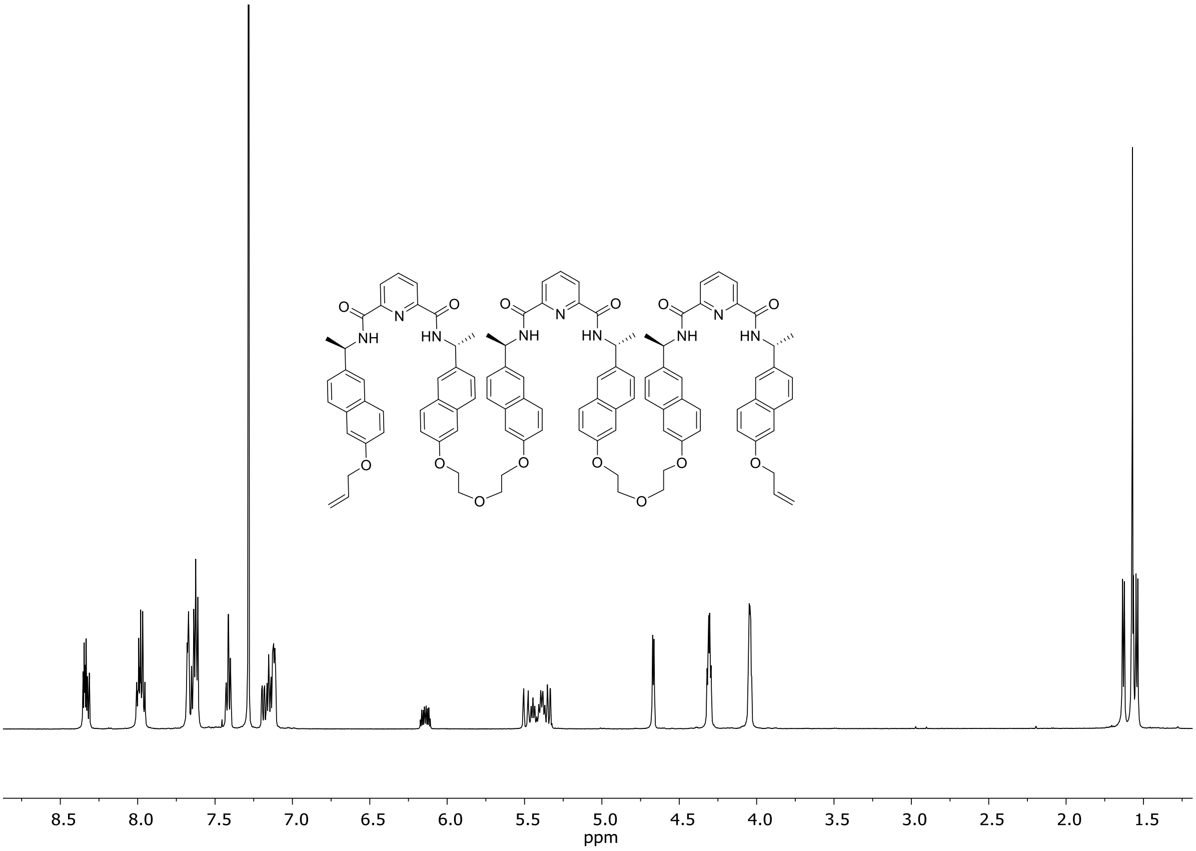

Spectrum S21. ^{1}H-NMR (600 MHz, $CDCl_3$, 298 K) of **1**.

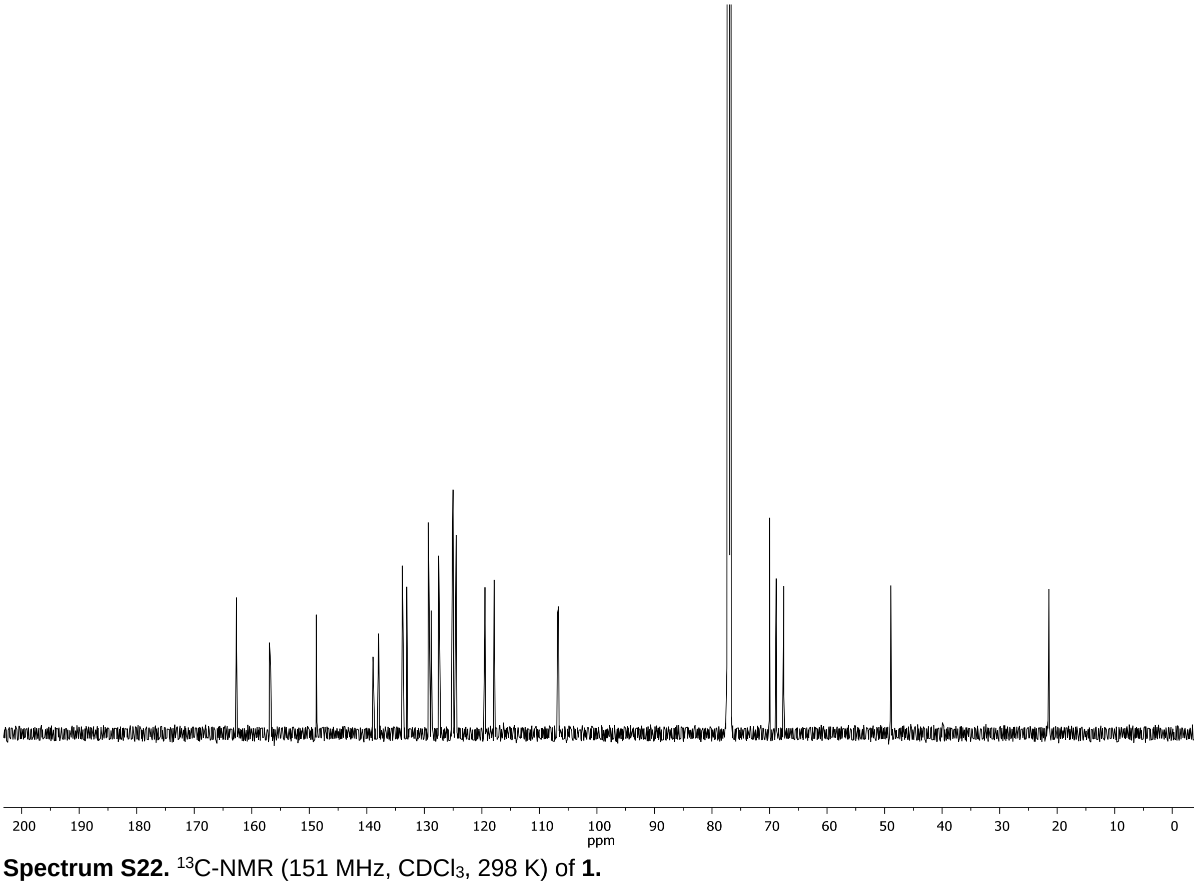

Spectrum S22. ^{13}C-NMR (151 MHz, $CDCl_3$, 298 K) of **1.**

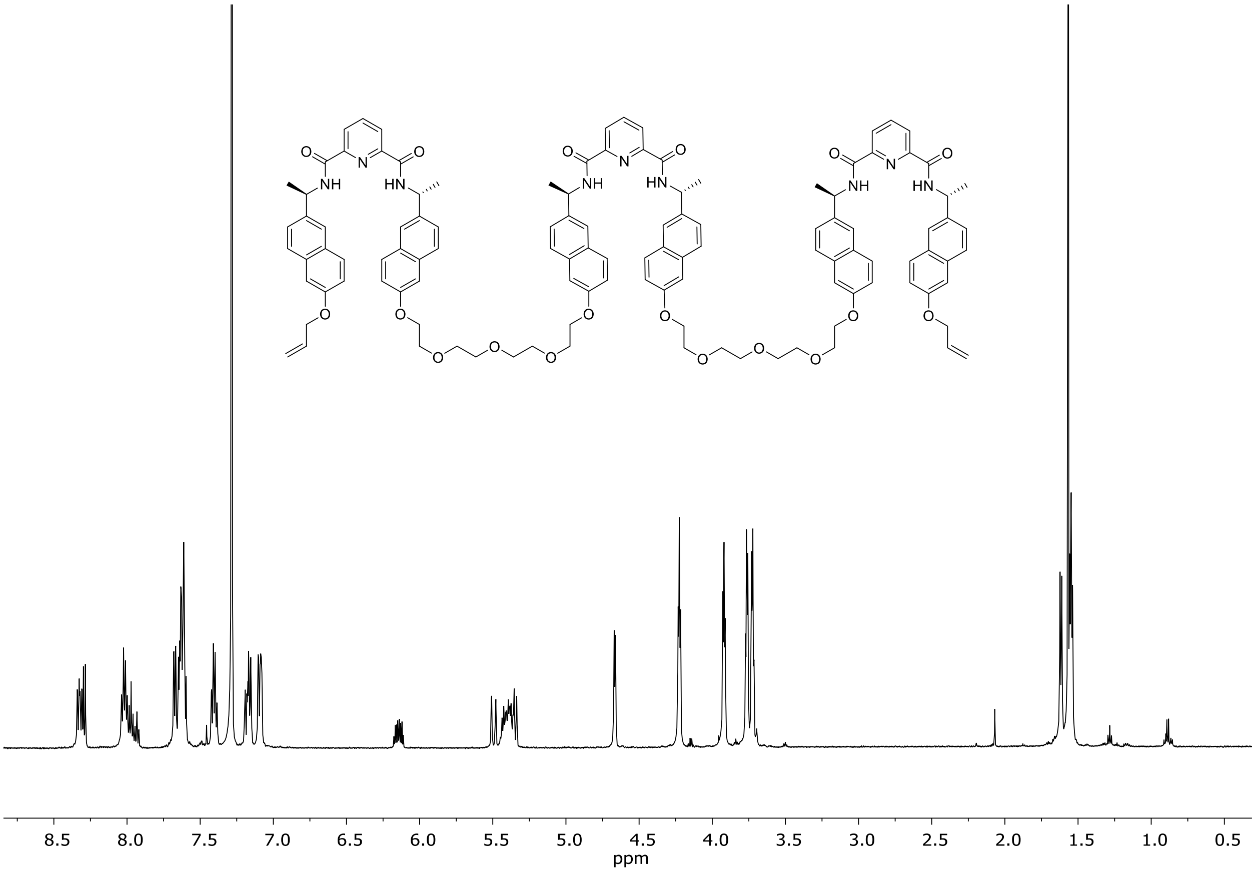

Spectrum S23. ^{1}H-NMR (600 MHz, $CDCl_3$, 298 K) of **3**.

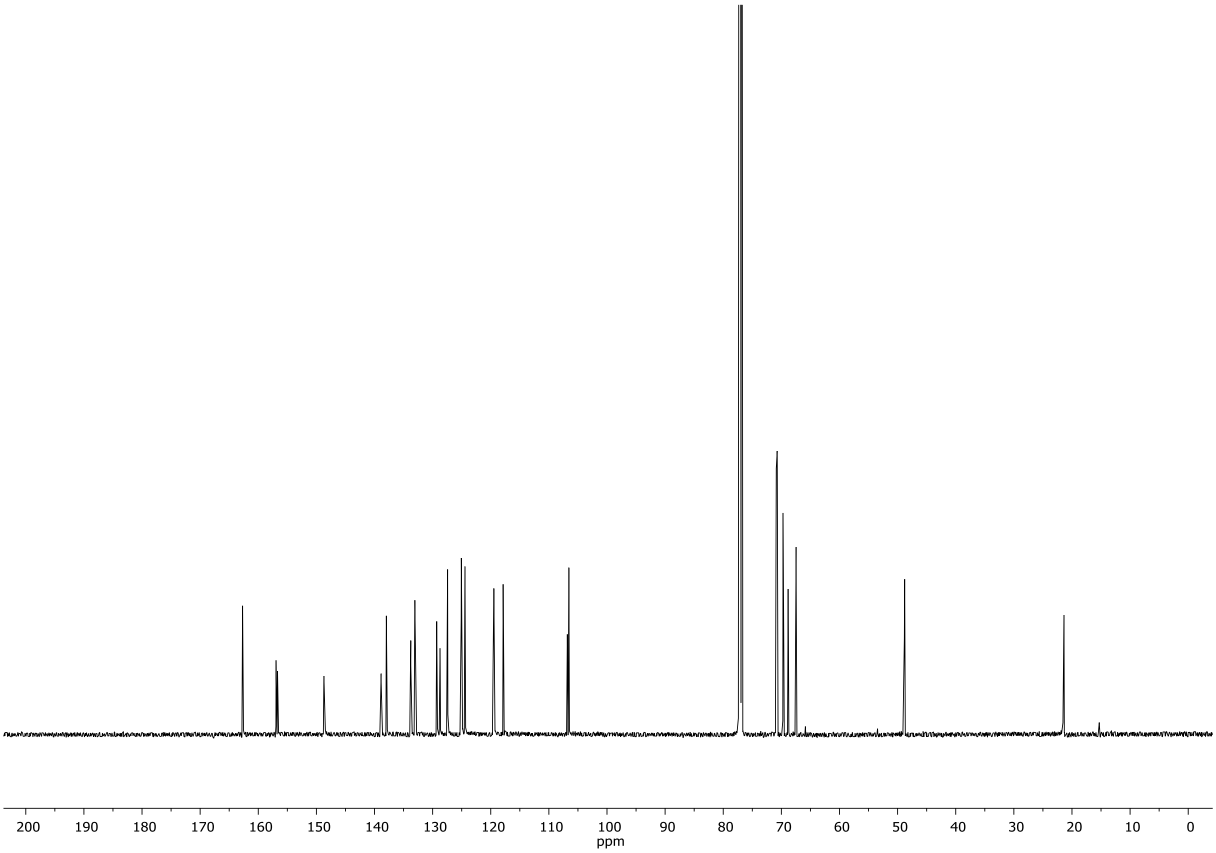

Spectrum S24. ^{13}C-NMR (151 MHz, $CDCl_3$, 298 K) of **3.**

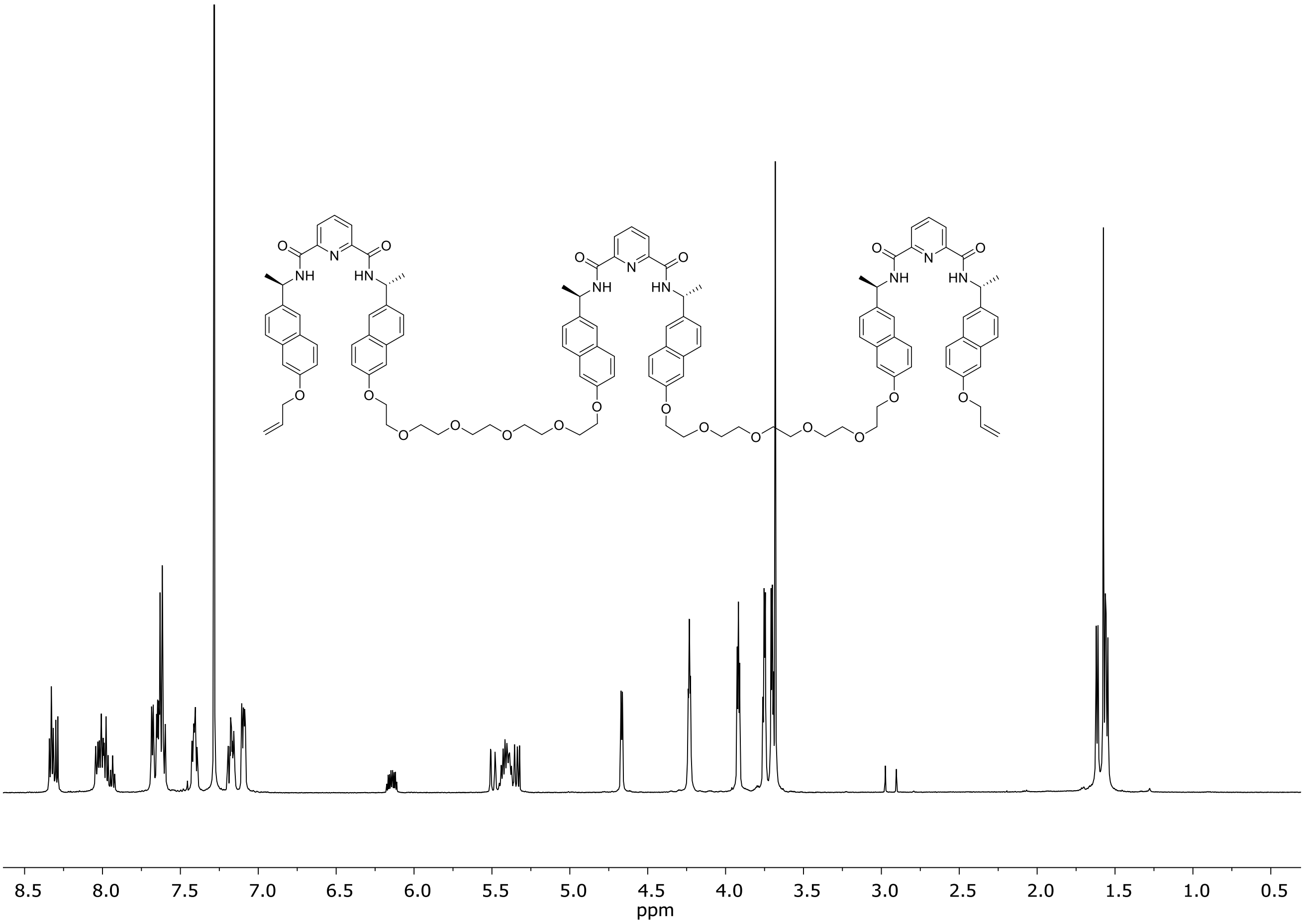

Spectrum S25. ^{1}H-NMR (600 MHz, $CDCl_3$, 298 K) of **4**.

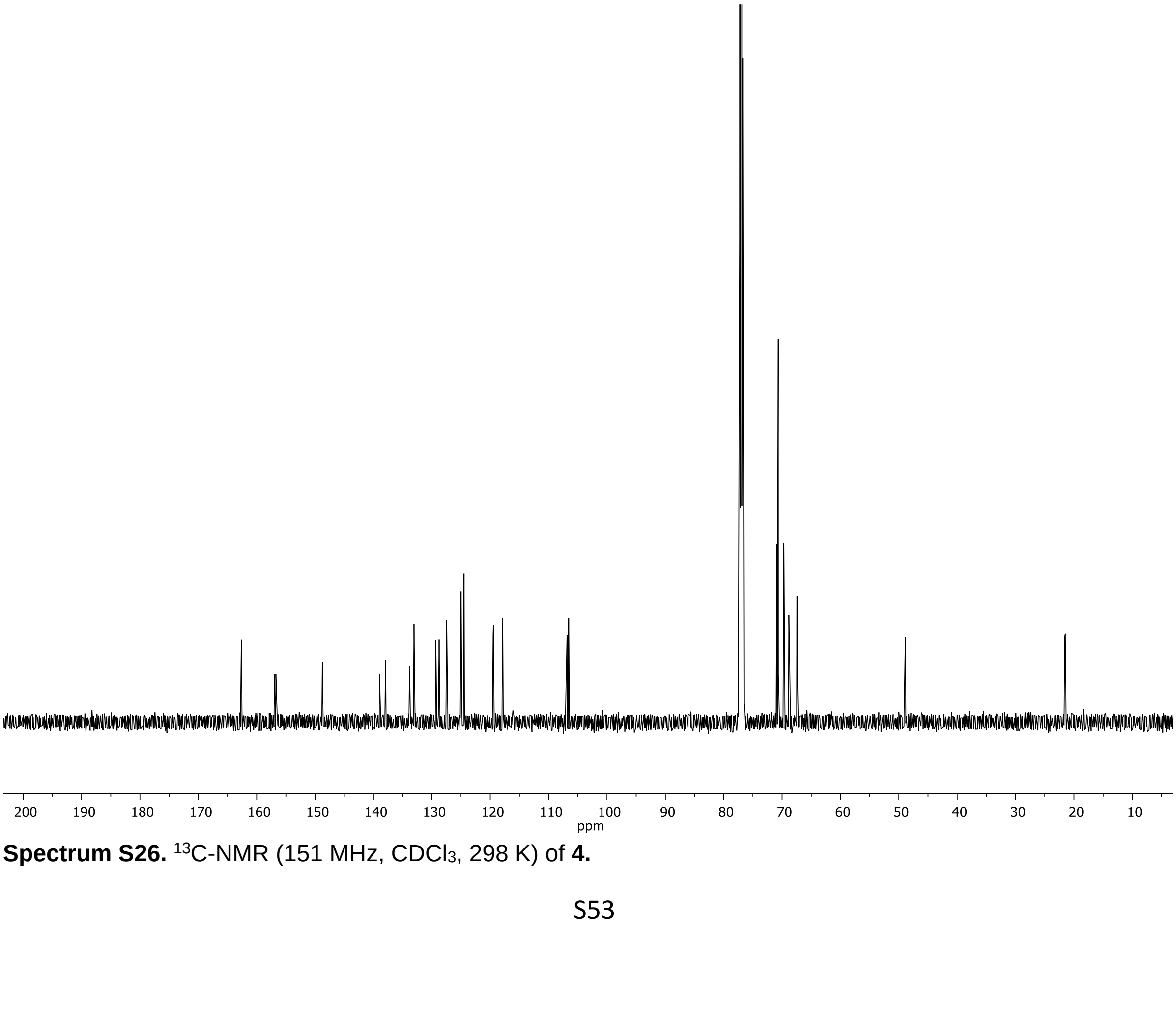

Spectrum S26. ^{13}C-NMR (151 MHz, $CDCl_3$, 298 K) of **4.**

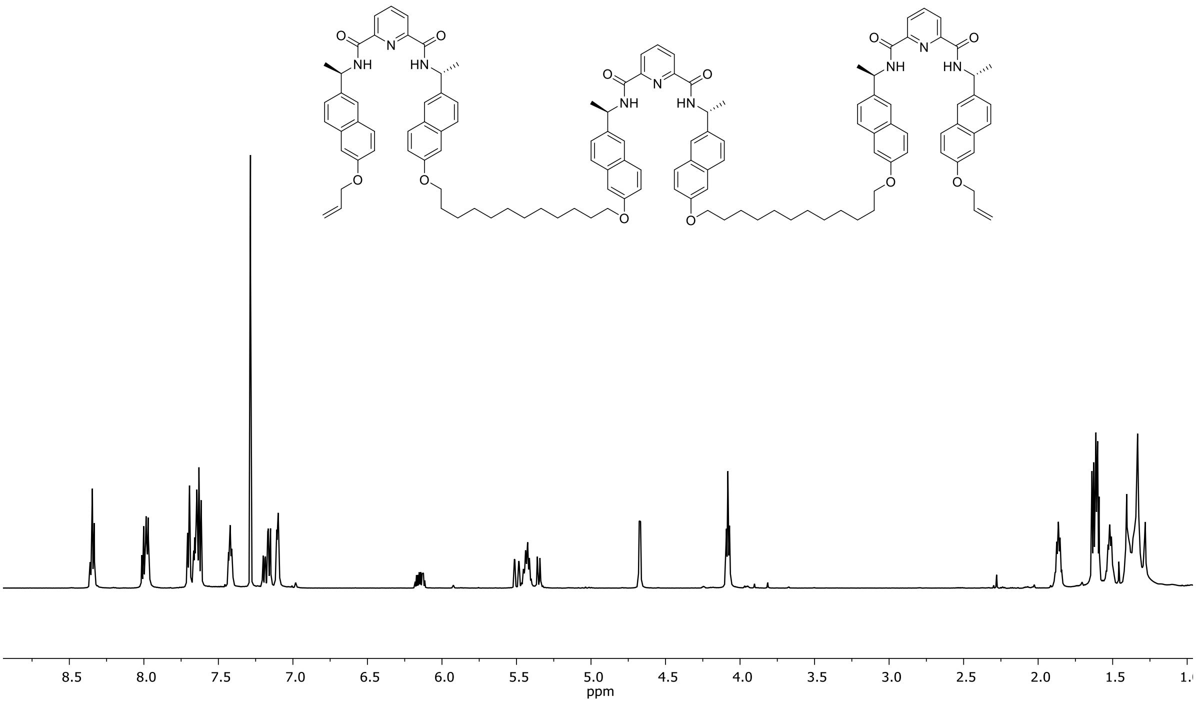

Spectrum S27. ^{1}H-NMR (600 MHz, $CDCl_3$, 298 K) of **6**.

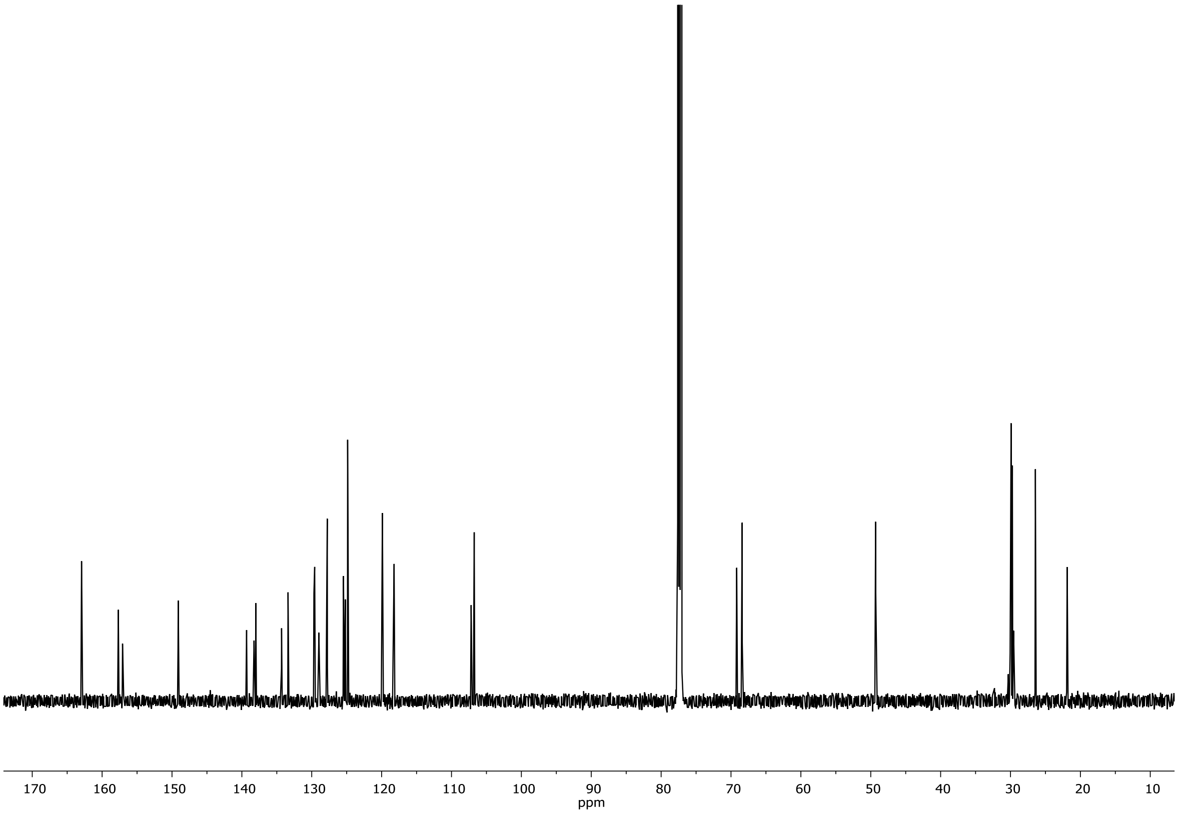

Spectrum S28. ^{13}C-NMR (151 MHz, $CDCl_3$, 298 K) of **6.**

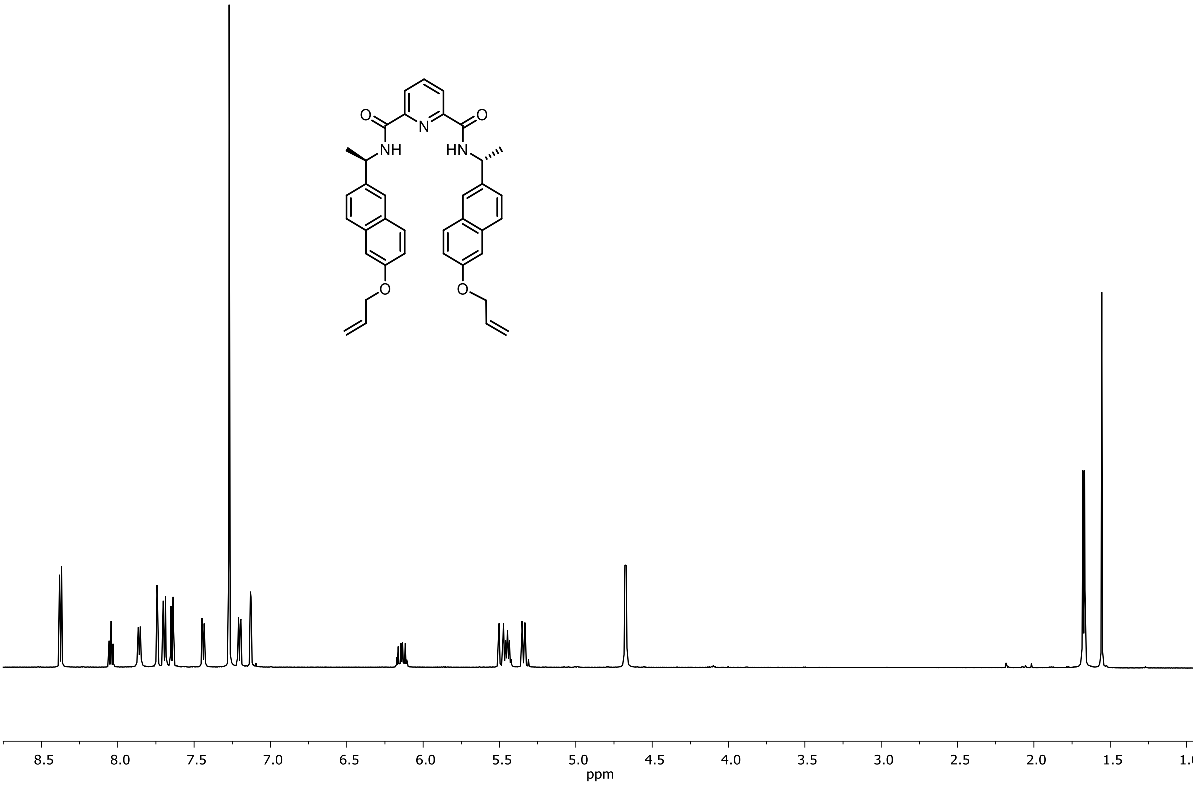

Spectrum S29. ^{1}H-NMR (600 MHz, $CDCl_3$, 298 K) of **7**.

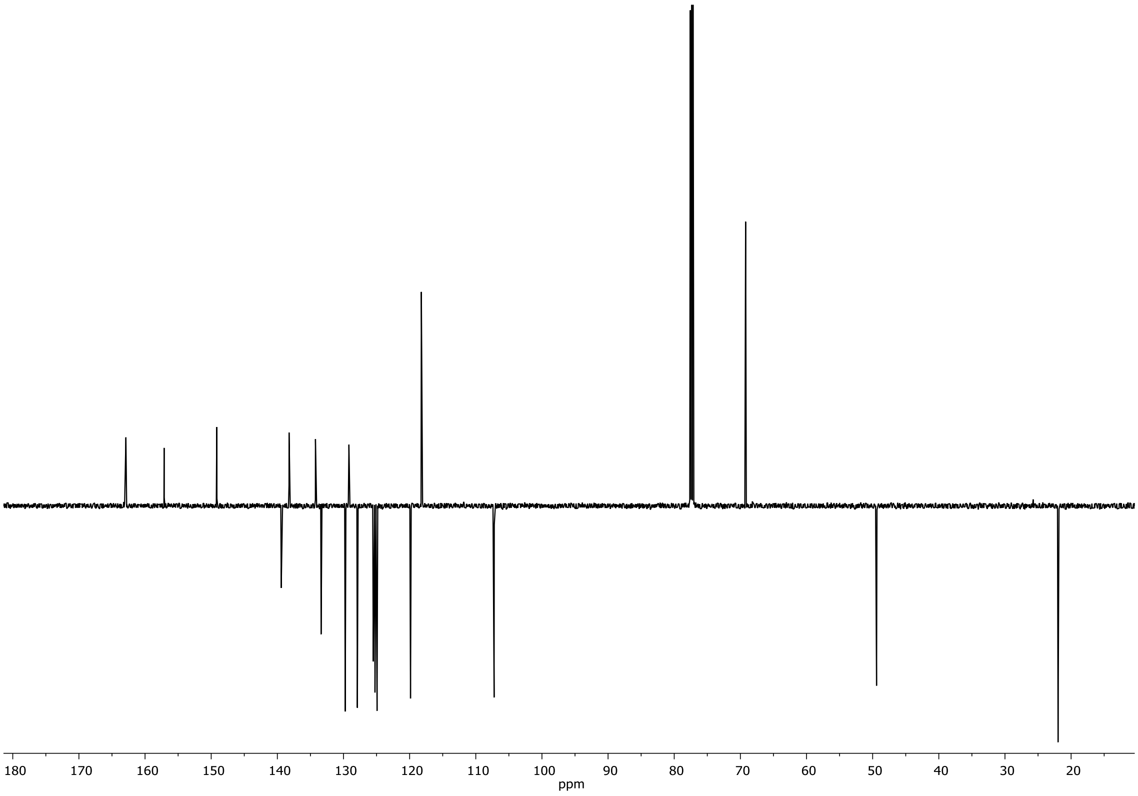

Spectrum S30. ^{13}C DEPT-NMR (151 MHz, CD_3CN, 298 K) of **7.**

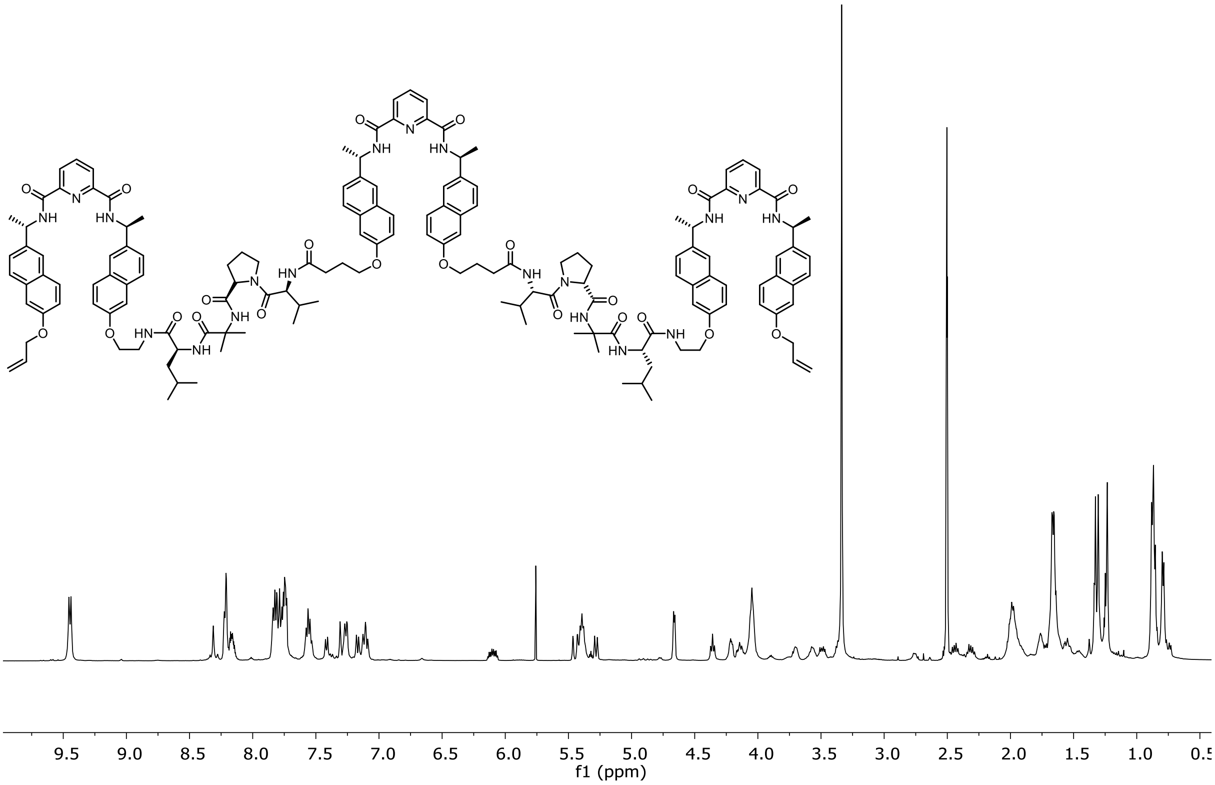

Spectrum S31. ^{1}H-NMR (500 MHz, DMSO-d_6, 298 K) of **8**.

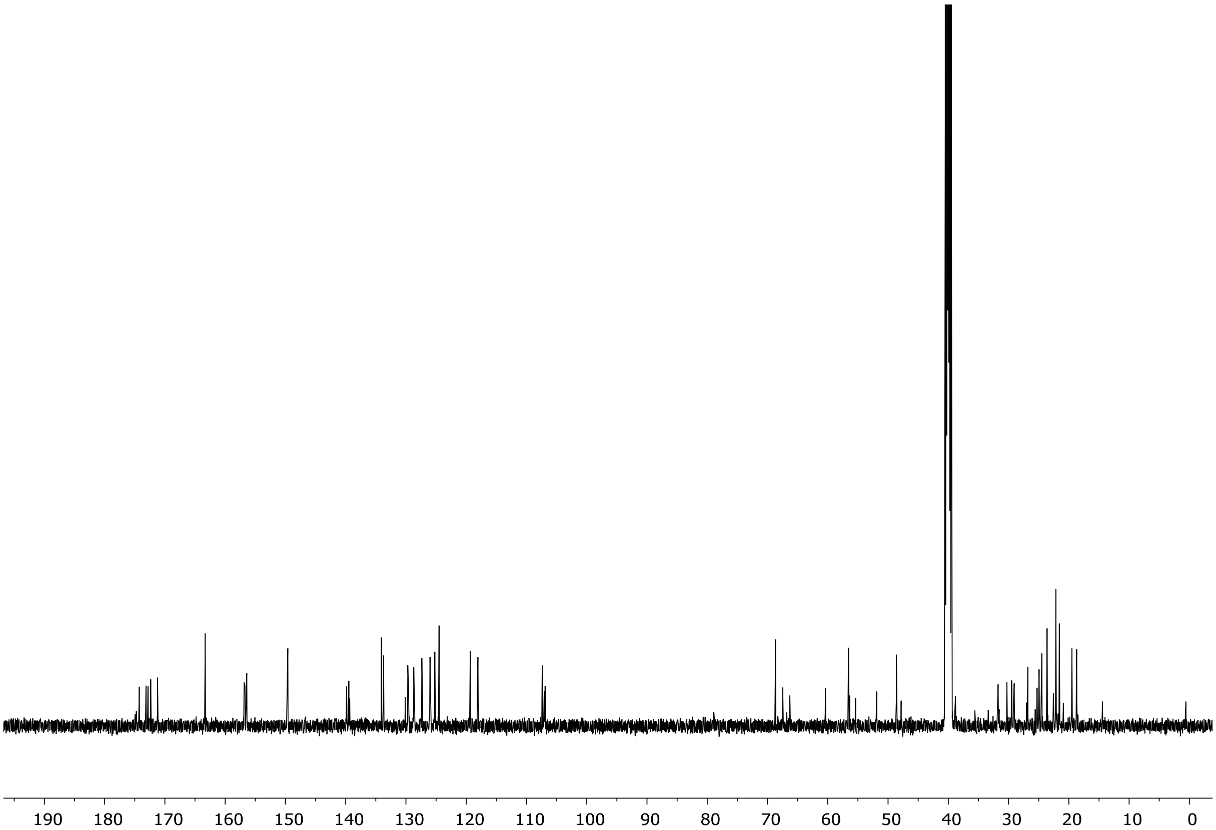

Spectrum S32. ^{13}C-NMR (125 MHz, DMSO-d_6, 298 K) of **8**.

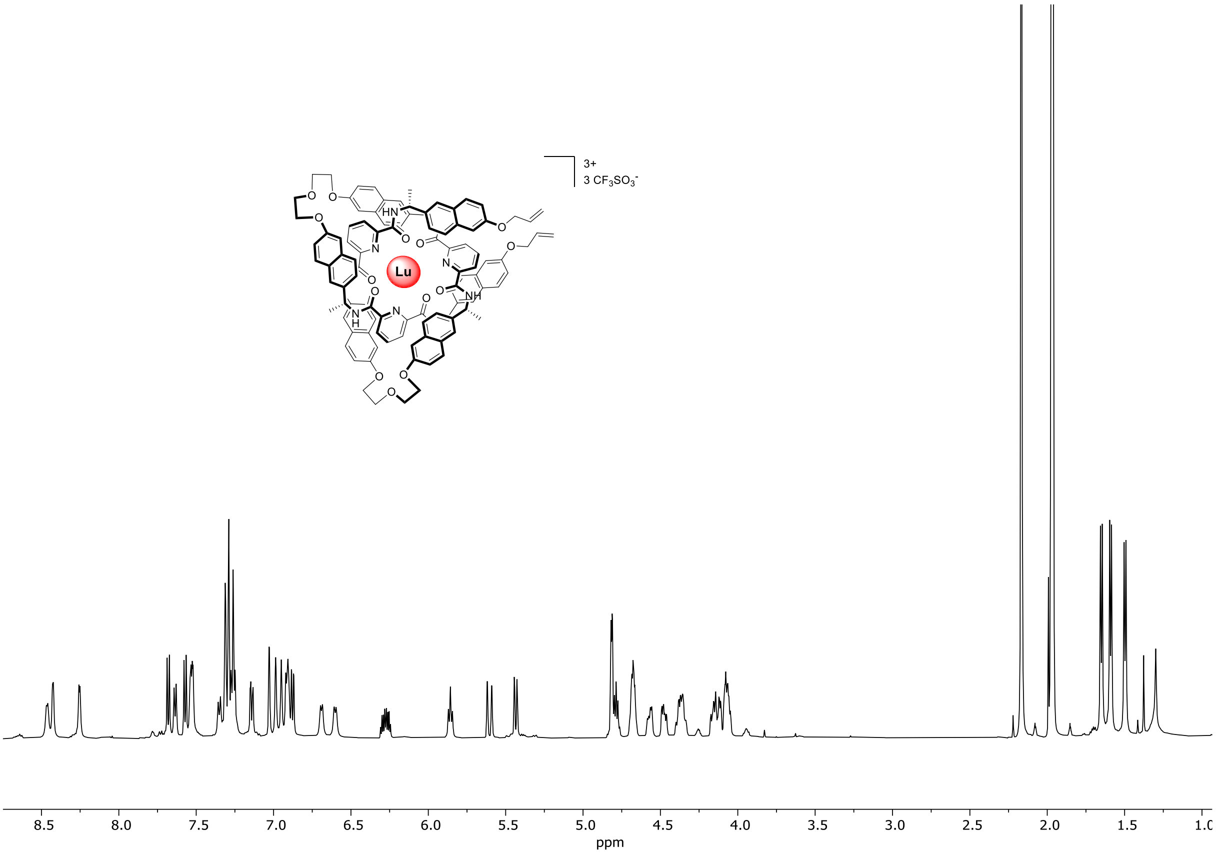

Spectrum S33. ^{1}H-NMR (600 MHz, CD_3CN, 345 K) of **1•**[Lu].

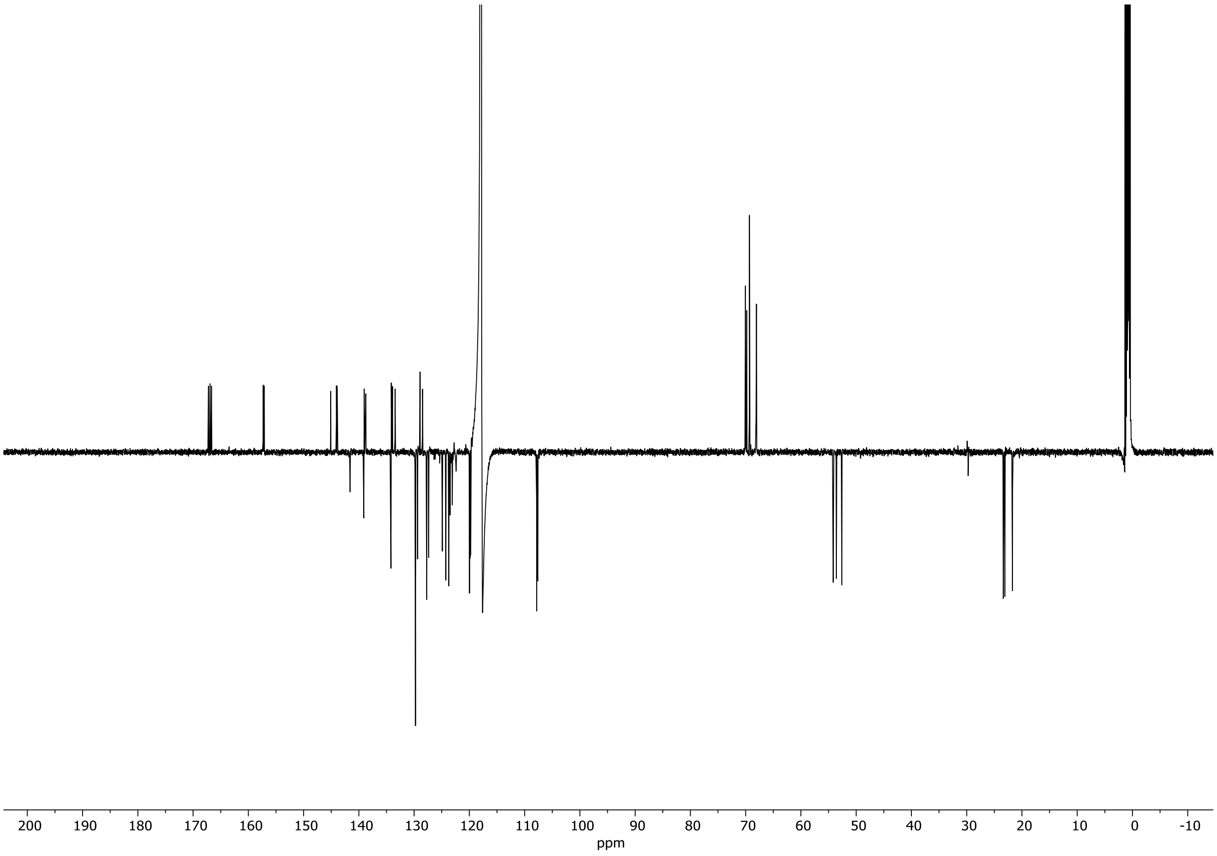

Spectrum S34. ^{13}C-NMR (151 MHz, CD_3CN, 298 K) of **1•**[Lu].

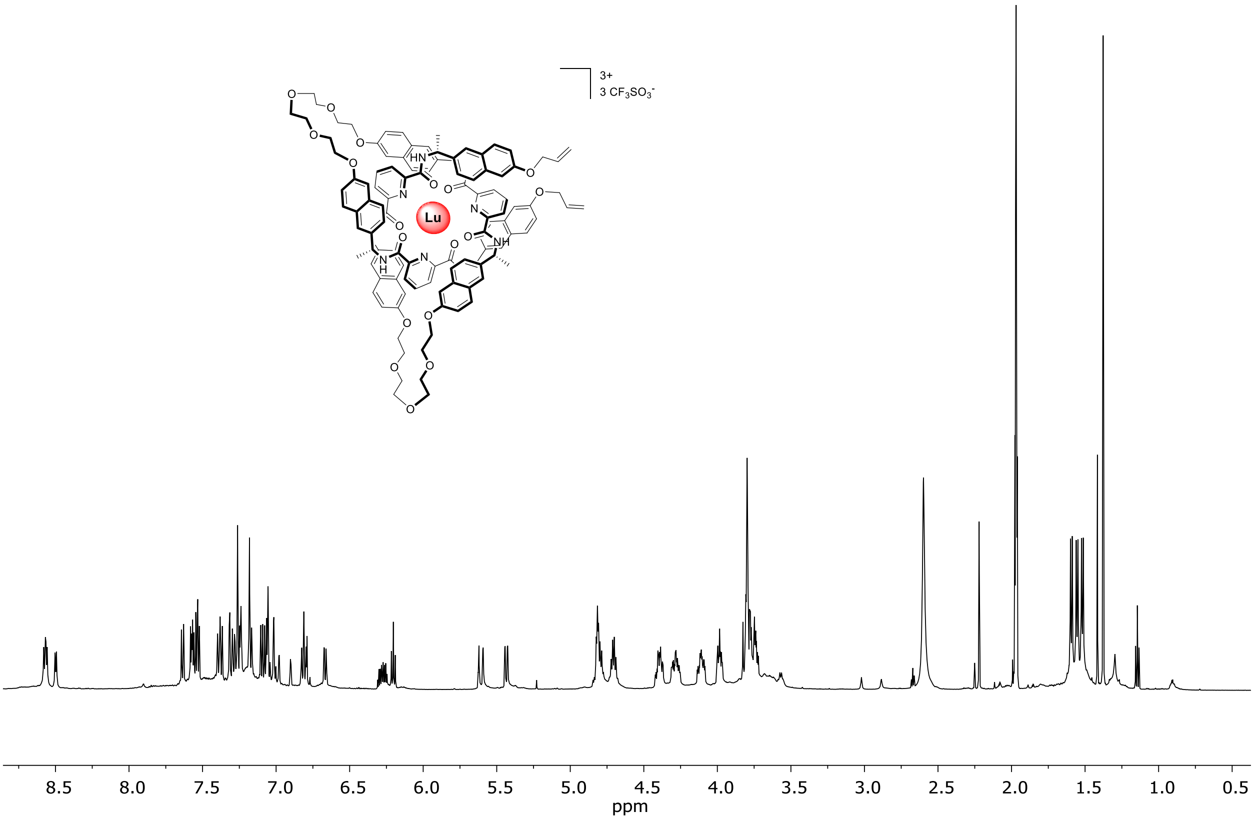

Spectrum S35. ^{1}H-NMR (600 MHz, CD_3CN, 298 K) of **3•**[Lu].

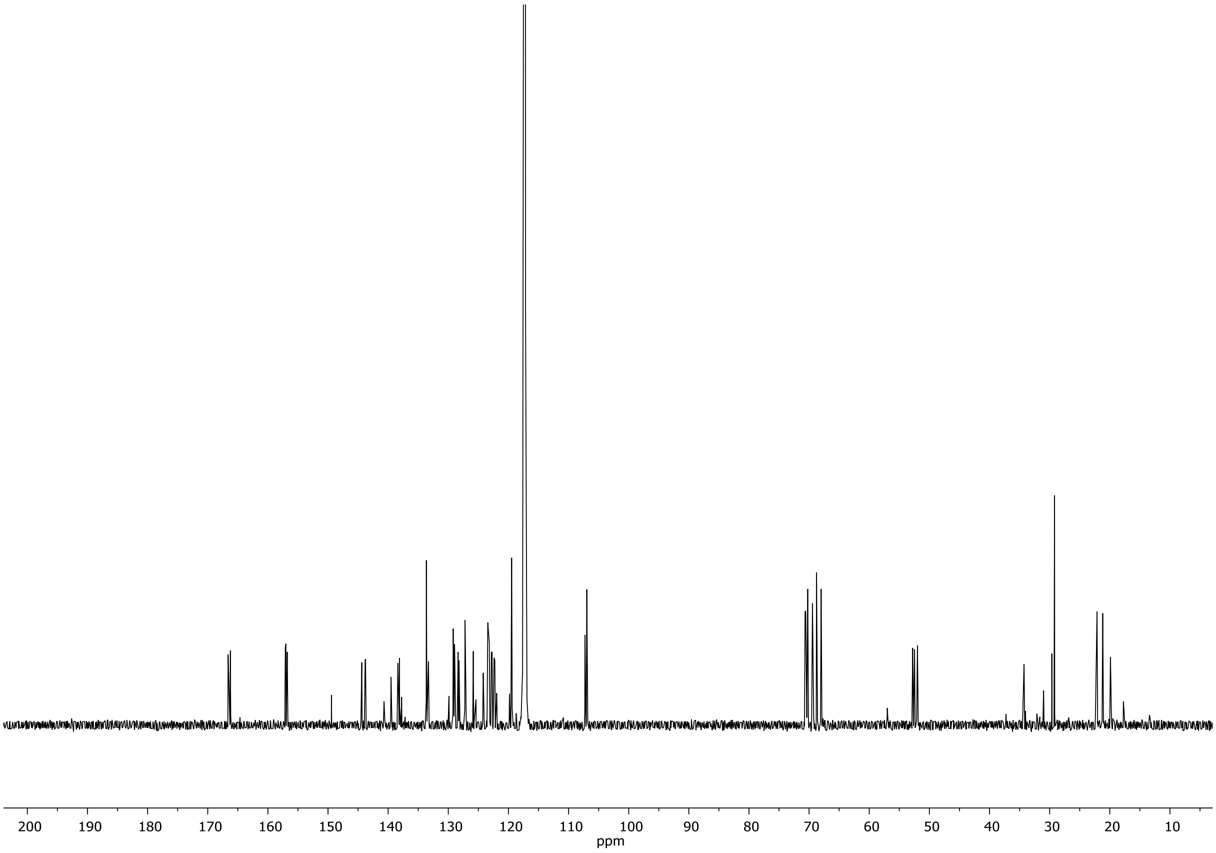

Spectrum S36. ^{13}C-NMR (151 MHz, CD_3CN, 298 K) of **3•**[Lu].

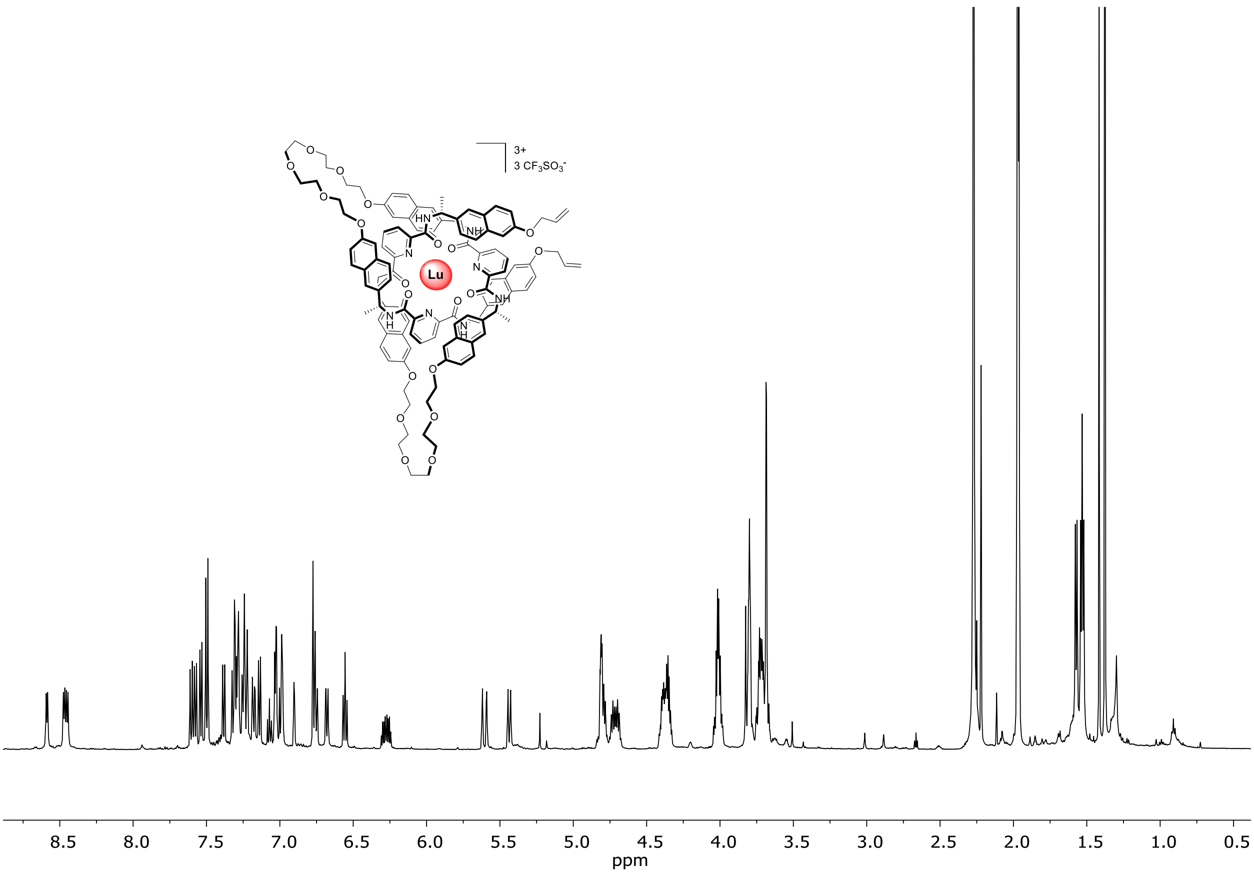

Spectrum S37. ^{1}H-NMR (600 MHz, CD_3CN, 298 K) of **4•**[Lu].

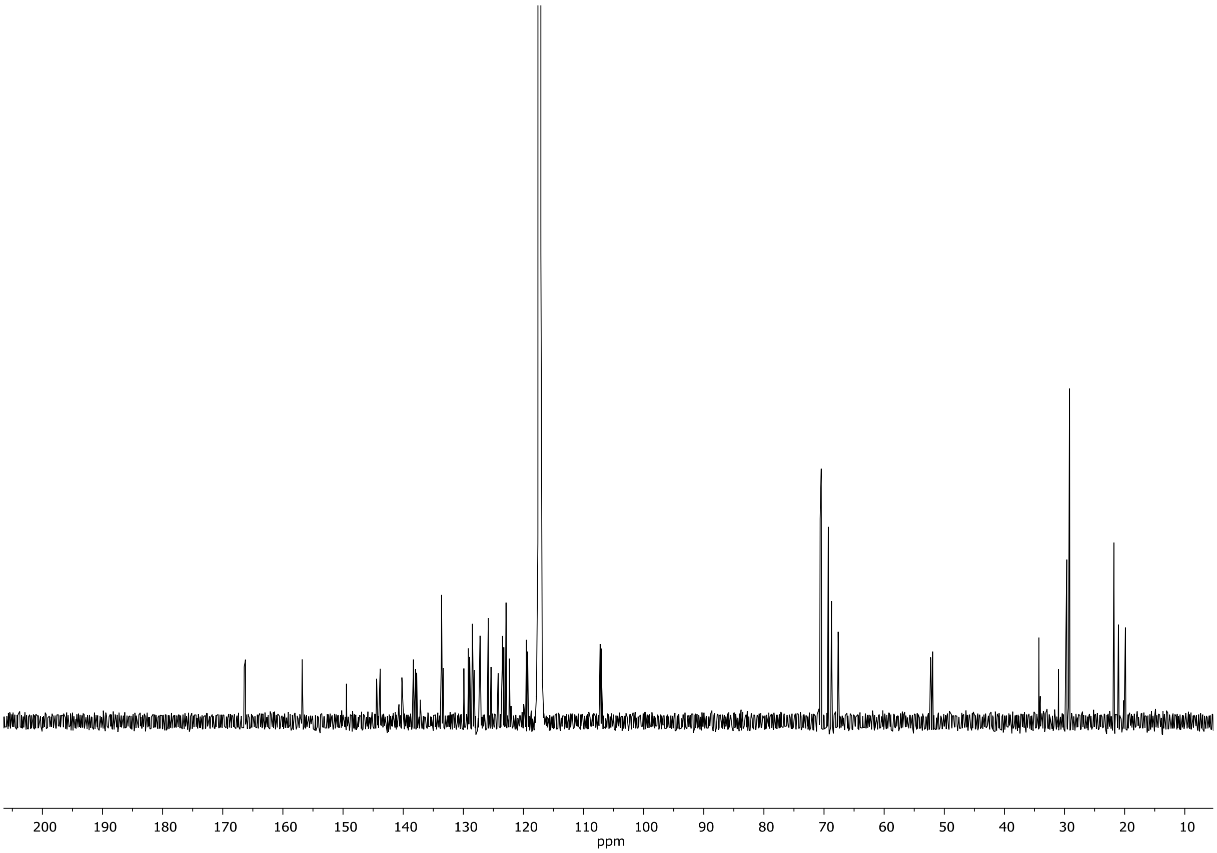

Spectrum S38. ^{13}C-NMR (151 MHz, CD_3CN, 298 K) of **4•**[Lu].

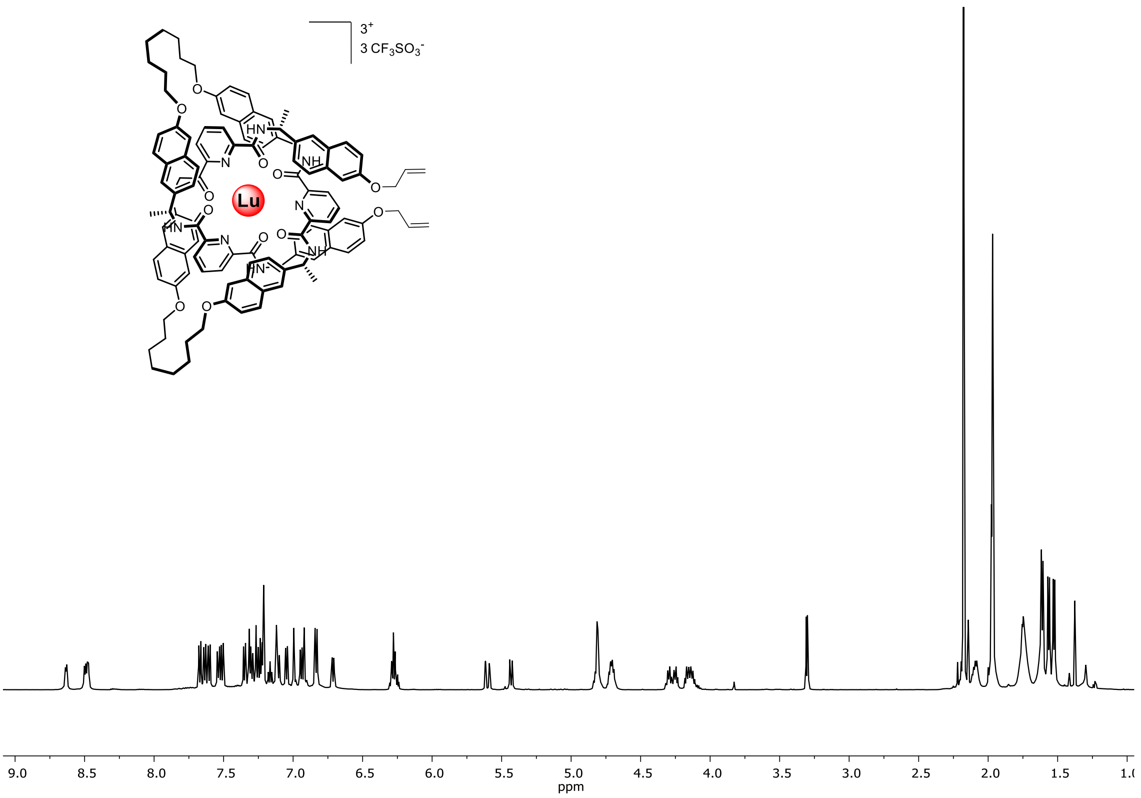

Spectrum S39. ^{1}H-NMR (600 MHz, CD_3CN, 298 K) of **5•**[Lu].

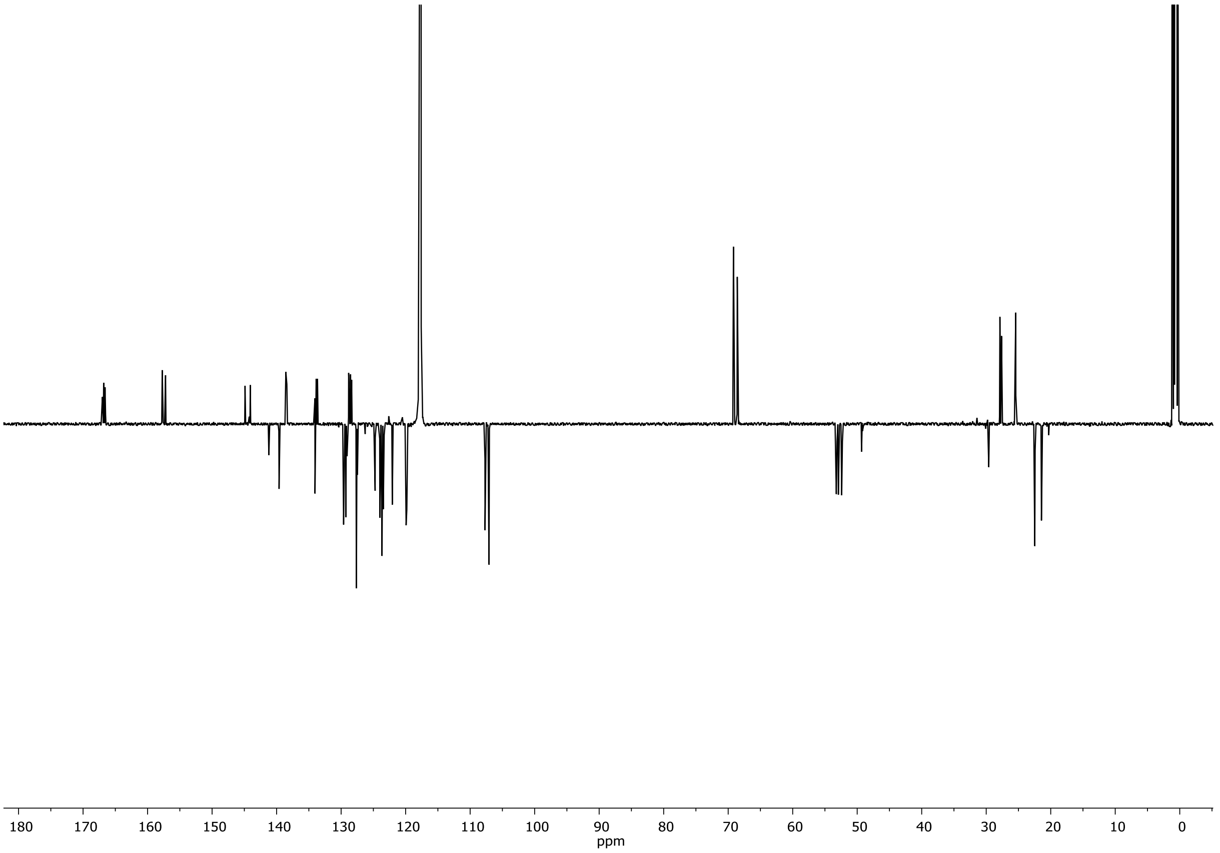

Spectrum S40. ^{13}C DEPT-NMR (151 MHz, CD_3CN, 298 K) of **5•**[Lu].

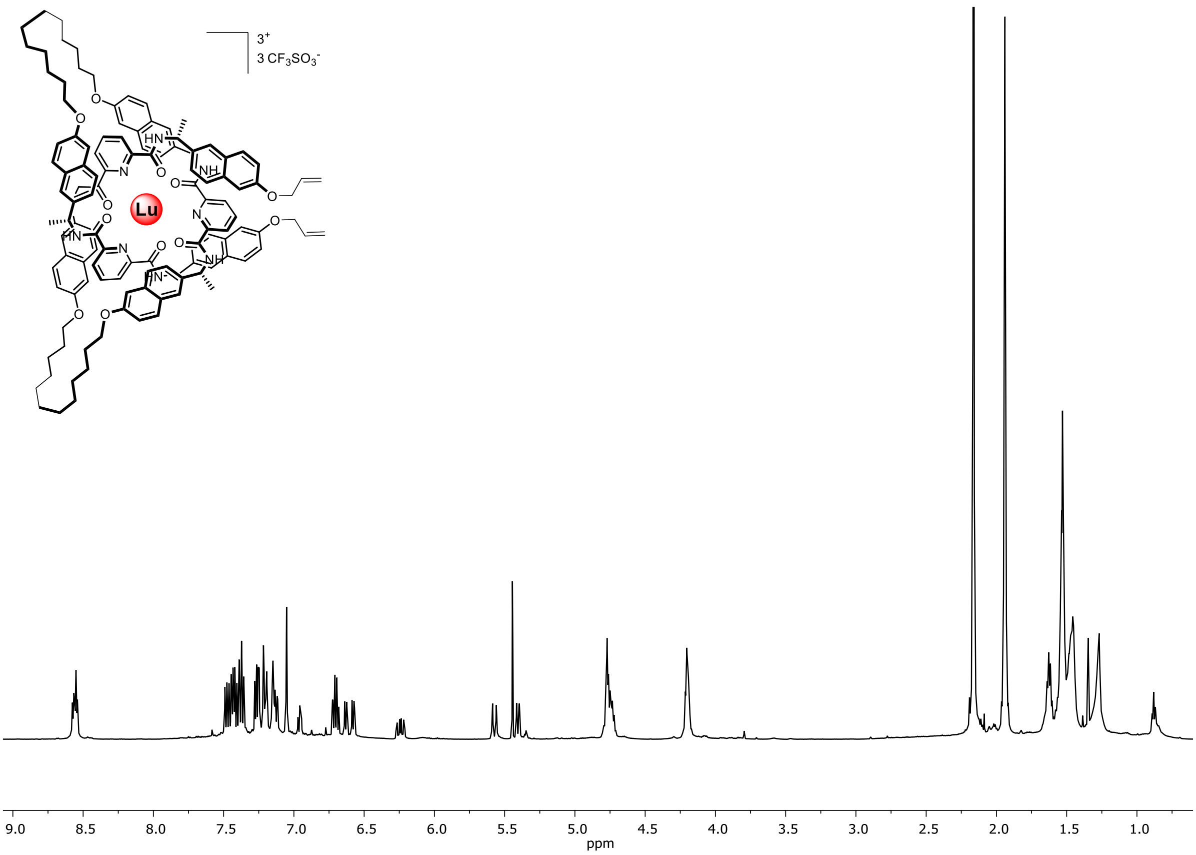

Spectrum S41. ^{1}H-NMR (600 MHz, CD_3CN, 298 K) of **6•**[Lu].

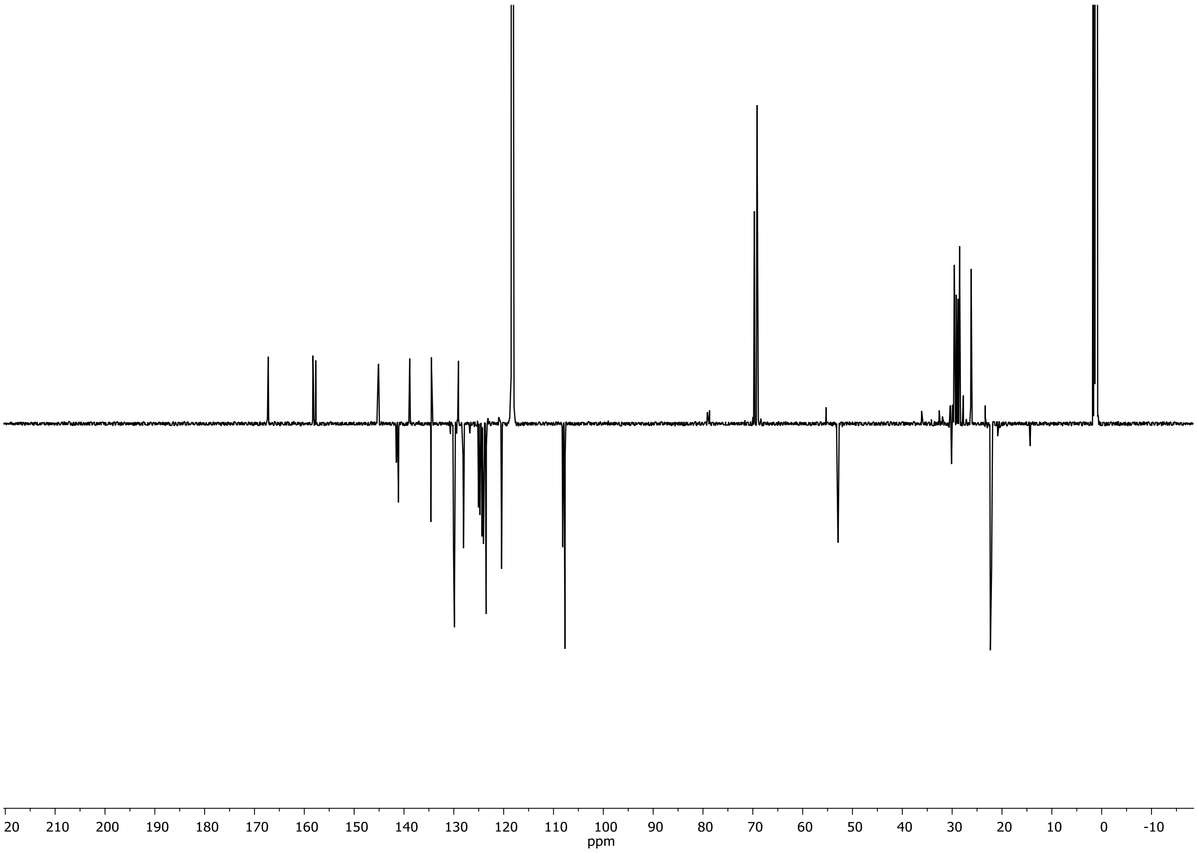

Spectrum S42. ^{13}C DEPT-NMR (151 MHz, CD_3CN, 298 K) of **6•**[Lu].

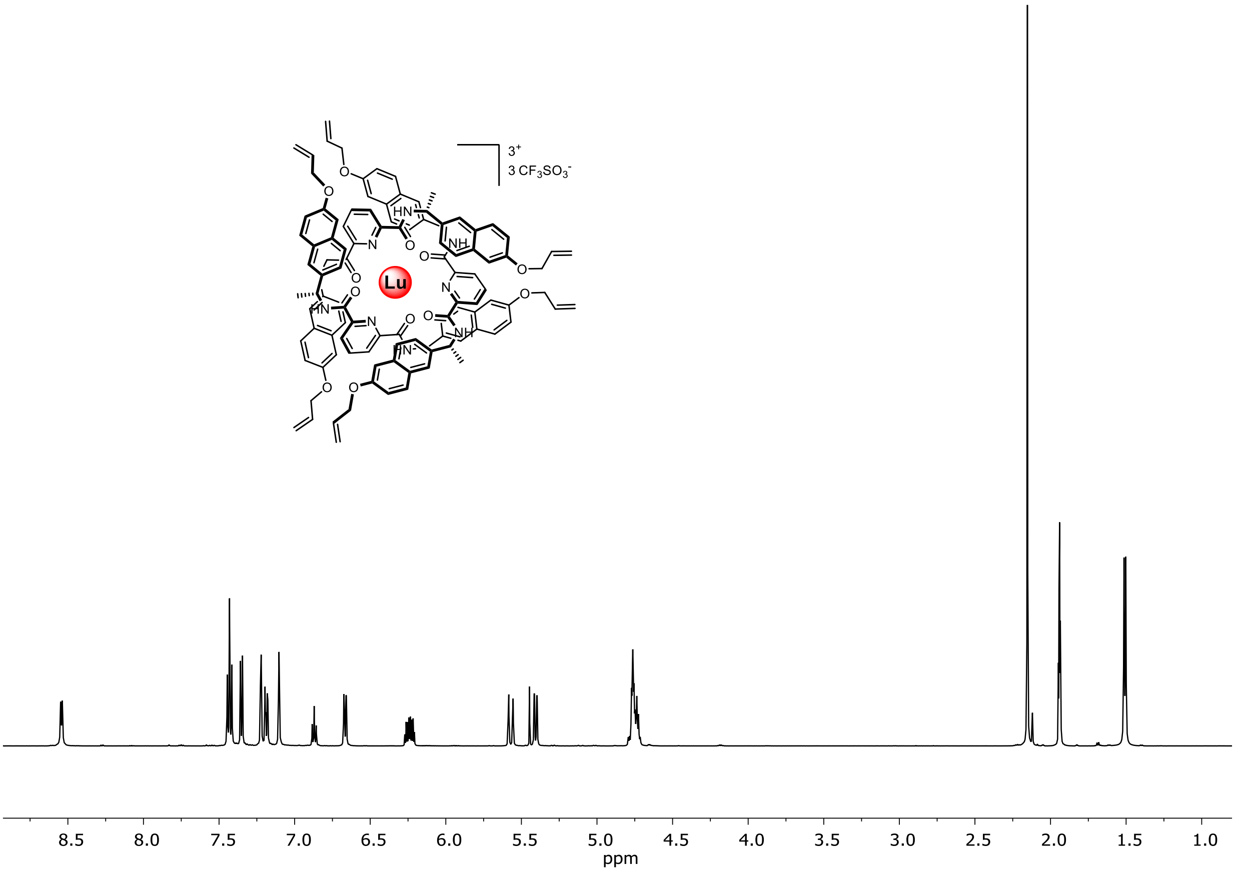

Spectrum S43. ^{1}H-NMR (600 MHz, CD_3CN, 298 K) of **7**$_3$•[Lu].

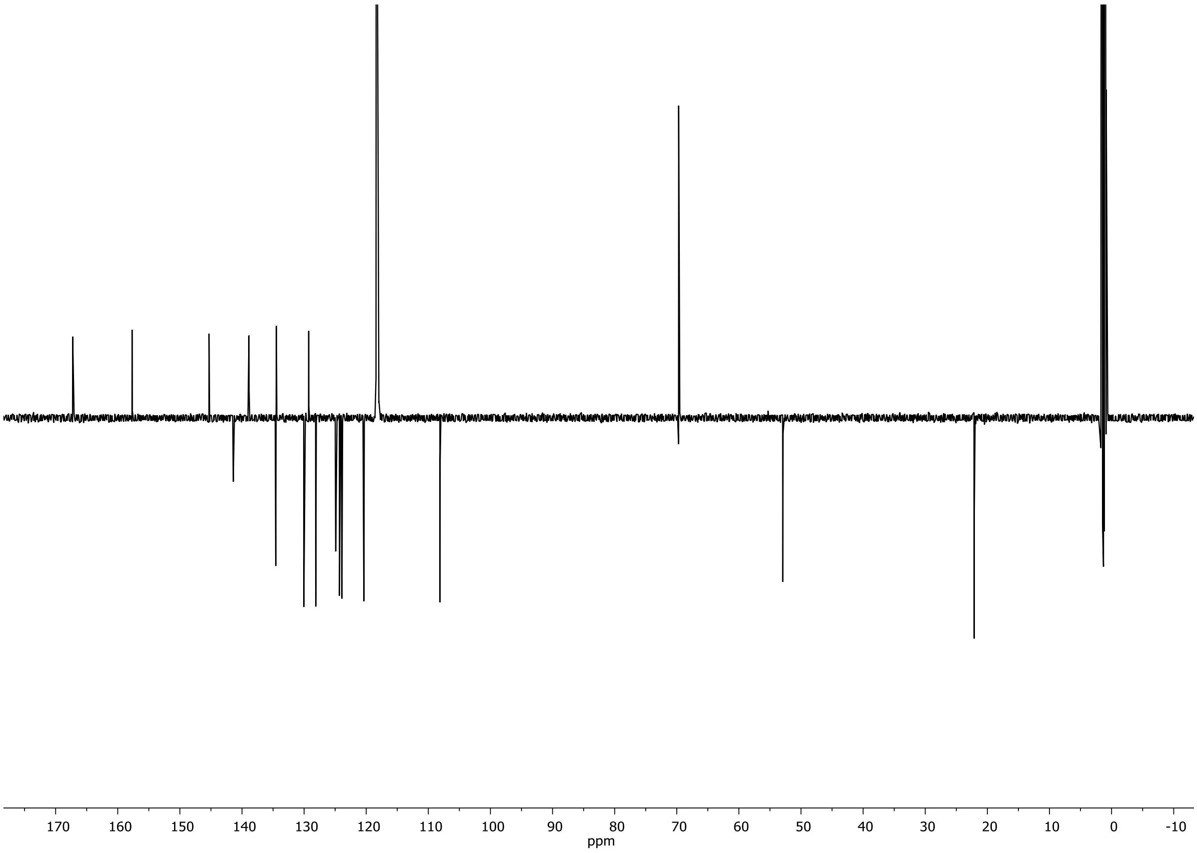

Spectrum S44. ^{13}C DEPT-NMR (151 MHz, CD_3CN, 298 K) of **7**$_3$•[Lu].

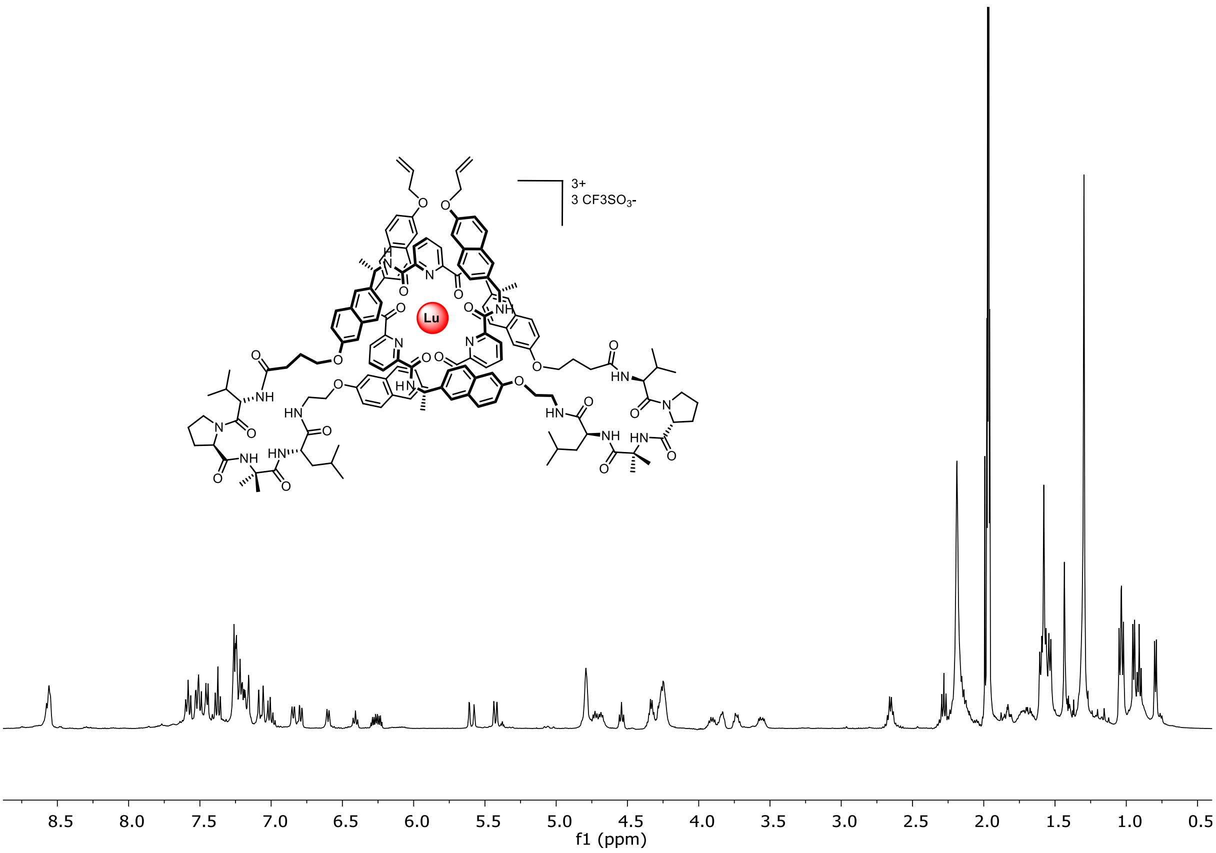

Spectrum S45. ^{1}H-NMR (500 MHz, CD_3CN, 298 K) of **8•**[Lu].

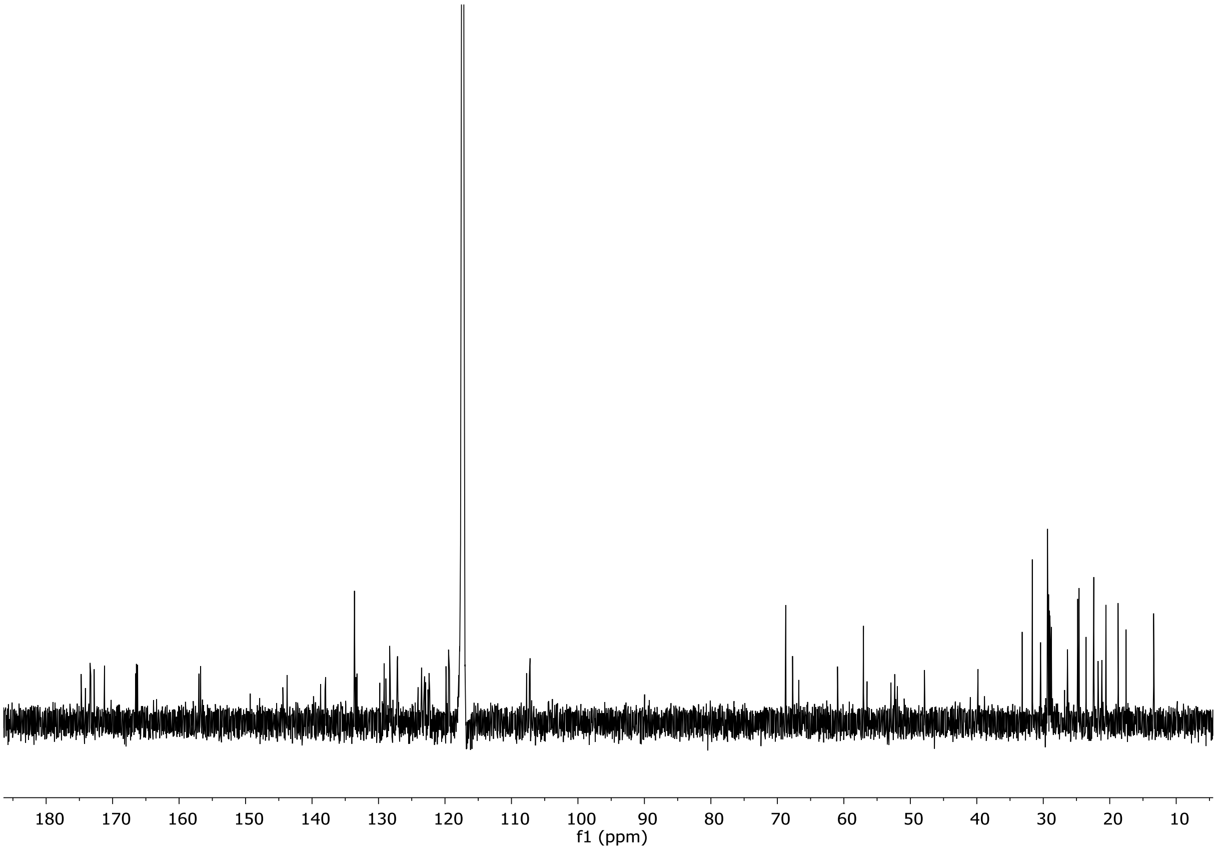

Spectrum S46. ^{13}C-NMR (125 MHz, CD_3CN, 298 K) of **8•**[Lu].

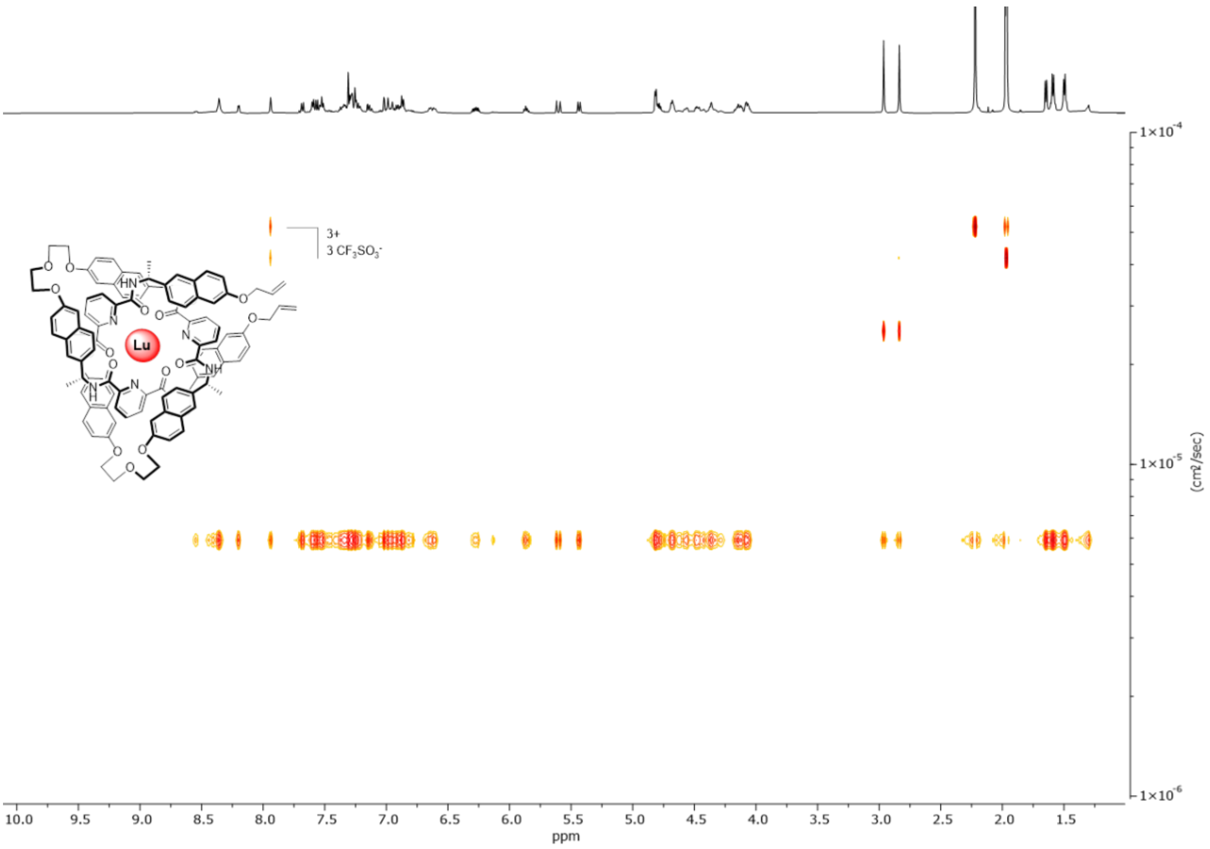

Spectrum S47. DOSY NMR (600 MHz, 298 K, MeCN-*d3*) of **1**•[Lu].

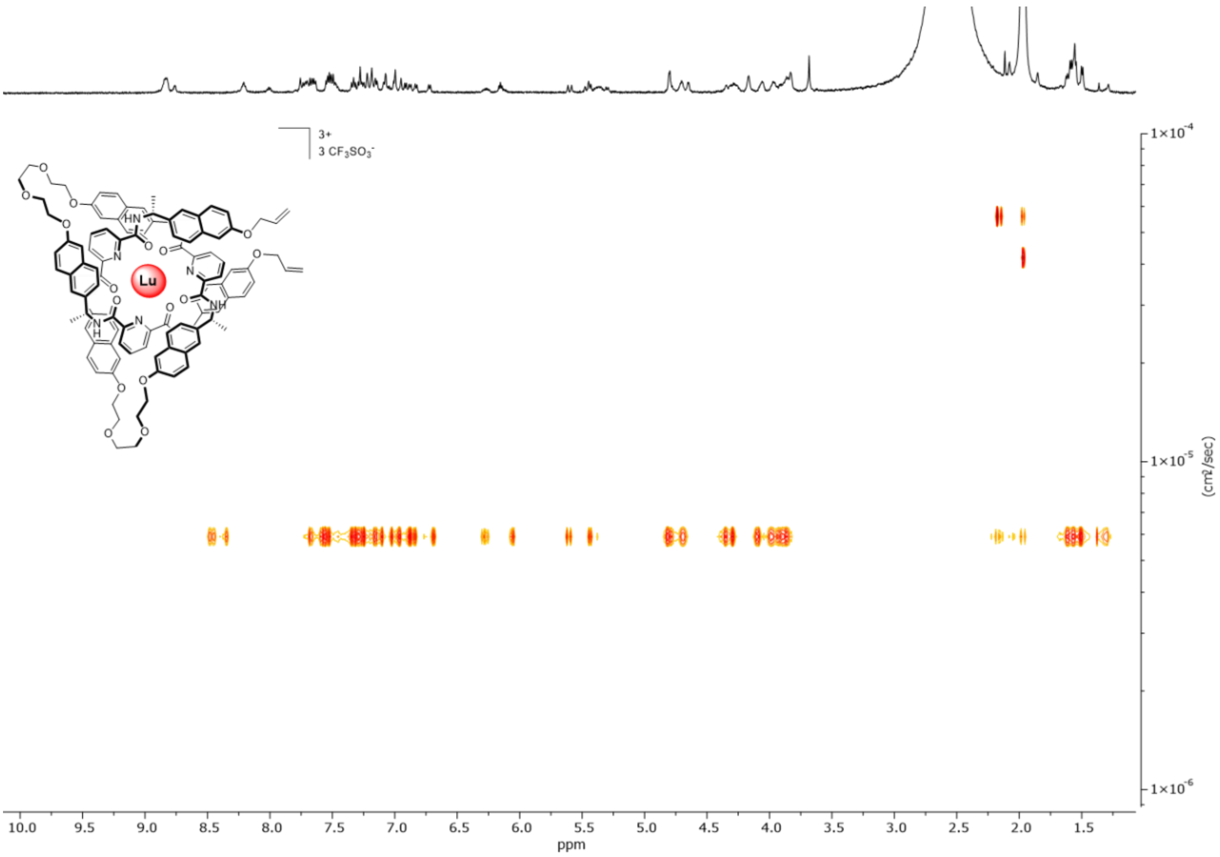

Spectrum S48. DOSY NMR (600 MHz, 298 K, MeCN-*d3*) of **2**•[Lu].

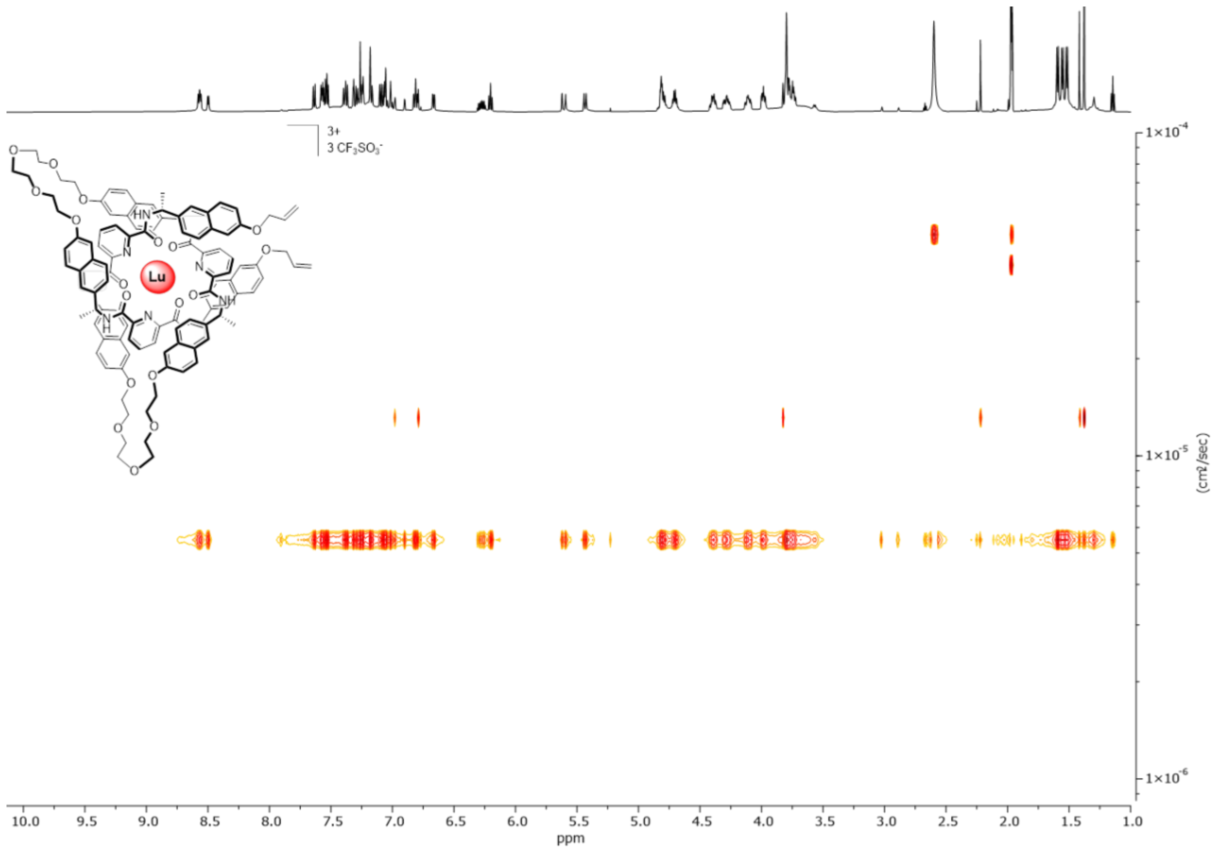

Spectrum S49. DOSY NMR (600 MHz, 298 K, MeCN-d_3) of **3**•[Lu].

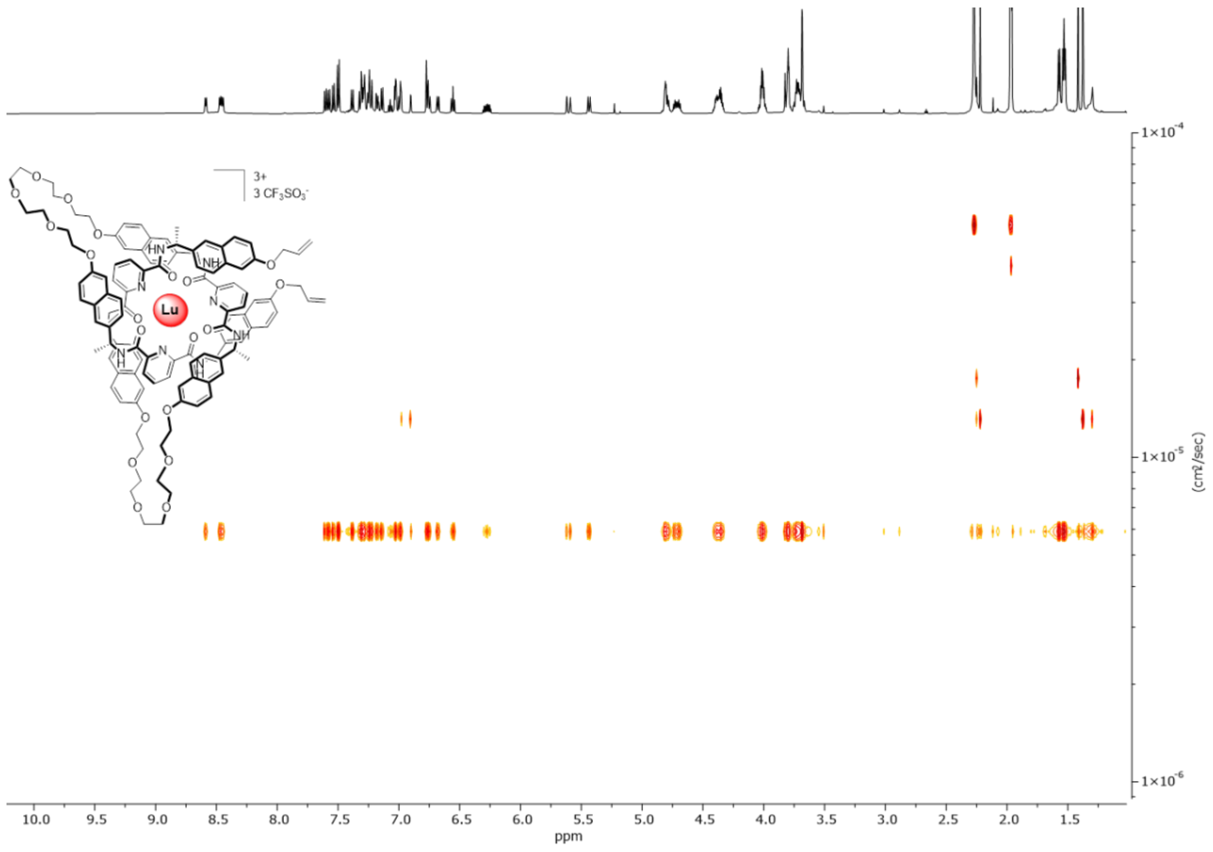

Spectrum S50. DOSY NMR (600 MHz, 298 K, MeCN-d_3) of **4**•[Lu].

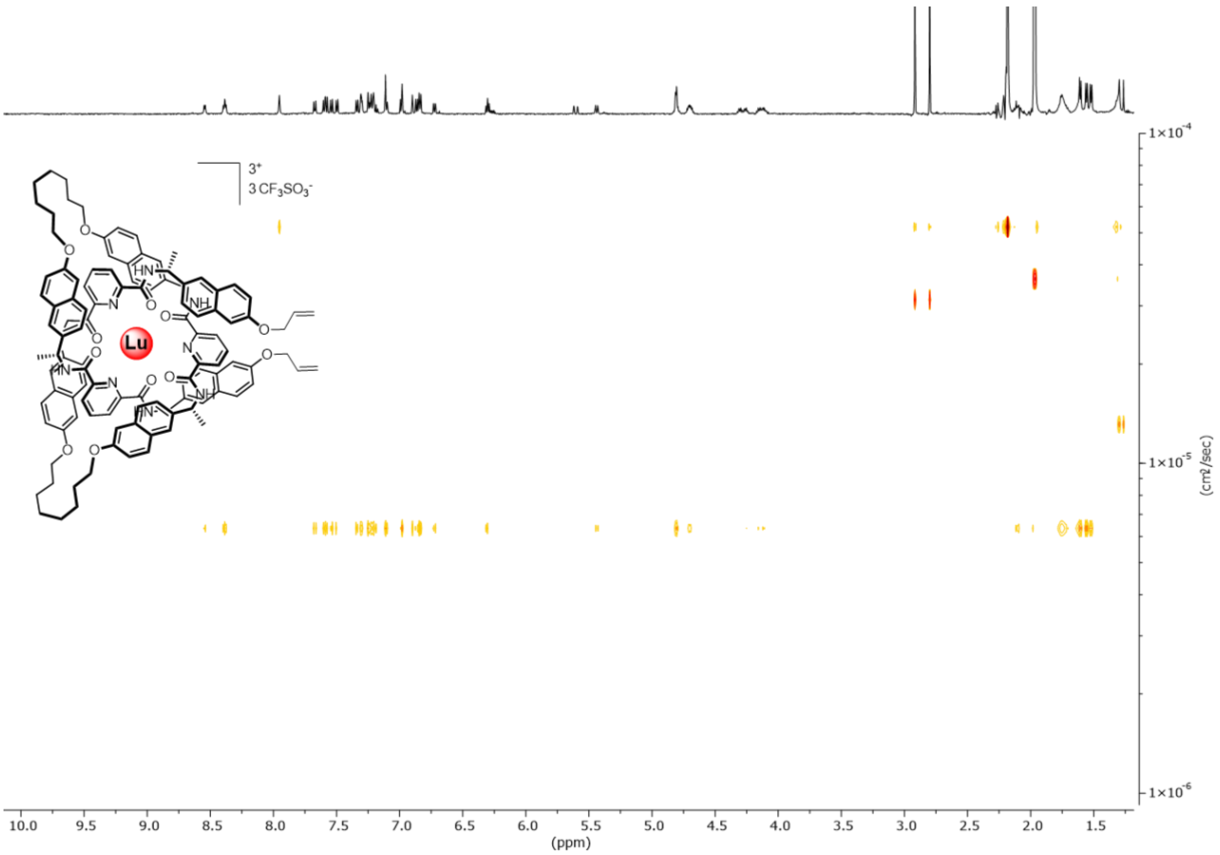

Spectrum S51. DOSY NMR (600 MHz, 298 K, MeCN-d_3) of **5**•[Lu].

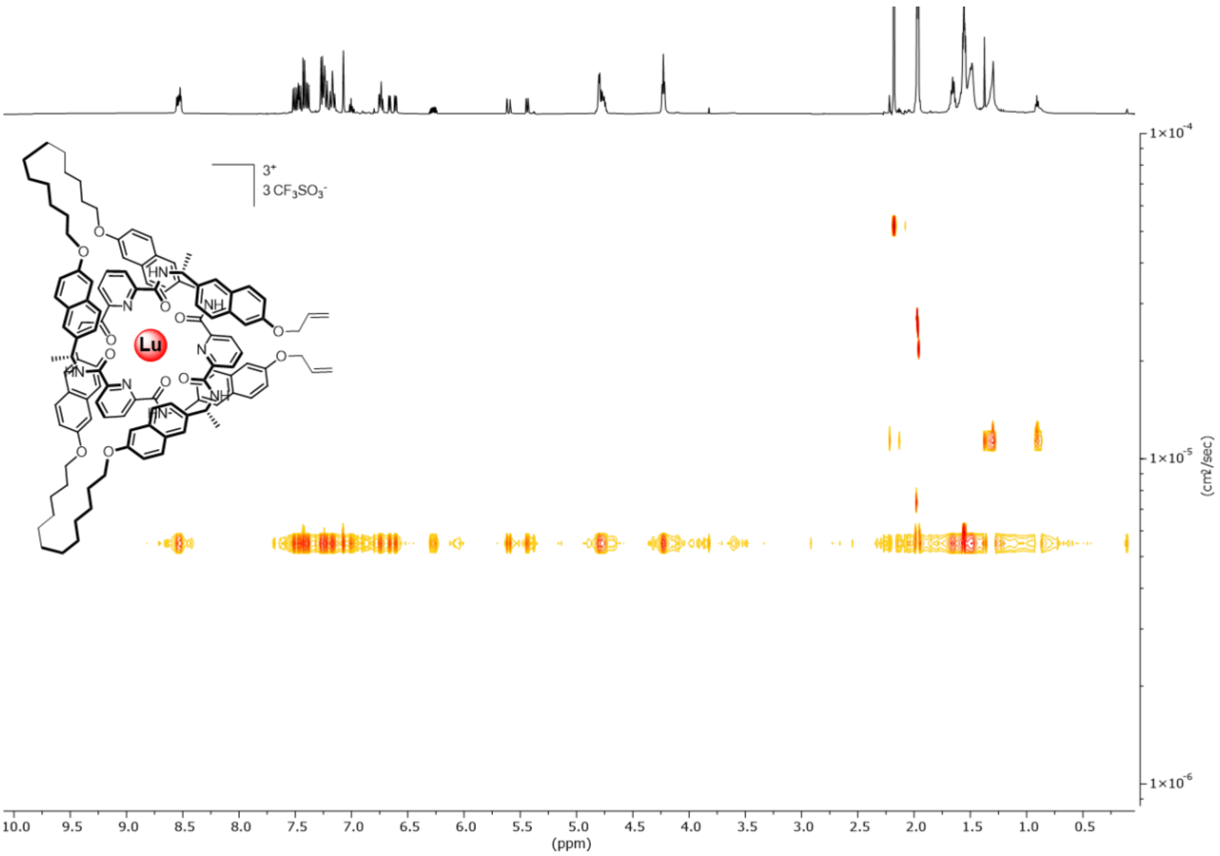

Spectrum S52. DOSY NMR (600 MHz, 298 K, MeCN-d_3) of **6**•[Lu].

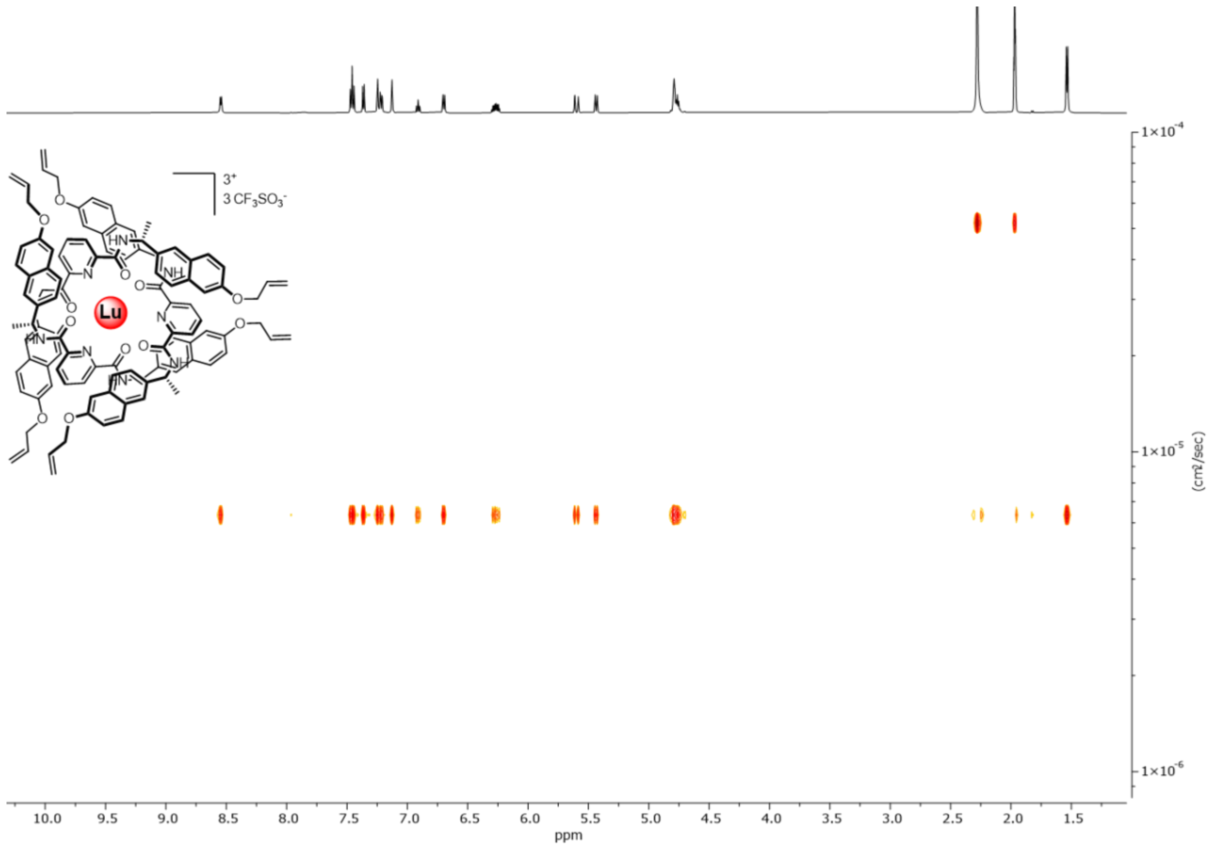

Spectrum S53. DOSY NMR (600 MHz, 298 K, MeCN-d_3) of **7**$_3$•[Lu].

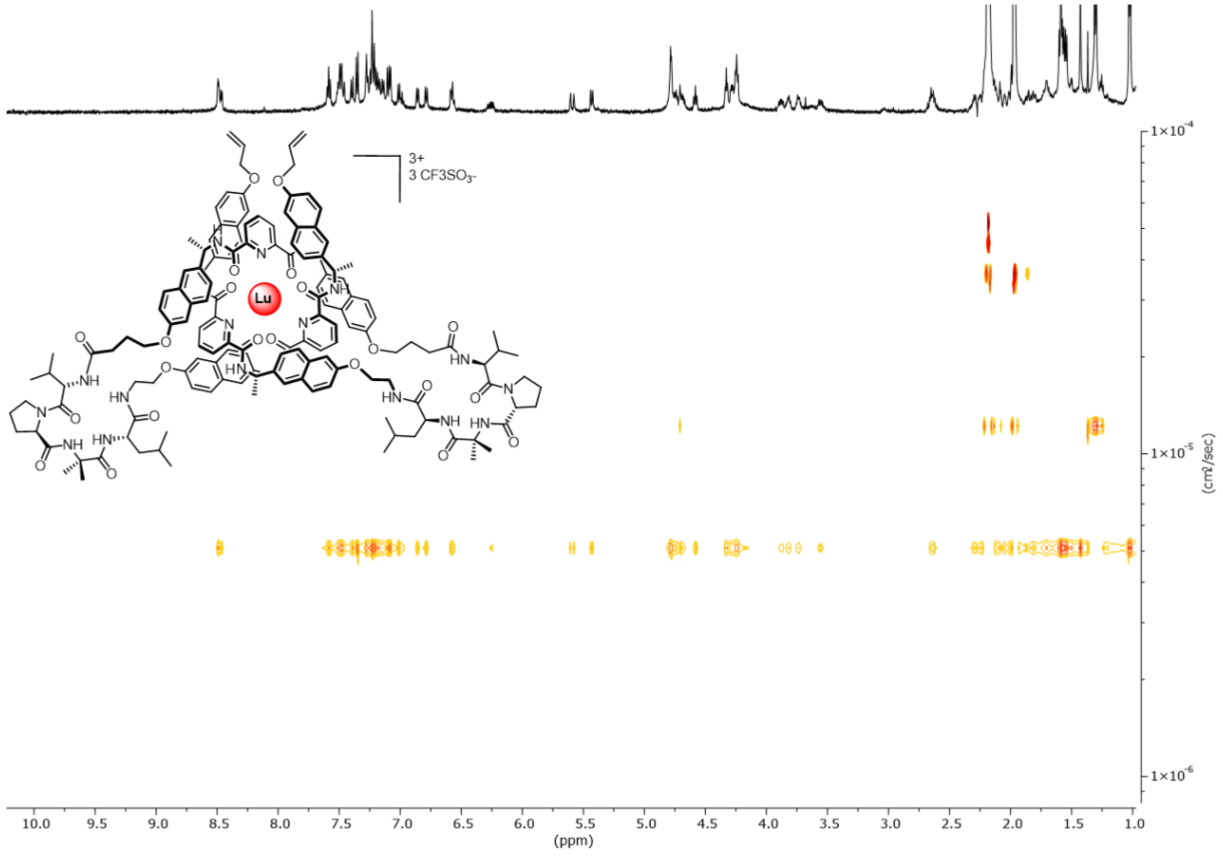

Spectrum S54. DOSY NMR (600 MHz, 298 K, MeCN-d_3) of **8**•[Lu].

S7. CIRCULAR DICHROISM AND ABSORPTION SPECTRA

S7.1 CD spectroscopy

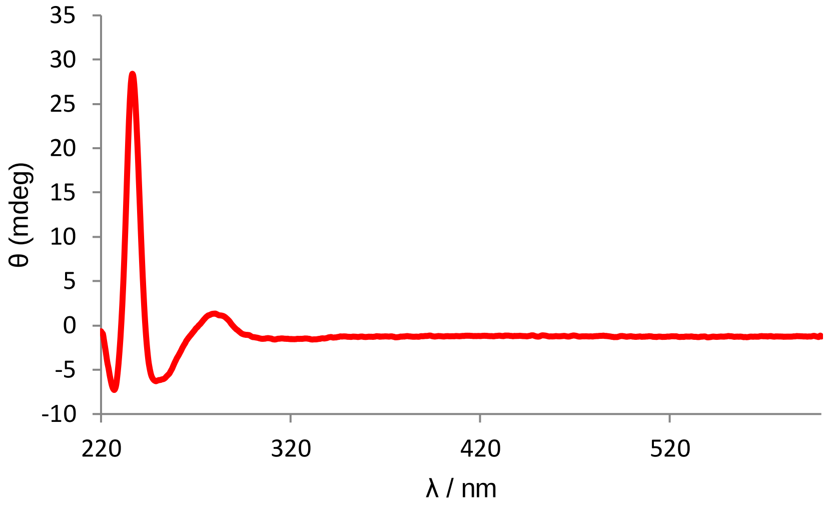

Spectrum S55. CD of knot **1**•[Lu] (0.1 mM, MeCN).

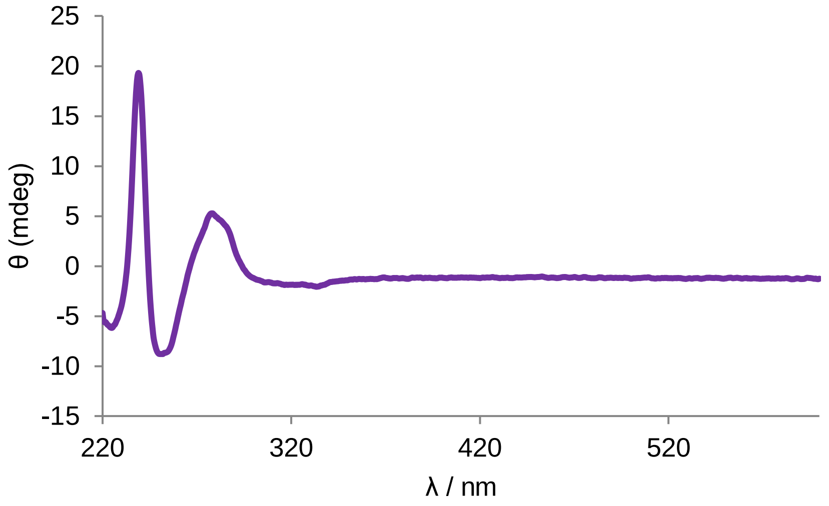

Spectrum S56. CD of knot **2**•[Lu] (0.1 mM, MeCN).

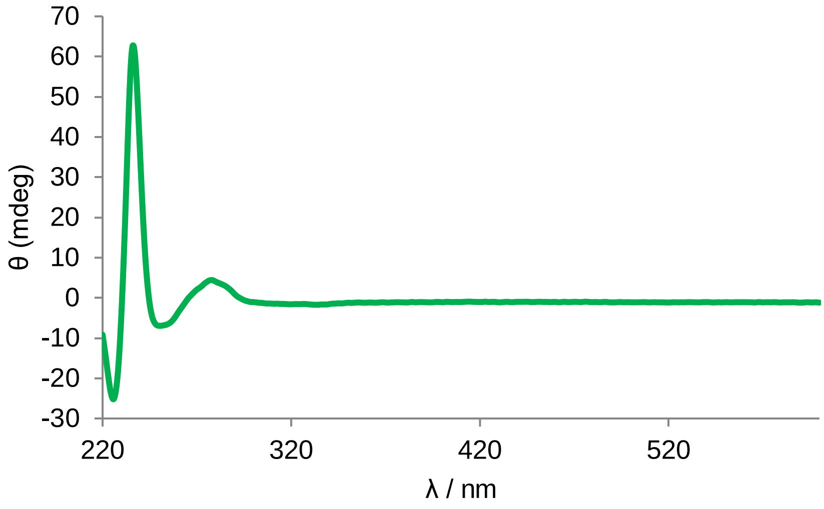

Spectrum S57. CD of knot **3**•[Lu] (0.1 mM, MeCN).

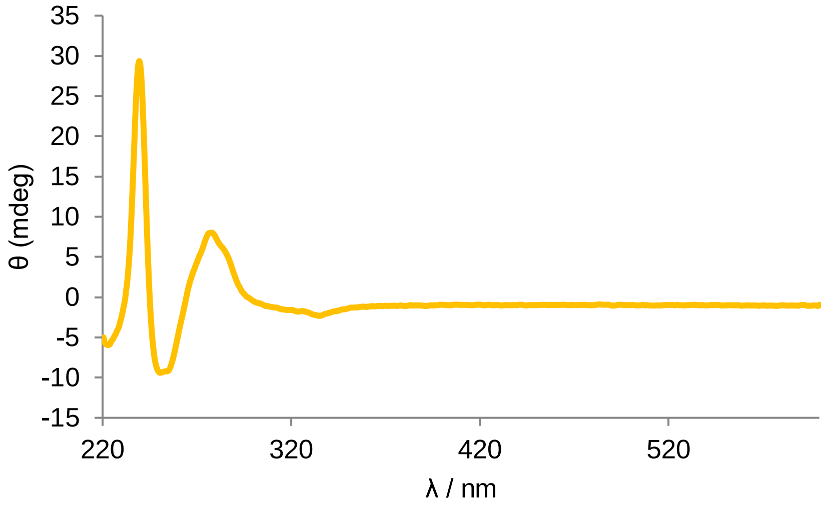

Spectrum S58. CD of knot **4**•[Lu] (0.1 mM, MeCN).

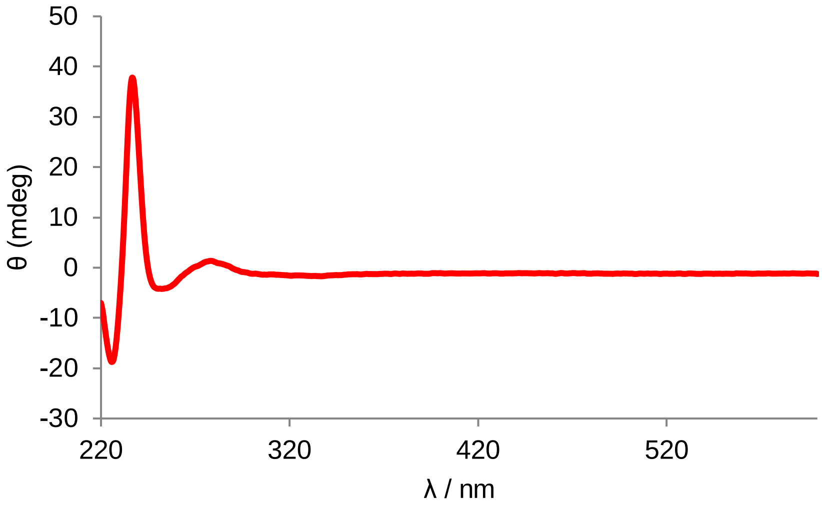

Spectrum S59. CD of knot **5**•[Lu] (0.1 mM, MeCN).

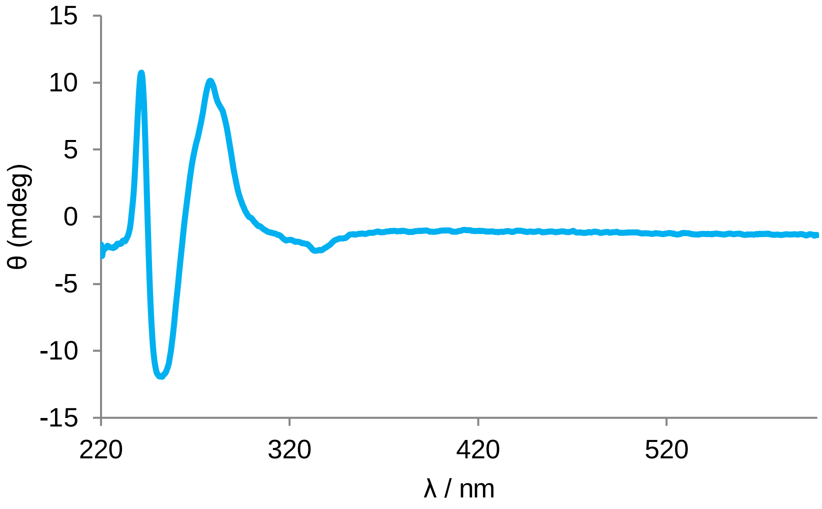

Spectrum S60. CD of knot **6**•[Lu] (0.1 mM, MeCN).

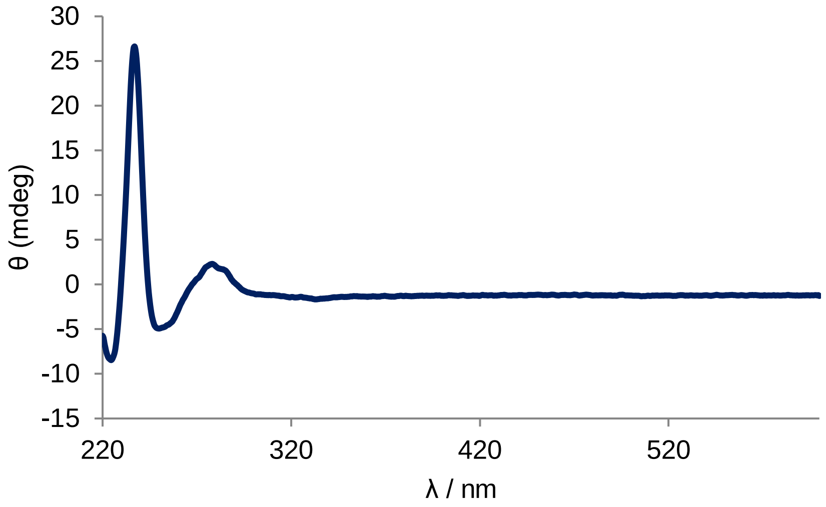

Spectrum S61. CD of circular helicate $\mathbf{7}_3$•[Lu] (0.1 mM, MeCN).

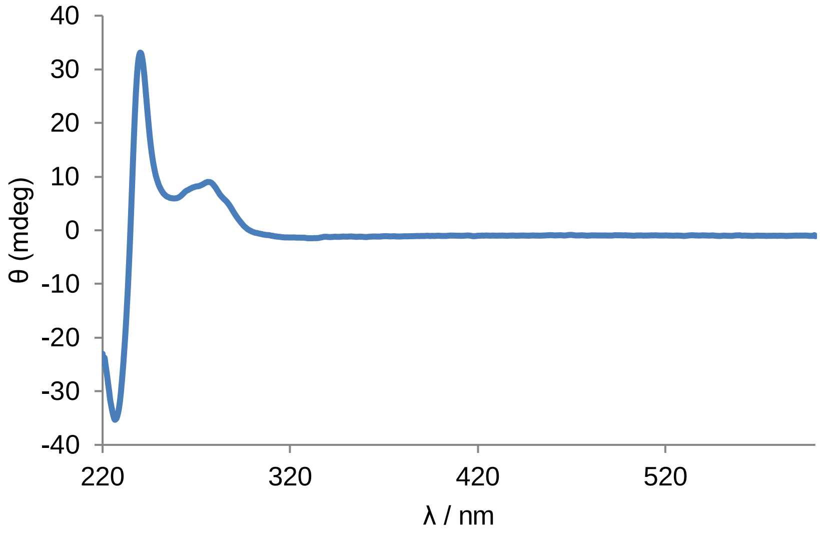

Spectrum S62. CD of metallopeptide ligand **8** (0.025 mM, CH_2Cl_2).

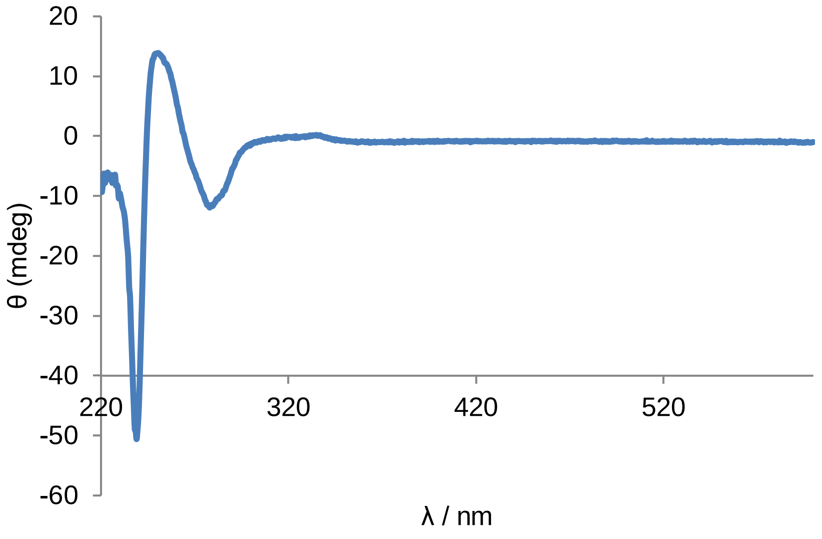

Spectrum S63. CD of metallopeptide knot **8**•[Lu] (0.1 mM, MeCN).

S7.2 UV/Vis spectroscopy

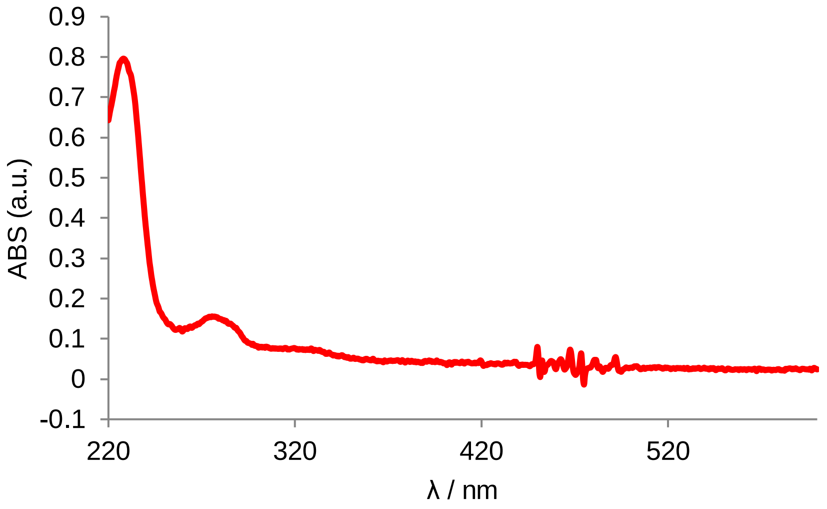

Spectrum S64. UV/Vis of knot **1**•[Lu](MeCN).

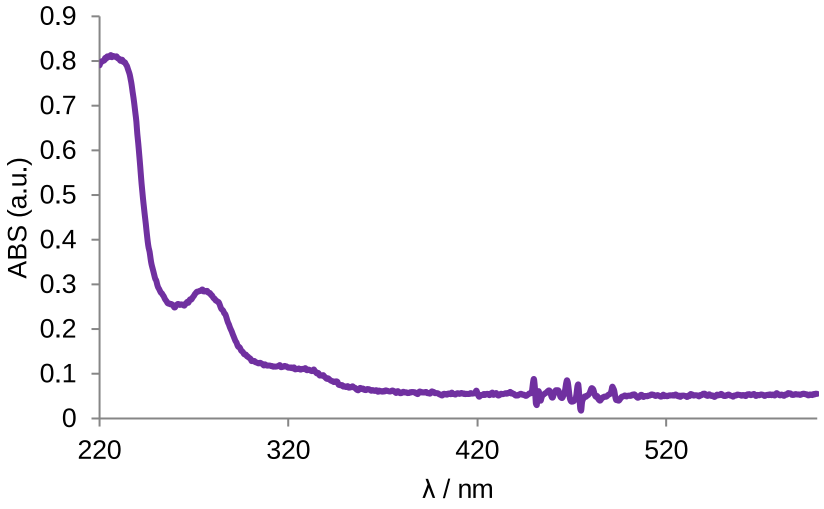

Spectrum S65. UV-Vis of knot **2**•[Lu] (MeCN).

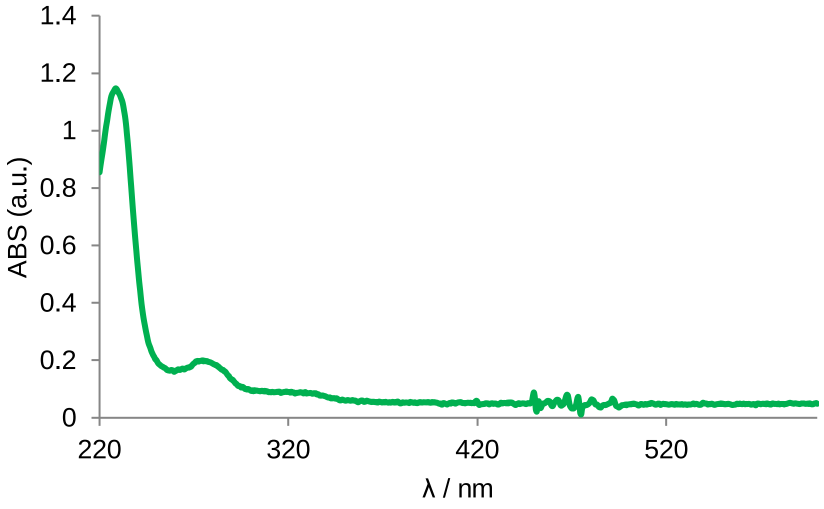

Spectrum S66. UV-Vis of knot **3**•[Lu] (MeCN).

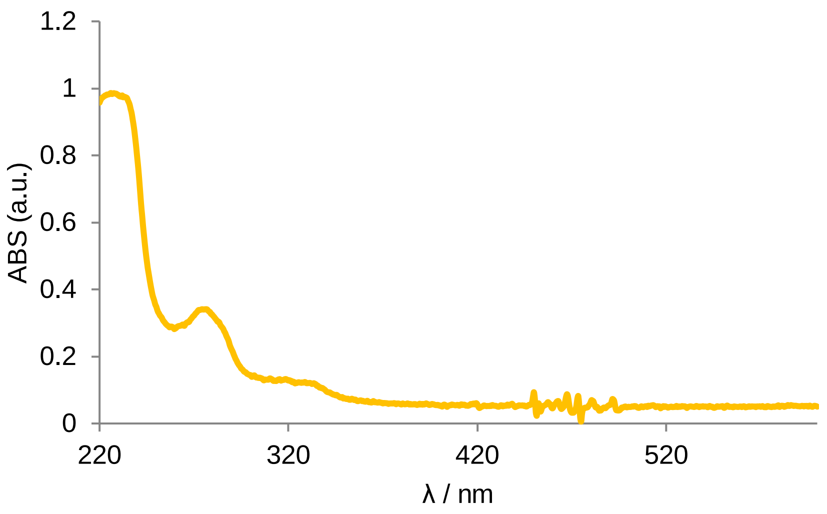

Spectrum S67. UV-Vis of knot **4**•[Lu] (MeCN).

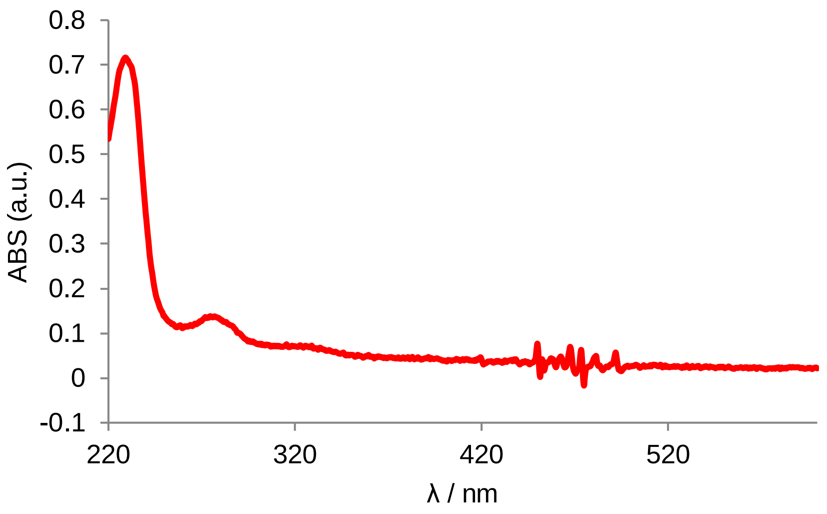

Spectrum S68. UV-Vis of knot **5**•[Lu] (MeCN).

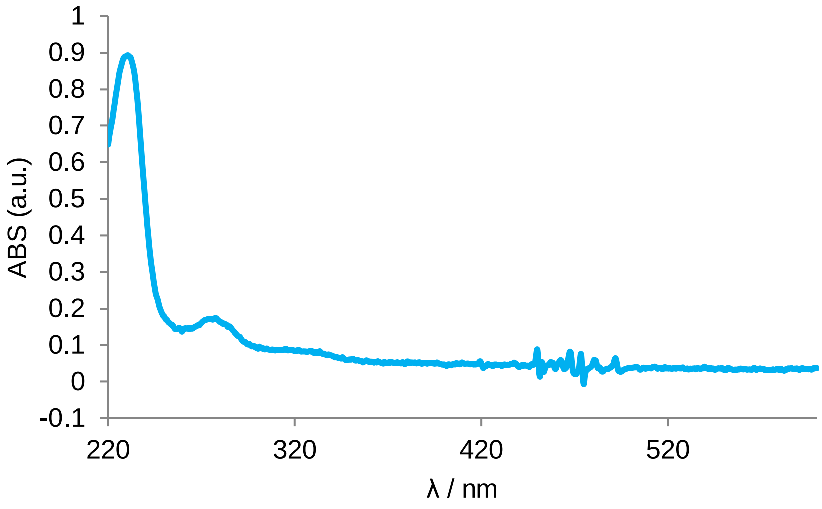

Spectrum S69. UV-Vis of knot **6**•[Lu] (MeCN).

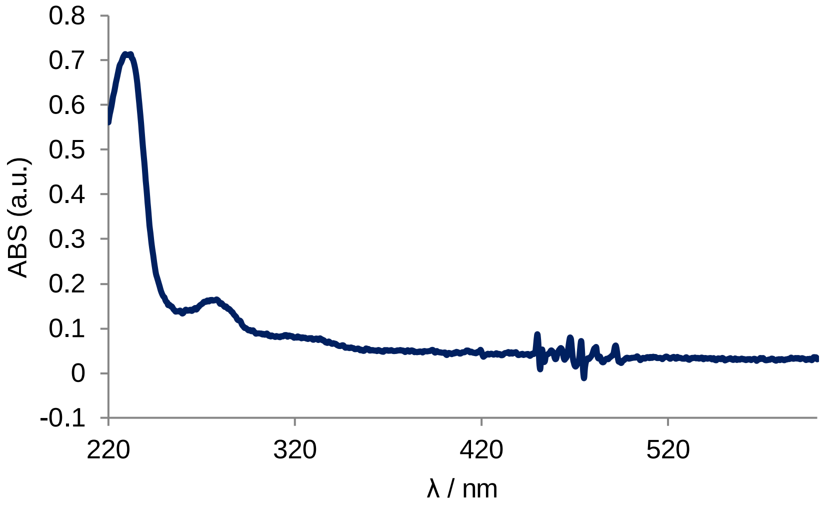

Spectrum S70. UV-Vis of circular helicate $\mathbf{7}_3$•[Lu] (MeCN).

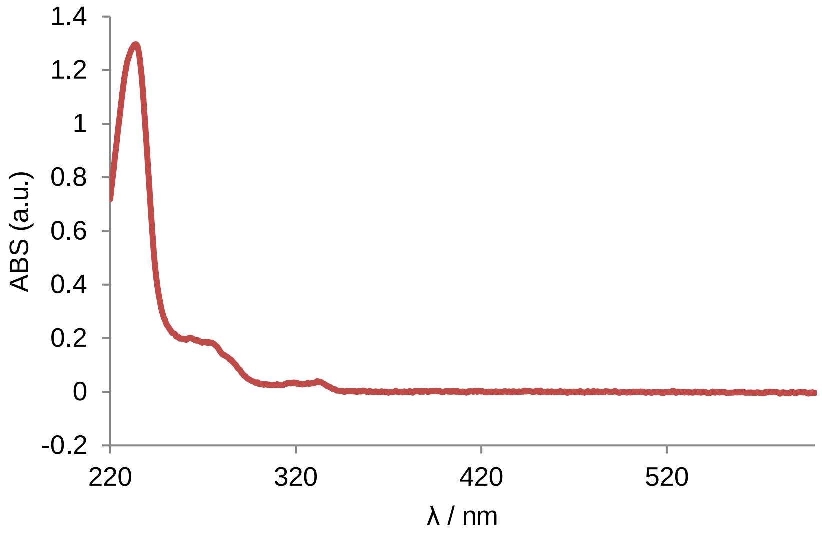

Spectrum S71. UV-Vis of metallopeptide ligand **8** (CH_2Cl_2).

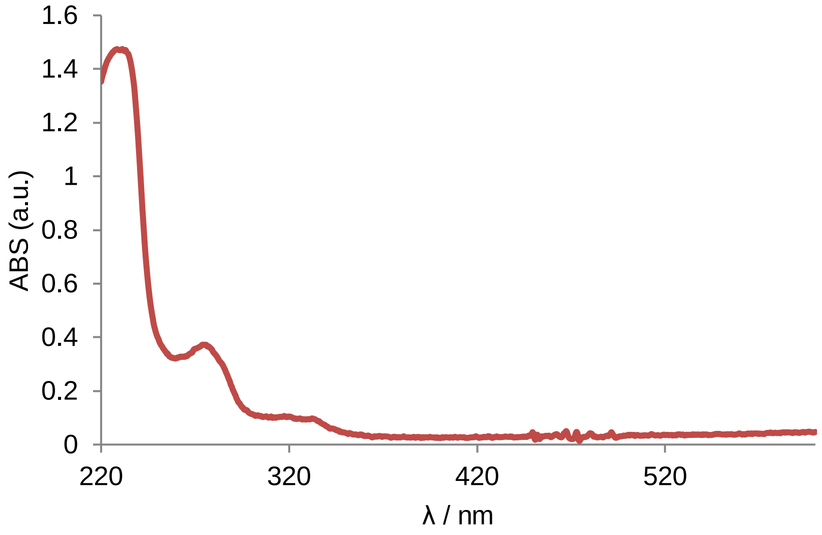

Spectrum S72. UV-Vis of metallopeptide knot **8•**[Lu] (MeCN).

S8. CRYSTALLOGRAPHY

Data Collection. X-Ray data for compound Λ-**4**•[Lu] were collected at a temperature of 100 K using a synchrotron radiation at single crystal X-ray diffraction beamline I19 in Diamond light Source,[9] equipped with a Pilatus 2M detector and an Oxford Cryosystems Cobra nitrogen flow gas system. Data was measured using GDA suite of programs.

Crystal structure determinations and refinements. X-Ray data were processed and reduced using CrysAlisPro suite of programmes. Absorption correction was performed using empirical methods (SCALE3 ABSPACK) based upon symmetry-equivalent reflections combined with measurements at different azimuthal angles.[10] The crystal structures were solved and refined against all F^2 values using the SHELX and Olex 2 suite of programmes.[11] All atoms except hydrogens were refined anisotropically. Hydrogen atoms were placed in the calculated positions. Naphthalene and the aliphatic groups were found and modelled over two positions. The C-C and C-O 1,2 and 1,3 distances in the aliphatic chains were restrained using distance restrains (SHELX; DFIX and SADI). The atomic displacement parameters (adp) of the ligands were restrained using rigid body restrains (SHELX RIGU and SIMU commands). The triflate anions were constrained to have the ideal structure. The adps were also restrained using rigid body restrains (SIMU and RIGU commands).

Compound Λ-**4**•[Lu] present medium size voids filled with a lot of scattered electron density. The solvent mask protocol inside Olex 2 software was used to account for the void electron density corresponding to the disordered solvent of the intermolecular space in the crystal structure. 45 electrons were accounted in a volume of 286 $Å^3$ .The electron count is compatible with 2 molecules of acetonitrile per void. The calculated flack parameter 0.050(5) shows that the absolute configuration of crystal structure Λ-4•[Lu] was correctly assigned.

CCDC 1997561 contains the supplementary crystallographic data for this paper. This data can be obtained free of charge via www.ccdc.cam.ac.uk/conts/retrieving.html (or from the Cambridge Crystallographic Data Centre, 12 Union Road, Cambridge CB21EZ, UK; fax: (+44)1223-336-033; or deposit@ccdc.cam.ac.uk).

Table S1. Crystallographic information for Λ-**4**•[Lu]

Identification code	Λ-**4**•[Lu]
Empirical formula	$C_{123.64}H_{127.46}F_9LuN_{9.82}O_{29}S_3$
Formula weight	2657.11
Temperature/K	100(2)
Crystal system	orthorhombic
Space group	$P2_12_12_1$
a/Å	13.2976(2)
b/Å	25.7772(2)
c/Å	38.6074(7)
α/°	90
β/°	90
γ/°	90
Volume/Å^3	13233.6(3)
Z	4
ρ_{calc}g/cm^3	1.334
μ/mm^{-1}	0.802
F(000)	5480.0
Crystal size/mm^3	0.12 × 0.09 × 0.08
Radiation	Synchrotron (λ = 0.6889)
2Θ range for data collection/°	3.062 to 51.006
Index ranges	-16 ≤ h ≤ 16, -32 ≤ k ≤ 32, -48 ≤ l ≤ 48
Reflections collected	175761
Independent reflections	27068 [R_{int} = 0.0932, R_{sigma} = 0.0658]
Data/restraints/parameters	27068/2940/1957
Goodness-of-fit on F^2	1.015
Final R indexes [I>=2σ (I)]	R_1 = 0.0780, wR_2 = 0.2195
Final R indexes [all data]	R_1 = 0.1254, wR_2 = 0.2503
Largest diff. peak/hole / e Å^{-3}	1.20/-0.66
Flack parameter	0.050(5)

S9. BCR CALCULATION

Backbone-to-crossing (BCR) ratios provide a measure of the tightness of a given knot. BCRs can also be used for open entanglements such as the overhand knots in this study (as is commonly done for knots in biology).[12] However, in such a case it is important to define what is the core part of the entanglement. For proteins, this is generally defined per amino acid – the moment when an amino acid is removed so that the minimized projection of the protein knot is no longer entangled is the minimum entanglement degree. For this study, we used a precise atomic definition and defined the peripheral naphthol oxygens as the endpoints of the entangled region, see Figure S11 for full numbering of an example ligand. This definition generated the approximate BCR values represented in Table S2 below.

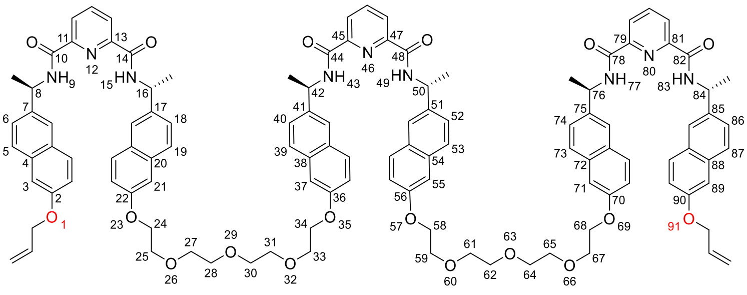

Figure S11. Example definition of numbering of ligand **3** to calculate BCR ratios.

Table S2. Backbone-to-crossing-ratios of the ligands used in this study

Compound	Backbone n	BCR for 3_1 knot
**1**	79	26
**2**	85	28
**3**	91	30
**4**	97	32
**5**	85	28
**6**	93	31
**8**	107	37

S10. REFERENCES

[1] A. Barrán-Berdón, S. Misra, S. Datta, M. Muñoz-Úbeda, P. Kondaiah, E. Junquera, S. Bhattacharya and E. Aicart, *J. Mater. Chem. B.* **2014**, *2*, 4640-4652.

[2] C. A. Hurley, J. B. Wong, J. Ho, M. Writer, S. A. Irvine, J. M. Lawrence, M. Jayne, S. L. Hart, A. B. Tabor, H. Hailes, *Org. Biomol. Chem.* **2008**, *6*, 2554-2559.

[3] E. Biron, F. Otis, J. Meillon, M. Robitaille, J. Lamothe, P. Van Hove, M. Cormier, N. Voyer, *Bioorg. Med. Chem.* **2004**, *12*, 1279-1290.

[4] H. Moon, Y. Jung, Y. Kim, B. W. Kim, J. G. Choi, N. H. Kim, M. S. Oh, S. Park, B. M. Kim, D. Kim, *Org. Lett*. **2019**, *21*, 389-3894.

[5] G. Zhang, G. Gil-Ramírez, A. Markevicius, C. Browne, I. J. Vitorica-Yrezabal, D. A. Leigh, *J. Am. Chem. Soc.* **2015**, *137*, 10437-10442.

[6] G. Gil-Ramírez, S. Hoekman, M. O. Kitching, D. A. Leigh, I. J. Vitorica-Yrezabal, G. Zhang, *J. Am. Chem. Soc.*, **2016**, 138, 13159-13162.

[7] D. A. Leigh, F. Schaufelberger, L. Pirvu, J. Halldin Stenlid, D. August, J. Segard, *Nature,* **2020**, *584*, 562-568.

[8] N. Katsonis, F. Lancia, D. A. Leigh, L. Pirvu, A. Ryabchun, F. Schaufelberger, *Nat. Chem*. **2020**, *12*, 939-944.

[9] H. Nowell, S. A. Barnett, K. E. Christensen, S. J. Teat, D. R. Allan. *J Synchrotron Radiat*.,**2012**, *19*, 435-441.

[10]. (a) G. M. Sheldrick, SADABS, empirical absorption correction program based upon the method of Blessing. (b) L. Krause, R. Herbst-Irmer, G. M. Sheldrick, D. Stalke, J*. Appl. Cryst.* **2015**, 48. (c) R. H. Lessing, *Acta Crystallogr.* **1995**, *A51*, 33-38.

[11] G. M. Sheldrick, *Acta Crystallogr.*, **2015**, *C71*, 3-8; b) O. V. Dolomanov, L. J. Bourhis, R. J. Gildea, J. A. K. Howard, H. Puschmann,. *J. Appl. Cryst.* **2009**, *42*, 339-341.

[12] E. Orlandini, *J. Phys. A: Math. Theor*. **2018**, *51*, 053001.

APPENDIX. XYZ COORDINATES FOR 8•[Lu]

Lu	-0.129700	-2.223400	-1.380400
O	-0.783100	-0.559400	0.101500
O	-2.314900	-2.078400	-2.134900
O	1.842200	-1.092100	-1.006800
O	0.720500	-2.893400	0.813200
O	-0.509700	-4.338800	-2.185500
O	0.455900	-2.096900	-3.632900
N	-0.474800	-0.017500	-2.422700
N	1.883200	-3.618600	-1.565100
C	-1.462100	-3.535300	1.431100
C	-0.592800	1.339500	-4.379000
H	-0.473600	1.463600	-5.457600
N	0.035100	-6.471900	-2.672500
H	0.770800	-7.168200	-2.756400
C	-0.863100	0.644300	-0.205800
C	0.346500	-5.240600	-2.286500
C	2.705000	-1.731900	-5.985700
O	8.915500	-3.688800	-0.140500
N	0.375400	-1.187900	-5.700300
H	0.115400	-0.386400	-6.268000
C	-3.324900	-2.320000	-1.440500
C	-0.345400	0.117800	-3.744500
C	4.063600	-2.979900	2.564300
H	3.863800	-2.027500	3.056100
C	4.153500	-5.145200	-1.828500
N	-4.553100	-2.028500	-1.863600
H	-5.373500	-2.194000	-1.272500
C	-3.743100	-3.465600	2.177500
H	-4.493100	-3.573900	2.963300
C	0.172700	-1.140700	-4.384200
C	-1.002300	2.421300	-3.596300
H	-1.227200	3.381200	-4.061800
N	-1.826600	-3.122600	0.213000
N	3.946400	-1.042800	-0.203500
H	4.760800	-1.581700	0.081900
C	-3.112200	-2.877700	-0.060800
N	0.546900	-4.079100	2.739200
H	-0.076100	-4.630500	3.322300
C	2.904800	-5.696200	-2.118100
H	2.828700	-6.727200	-2.467600
C	-4.108400	-3.008600	0.911200
H	-5.141300	-2.735300	0.695500
C	-4.526900	0.705100	-1.353300
H	-4.664800	-0.028300	-0.559800
C	4.601300	-5.438100	1.362000
H	4.810000	-6.402400	0.890200
C	4.245900	-3.821400	-1.385600
H	5.219900	-3.381200	-1.179600
C	-2.521100	-6.813800	-0.984300
H	-1.712000	-7.397900	-0.533700
C	3.552600	-2.505600	-5.220600
H	3.246500	-3.507000	-4.902800
C	-0.841300	1.014000	-1.661100

C	3.805200	0.318200	0.348100
C	-4.252400	2.605300	-3.396900
C	7.494600	-2.320300	1.234600
H	8.253400	-1.534600	1.198300
C	7.763300	-3.558100	0.586300
C	1.288300	-2.184300	-6.302600
H	1.094200	-3.135600	-5.788900
C	-6.152100	-1.482700	-3.632000
H	-6.363000	-0.801600	-4.471200
H	-6.217800	-2.522400	-3.997500
H	-6.916700	-1.347700	-2.854200
C	-2.401600	-3.743400	2.446900
H	-2.100000	-4.060500	3.446500
O	-3.296300	6.583400	-2.472700
C	-0.461400	2.575200	2.899600
H	0.471700	3.035700	2.524500
H	-1.297000	3.287100	2.778800
H	-0.338200	2.376200	3.976100
C	-1.290500	-6.823700	-3.214400
H	-1.402200	-6.265300	-4.158800
C	6.299500	-2.129100	1.895900
H	6.103100	-1.168200	2.379800
C	-1.127500	2.263800	-2.213600
H	-1.452800	3.104000	-1.603900
C	-4.730500	-5.738800	-0.761500
C	-4.347600	2.029400	-1.019900
H	-4.366200	2.363500	0.019200
C	-3.765300	-6.884000	1.179000
H	-2.944700	-7.443900	1.638400
C	1.775200	-4.882200	-1.971500
C	-4.171700	3.007800	-2.026600
C	0.031100	-3.514200	1.639400
C	3.086000	-3.954400	2.559600
C	3.068800	-3.087600	-1.254200
C	-4.764700	-1.196000	-3.051900
H	-4.002600	-1.488200	-3.788300
N	-0.911900	1.590700	0.730500
H	-0.967900	2.566900	0.457700
C	2.930700	-1.661600	-0.803800
C	-4.556700	0.280500	-2.706200
C	3.371000	-5.206800	1.948500
H	2.608800	-5.992200	1.943100
C	1.823400	-3.672700	3.357700
H	1.770100	-2.578000	3.485100
C	-4.889100	-6.585200	1.914200
H	-4.986600	-6.899800	2.956200
C	-4.616400	-5.324300	-2.116100
H	-5.427700	-4.742000	-2.563900
C	10.122500	-3.993700	0.574200
H	10.441400	-5.010100	0.281500
H	9.923100	-3.996000	1.660400
C	6.842100	-2.221600	-3.448700
H	7.515600	-2.838500	-2.848800
C	-3.500500	-5.647300	-2.859300
H	-3.424000	-5.307600	-3.896700

C	-4.437800	1.231700	-3.706500
H	-4.476900	0.933300	-4.759500
C	1.911800	-4.310400	4.748800
H	2.805900	-3.930000	5.268000
H	1.024400	-4.052000	5.354500
H	1.991300	-5.409600	4.674500
C	5.325600	-3.169700	1.947700
C	-2.440800	-6.414000	-2.306000
C	-0.763700	1.270900	2.156600
H	0.105300	0.597800	2.239600
O	-7.017000	-5.626800	2.167700
C	5.608700	-4.429400	1.325800
C	4.808900	-2.002200	-4.783200
C	-5.964000	-5.850500	1.334300
C	-3.648100	-6.482200	-0.184000
C	6.849700	-4.595300	0.646600
H	7.077100	-5.536700	0.139300
C	-3.863300	4.354200	-1.685000
H	-3.786200	4.605900	-0.627500
C	5.180900	-0.656600	-5.109400
C	-3.814400	4.914200	-4.050900
H	-3.672600	5.683200	-4.815000
C	-3.664900	5.291400	-2.681600
C	-5.877400	-5.424000	0.020200
H	-6.660200	-4.822900	-0.442900
O	8.238400	-0.250500	-3.146600
C	6.357600	-0.103700	-4.546100
H	6.631500	0.932900	-4.758000
C	7.154500	-0.853700	-3.695000
C	4.319800	0.096700	-5.958800
H	4.618200	1.106500	-6.253800
C	3.013400	0.308800	1.657400
H	3.577900	-0.230500	2.430200
H	2.871600	1.334700	2.020600
H	2.033800	-0.175600	1.521600
C	-4.097600	3.611900	-4.394500
H	-4.180300	3.330800	-5.449100
C	3.122600	-0.429300	-6.386800
H	2.466400	0.179400	-7.016800
C	5.699600	-2.772900	-3.990400
H	5.469300	-3.825600	-3.818100
C	11.242500	-3.023200	0.236100
H	11.310500	-2.891600	-0.853400
H	12.190100	-3.483400	0.578400
O	-7.856500	-1.164300	4.667300
C	8.648500	-0.610800	-1.820800
H	9.655900	-1.046800	-1.876300
H	7.964900	-1.367400	-1.402800
C	1.002300	-2.344100	-7.792100
H	1.691300	-3.092600	-8.216100
H	-0.033600	-2.688600	-7.955200
H	1.152600	-1.395400	-8.337900
C	-8.126200	-4.861800	1.713300
H	-8.990900	-5.210600	2.296900
H	-8.334900	-5.063800	0.649500

C	-1.298400	-8.320900	-3.529700
H	-2.261400	-8.598400	-3.988400
H	-0.490600	-8.577200	-4.238200
H	-1.165700	-8.915500	-2.608900
C	-7.884000	-3.359800	1.949600
H	-8.087700	-3.098100	2.998500
C	-5.429700	-1.067200	4.626600
H	-5.338900	-1.547900	5.602700
C	-6.675000	-0.852900	4.058700
C	-6.781900	-0.232800	2.782900
H	-7.772900	-0.060400	2.356300
C	-5.653500	0.203400	2.125500
H	-5.774300	0.739700	1.186200
C	-4.366800	0.060000	2.713100
C	-4.252000	-0.608000	3.973700
C	-2.955200	-0.740300	4.548300
H	-2.848900	-1.278500	5.494300
C	-1.849700	-0.184400	3.937300
H	-0.864600	-0.288300	4.404800
C	-1.974800	0.541900	2.720200
C	-3.209600	0.623100	2.117200
H	-3.318800	1.167800	1.184500
C	7.251000	1.215000	-0.788400
H	6.600700	0.454700	-0.320100
C	8.648200	0.634700	-0.951100
H	9.046000	0.360400	0.036600
H	9.333800	1.383700	-1.379800
C	-7.584900	0.460900	6.513500
H	-7.855100	0.574200	7.578200
H	-6.487400	0.555800	6.461500
C	-7.982200	-0.953900	6.082800
H	-7.388400	-1.700400	6.639300
H	-9.046500	-1.146000	6.287600
C	-8.246600	1.577000	5.670100
H	-8.537000	1.175100	4.685700
C	3.275900	1.269700	-0.718700
H	1.964700	2.354800	0.593700
C	2.357900	2.251800	-0.412400
C	3.318600	2.051400	-3.034300
C	1.919000	3.190200	-1.378600
C	3.761400	1.184500	-2.058300
C	2.386600	3.087400	-2.727800
C	1.037600	4.250700	-1.041400
H	4.476200	0.401800	-2.330300
H	2.255200	3.937900	-4.730100
H	3.684500	1.948200	-4.058400
C	0.604900	5.153300	-1.990000
H	0.712600	4.367500	-0.002600
H	-0.052700	5.968200	-1.689700
C	1.034000	5.023600	-3.340300
C	1.918600	4.011900	-3.692900
H	4.831600	0.622100	0.606400
O	0.617800	5.850800	-4.335400
C	-0.256600	6.932400	-4.037200
H	-0.652600	7.260600	-5.014400

H	-1.130600	6.580300	-3.459300
C	-2.892400	6.997000	-1.163800
H	-2.343700	7.938200	-1.341000
H	-2.182700	6.262500	-0.739000
C	-4.037600	7.240800	-0.220600
H	-4.844100	7.878600	-0.607300
C	-4.081300	6.761600	1.026700
H	-4.909800	6.990900	1.707000
H	-3.296400	6.100800	1.413700
C	0.409300	8.089600	-3.342100
H	-0.279100	8.892800	-3.045500
C	1.717700	8.199900	-3.089200
H	2.124600	9.082100	-2.581000
H	2.428500	7.415700	-3.376900
O	-7.096800	-2.170500	-0.477700
C	-8.251400	-2.001500	-0.066600
N	-8.695800	-2.514800	1.096500
H	-9.643000	-2.302800	1.393400
C	-9.302800	-1.248800	-0.889100
H	-10.143400	-0.977600	-0.237200
C	-9.796300	-2.128500	-2.057700
H	-8.932000	-2.360600	-2.706300
N	-8.789300	0.005800	-1.399900
H	-8.123900	-0.031200	-2.160900
C	-9.247800	1.210600	-0.969100
O	-10.020400	1.327100	-0.026600
C	-8.880200	2.432200	-1.857700
C	-7.823100	2.138800	-2.931500
H	-8.167700	1.381000	-3.656600
H	-6.880700	1.806400	-2.471100
H	-7.614700	3.062700	-3.495700
C	-10.197300	2.894700	-2.505800
H	-10.605100	2.098700	-3.151200
H	-10.018500	3.787700	-3.130600
H	-10.937900	3.139400	-1.727600
N	-8.401400	3.518800	-1.002400
H	-8.657100	4.459100	-1.280600
C	-7.502400	3.330200	-0.010700
O	-7.121600	2.214800	0.321700
C	-10.503700	-3.439800	-1.677700
H	-9.811600	-4.048500	-1.063700
C	-6.949800	4.598600	0.678800
N	-6.728700	4.328600	2.098400
C	-7.909300	4.715700	2.892500
C	-8.840000	5.406700	1.878800
C	-7.898200	5.804200	0.737400
H	-8.388300	3.834900	3.342900
H	-7.613800	5.394600	3.708300
H	-9.376600	6.259000	2.323700
H	-9.590100	4.689200	1.503000
H	-8.418400	6.034700	-0.204900
C	-5.536100	3.882500	2.568100
O	-4.572000	3.671900	1.832400
H	-5.988300	4.829800	0.197100
C	-5.457100	3.646200	4.095300

H	-5.804400	4.556500	4.610100
C	-4.015600	3.353100	4.568600
H	-3.563900	2.653900	3.847500
C	-3.998600	2.698500	5.954800
H	-4.545600	3.311900	6.693300
H	-2.957800	2.579400	6.304900
H	-4.460600	1.698100	5.932300
C	-3.199600	4.650000	4.565300
H	-3.613900	5.371900	5.294700
H	-3.202900	5.125000	3.570200
H	-2.149700	4.452300	4.845900
N	-6.418800	2.620100	4.459200
H	-6.429400	1.764800	3.909400
C	-7.317900	2.766600	5.470200
O	-7.370000	3.776300	6.169600
H	-10.485200	-1.505200	-2.656800
C	-10.821700	-4.242000	-2.947000
H	-11.297200	-5.206200	-2.692200
H	-9.908700	-4.454800	-3.533000
H	-11.519800	-3.681900	-3.597300
C	-11.780600	-3.193600	-0.860200
H	-11.577400	-2.703100	0.108600
H	-12.298500	-4.144700	-0.641800
H	-12.480500	-2.546800	-1.421900
O	11.736200	-0.531000	-1.000300
C	11.457900	-0.569800	0.194300
N	11.095900	-1.707600	0.841500
H	11.101500	-1.658100	1.864500
C	11.577800	0.679000	1.110500
H	12.428900	0.419500	1.765600
C	11.908400	1.963000	0.342300
H	11.000200	2.591100	0.266600
N	10.443600	0.884300	2.015500
H	9.758300	1.609700	1.791300
C	10.394900	0.286600	3.221900
O	11.179500	-0.611400	3.546100
C	9.352300	0.777100	4.243000
C	10.145000	1.307600	5.455700
H	9.443300	1.629400	6.244300
H	10.799400	0.518000	5.852500
H	10.774800	2.168400	5.165900
C	8.489300	-0.418300	4.675300
H	7.963100	-0.871200	3.825700
H	7.742700	-0.091200	5.418400
H	9.143800	-1.175000	5.133900
N	8.552400	1.908900	3.754000
H	8.888000	2.811100	4.078000
C	7.268600	1.911000	3.296400
O	6.701000	0.958400	2.781100
C	13.055900	2.776900	0.968600
H	13.959300	2.136000	0.939000
C	6.556700	3.254800	3.577700
N	5.165000	3.227600	3.155600
C	4.278100	2.793200	4.251000
C	5.213300	2.610900	5.453500

C	6.416700	3.488100	5.099900
H	3.519400	3.574200	4.422400
H	3.748500	1.868600	3.976800
H	4.730700	2.904800	6.398700
H	5.530600	1.557100	5.547600
H	7.324200	3.234900	5.668000
H	6.184500	4.553500	5.270000
C	4.611200	3.708600	2.020100
O	3.386000	3.722900	1.880800
H	7.118100	4.062400	3.088700
C	5.543700	4.194900	0.884000
H	6.466600	4.622300	1.298200
C	4.837600	5.250300	0.010200
H	3.914800	4.780900	-0.372900
C	5.704400	5.669900	-1.181800
H	6.624100	6.181400	-0.841200
H	5.142000	6.370800	-1.825100
H	6.004300	4.809600	-1.804100
C	4.437900	6.472900	0.847300
H	5.330000	6.955800	1.290600
H	3.747000	6.197700	1.659700
H	3.929800	7.221100	0.213200
N	5.946400	3.036600	0.095100
H	5.222600	2.627500	-0.492500
C	7.179100	2.465900	0.077600
O	8.139100	2.898700	0.717000
H	12.181000	1.682500	-0.686800
C	13.343800	4.024700	0.127100
H	12.452100	4.679200	0.082000
H	13.618000	3.751500	-0.908600
H	14.174600	4.613200	0.557600
C	12.787400	3.146500	2.434200
H	13.613200	3.757100	2.841700
H	12.690500	2.253100	3.075700
H	11.854300	3.734400	2.530100
H	6.808000	1.438400	-1.774800
H	-9.159200	1.947300	6.160800
H	-6.832600	-3.123100	1.743600
H	-7.304800	6.693900	1.008300
H	5.057700	-5.744700	-1.954600
